# Supplementary material for: Fallacy of attributing the U.S. firearm mortality epidemic to mental health
Source: PLoS One. 2024 Aug 5;19(8):e0290138. doi: 10.1371/journal.pone.0290138 (PMC11299823; doi:10.1371/journal.pone.0290138)
Supplement: S1 Fig — (PDF) [file pone.0290138.s001.pdf]

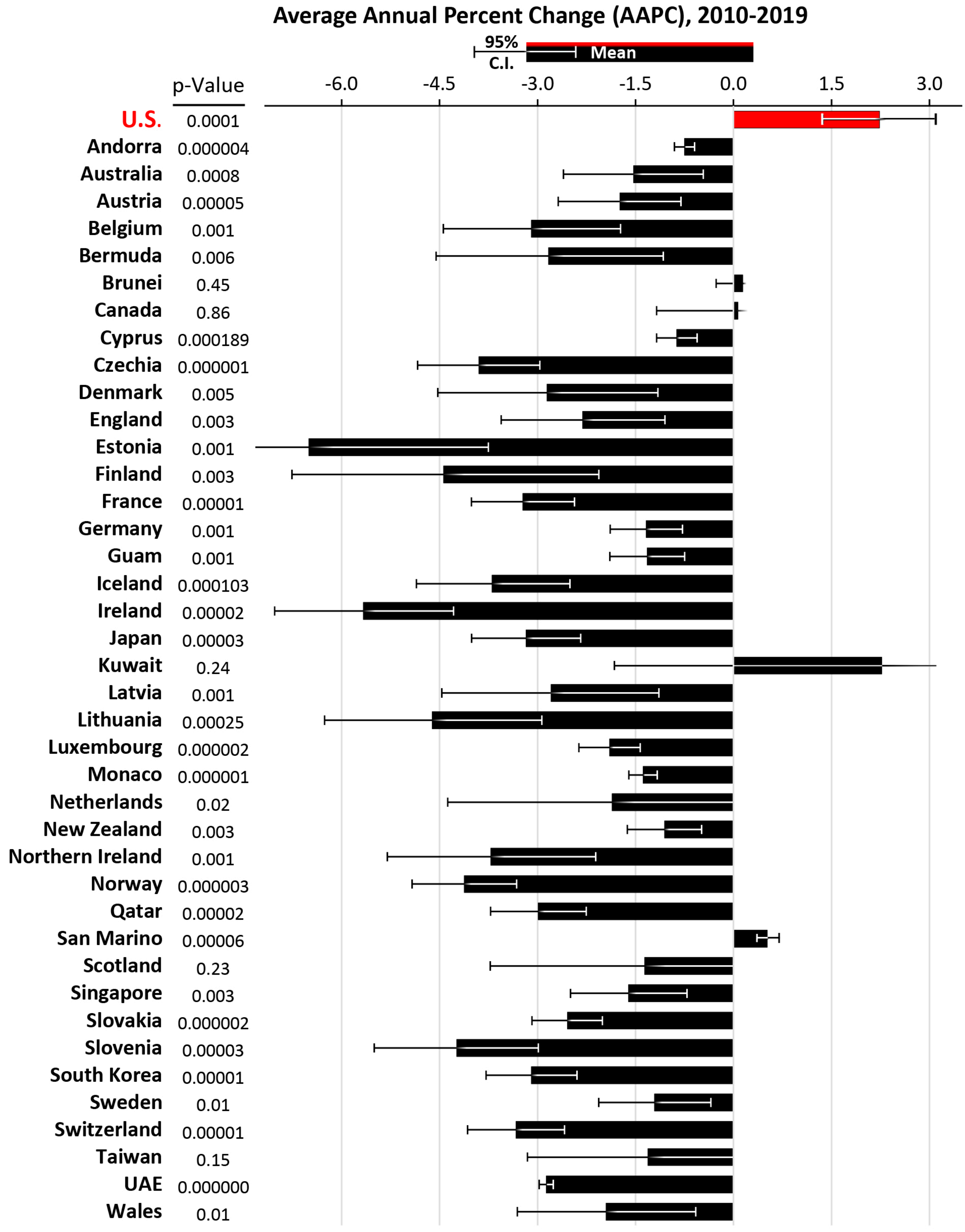

Supplemental Figure S1. **Average Annual Percent Change (AAPC) in the Age-Adjusted Overall Firearm Death Rate, 2010-2019, High SDI Countries, by Country.**

* AAPC – Average Annual Percent Change

Data Sources: CDC WISQARS[6] for U.S.; IHME[4] for other high SDI countries
