# Supplementary material for: Fallacy of attributing the U.S. firearm mortality epidemic to mental health
Source: PLoS One. 2024 Aug 5;19(8):e0290138. doi: 10.1371/journal.pone.0290138 (PMC11299823; doi:10.1371/journal.pone.0290138)
Supplement: S2 Fig — (PDF) [file pone.0290138.s002.pdf]

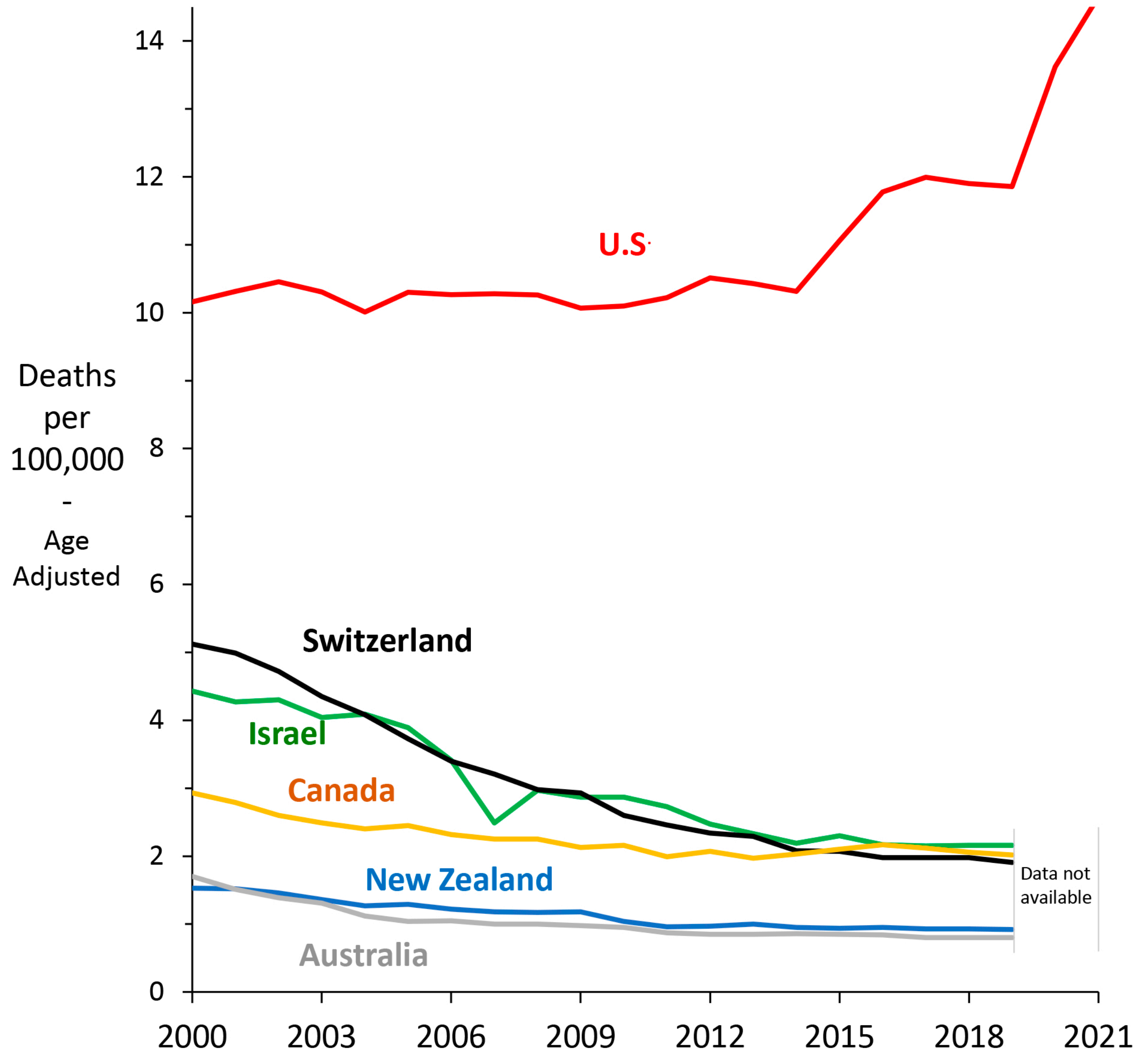

Supplemental Figure S2. **Annual Firearm Death Rate in U.S. during 2000-2021*, and in 5 High SDI Countries during 2000-2019 with National Legislation to Reduce Firearm Violence.**

Data Sources: IHME for Switzerland, Israel, Canada, New Zealand and Australia;
* CDC WISAQRS for U.S., which includes 2020 and 2021
