# Supplementary material for: Fallacy of attributing the U.S. firearm mortality epidemic to mental health
Source: PLoS One. 2024 Aug 5;19(8):e0290138. doi: 10.1371/journal.pone.0290138 (PMC11299823; doi:10.1371/journal.pone.0290138)
Supplement: S1 File — (PDF) [file pone.0290138.s003.pdf]

| | | Andorra | | | Australia | | | Austria | | | Belgium | | | Bermuda | | | Brunei | | | Canada | | | Cyprus | | | Czechia | | | Denma |
|---|---|---|---|---|---|---|---|---|---|---|---|---|---|---|---|---|---|---|---|---|---|---|---|---|---|---|---|---|---|
| | Andorı | AAPC | | Austra | AAPC | | Austri | AAPC | | Belgiu | AAPC | | Bermu | AAPC | | Brunei | AAPC | | Canad | AAPC | | Cyprus | AAPC | | Czechi | AAPC | | Denm | AAPC |
| AAPC 2000-2019 | | -1.7 | ##### | | -3.5 | ##### | | -2.2 | ##### | | -4.7 | ##### | | 0.6 | -100 | | -0.1 | 64.8 | | -1.6 | ##### | | -1.9 | ##### | | -3.5 | ##### | | -4.2 |
| AAPC 2010-2019 | -0.41 | **-0.7** | ##### | -1.31 | **-1.5** | ##### | -3.26 | **-1.7** | ##### | -5.17 | **-3.1** | ##### | -5.23 | **-2.8** | ##### | 0.02 | **0.1** | -99.2 | 0.13 | **0.1** | -48.2 | -1.09 | **-0.9** | ##### | -5.25 | **-3.9** | ##### | -2.75 | **-2.9** |
| | | Ln | | | Ln | | | Ln | | | Ln | | | Ln | | | Ln | | | Ln | | | Ln | | | Ln | | | Ln |
| 2000 | 0.73 | -0.31 | | 1.70 | 0.53 | | 2.86 | 1.05 | | 3.39 | 1.22 | | 1.31 | 0.27 | | 0.14 | -1.97 | | 2.93 | 1.08 | | 1.75 | 0.56 | | 2.17 | 0.77 | | 1.70 | 0.53 |
| 2001 | 0.70 | -0.36 | | 1.51 | 0.41 | | 2.66 | 0.98 | | 3.28 | 1.19 | | 1.45 | 0.37 | | 0.14 | -1.97 | | 2.79 | 1.03 | | 1.72 | 0.54 | | 2.17 | 0.77 | | 1.61 | 0.48 |
| 2002 | 0.68 | -0.39 | | 1.39 | 0.33 | | 2.55 | 0.94 | | 3.13 | 1.14 | | 1.62 | 0.48 | | 0.14 | -1.97 | | 2.60 | 0.96 | | 1.65 | 0.50 | | 2.11 | 0.75 | | 1.61 | 0.48 |
| 2003 | 0.66 | -0.42 | | 1.31 | 0.27 | | 2.41 | 0.88 | | 2.92 | 1.07 | | 1.55 | 0.44 | | 0.14 | -1.97 | | 2.49 | 0.91 | | 1.56 | 0.44 | | 2.15 | 0.77 | | 1.46 | 0.38 |
| 2004 | 0.65 | -0.43 | | 1.12 | 0.11 | | 2.30 | 0.83 | | 2.75 | 1.01 | | 1.62 | 0.48 | | 0.14 | -1.97 | | 2.40 | 0.88 | | 1.52 | 0.42 | | 1.94 | 0.66 | | 1.51 | 0.41 |
| 2005 | 0.63 | -0.46 | | 1.04 | 0.04 | | 2.23 | 0.80 | | 2.55 | 0.94 | | 1.88 | 0.63 | | 0.14 | -1.97 | | 2.45 | 0.90 | | 1.49 | 0.40 | | 1.81 | 0.59 | | 1.35 | 0.30 |
| 2006 | 0.62 | -0.48 | | 1.05 | 0.05 | | 2.10 | 0.74 | | 2.29 | 0.83 | | 1.67 | 0.51 | | 0.14 | -1.97 | | 2.32 | 0.84 | | 1.47 | 0.39 | | 1.76 | 0.57 | | 1.37 | 0.31 |
| 2007 | 0.61 | -0.49 | | 1.00 | 0.00 | | 2.07 | 0.73 | | 2.21 | 0.79 | | 1.69 | 0.52 | | 0.14 | -1.97 | | 2.25 | 0.81 | | 1.44 | 0.36 | | 1.74 | 0.55 | | 1.27 | 0.24 |
| 2008 | 0.59 | -0.53 | | 1.00 | 0.00 | | 2.00 | 0.69 | | 2.16 | 0.77 | | 1.82 | 0.60 | | 0.14 | -1.97 | | 2.25 | 0.81 | | 1.39 | 0.33 | | 1.71 | 0.54 | | 1.21 | 0.19 |
| 2009 | 0.58 | -0.54 | | 0.98 | -0.02 | | 2.13 | 0.76 | | 2.04 | 0.71 | | 2.06 | 0.72 | | 0.14 | -2.00 | | 2.13 | 0.76 | | 1.37 | 0.31 | | 1.62 | 0.48 | | 1.21 | 0.19 |
| 2010 | 0.57 | -0.57 | | 0.95 | -0.05 | | 2.10 | 0.74 | | 1.98 | 0.68 | | 2.21 | 0.79 | | 0.13 | -2.00 | | 2.15 | 0.77 | | 1.31 | 0.27 | | 1.56 | 0.45 | | 1.15 | 0.14 |
| 2011 | 0.56 | -0.58 | | 0.87 | -0.14 | | 1.97 | 0.68 | | 1.89 | 0.64 | | 2.01 | 0.70 | | 0.14 | -2.00 | | 1.99 | 0.69 | | 1.26 | 0.23 | | 1.59 | 0.46 | | 1.06 | 0.06 |
| 2012 | 0.55 | -0.60 | | 0.86 | -0.16 | | 1.93 | 0.66 | | 1.76 | 0.57 | | 1.90 | 0.64 | | 0.13 | -2.01 | | 2.07 | 0.73 | | 1.30 | 0.26 | | 1.53 | 0.42 | | 0.89 | -0.12 |
| 2013 | 0.54 | -0.61 | | 0.84 | -0.17 | | 1.94 | 0.66 | | 1.55 | 0.44 | | 1.88 | 0.63 | | 0.14 | -1.98 | | 1.97 | 0.68 | | 1.30 | 0.26 | | 1.46 | 0.38 | | 0.88 | -0.13 |
| 2014 | 0.55 | -0.61 | | 0.86 | -0.15 | | 1.89 | 0.64 | | 1.58 | 0.46 | | 1.59 | 0.46 | | 0.14 | -1.97 | | 2.03 | 0.71 | | 1.27 | 0.24 | | 1.36 | 0.31 | | 0.88 | -0.13 |
| 2015 | 0.54 | -0.62 | | 0.85 | -0.17 | | 1.91 | 0.65 | | 1.53 | 0.43 | | 1.61 | 0.48 | | 0.14 | -1.96 | | 2.10 | 0.74 | | 1.26 | 0.23 | | 1.31 | 0.27 | | 0.84 | -0.17 |
| 2016 | 0.54 | -0.62 | | 0.84 | -0.17 | | 1.88 | 0.63 | | 1.46 | 0.38 | | 1.63 | 0.49 | | 0.14 | -1.98 | | 2.17 | 0.78 | | 1.24 | 0.22 | | 1.22 | 0.20 | | 0.84 | -0.17 |
| 2017 | 0.53 | -0.63 | | 0.79 | -0.23 | | 1.84 | 0.61 | | 1.51 | 0.41 | | 1.67 | 0.51 | | 0.14 | -1.98 | | 2.12 | 0.75 | | 1.23 | 0.21 | | 1.21 | 0.19 | | 0.86 | -0.16 |
| 2018 | 0.53 | -0.63 | | 0.79 | -0.23 | | 1.77 | 0.57 | | 1.50 | 0.41 | | 1.68 | 0.52 | | 0.14 | -1.99 | | 2.06 | 0.72 | | 1.22 | 0.20 | | 1.20 | 0.18 | | 0.85 | -0.16 |
| 2019 | 0.53 | -0.64 | | 0.80 | -0.23 | | 1.71 | 0.54 | | 1.49 | 0.40 | | 1.68 | 0.52 | | 0.14 | -2.00 | | 2.02 | 0.70 | | 1.20 | 0.18 | | 1.14 | 0.13 | | 0.83 | -0.19 |

| Year | cause | val | upper | lower | val | upper | lower | val | upper | lower | val | upper | lower | val | upper | lower | val | upper | lower | val | upper | lower | val | upper | lower | val | upper | lower | val | upper |
|---|---|---|---|---|---|---|---|---|---|---|---|---|---|---|---|---|---|---|---|---|---|---|---|---|---|---|---|---|---|---|
| 2000 | Physical violence by firearm | 0.12 | 0.16 | 0.08 | 0.32 | 0.34 | 0.30 | 0.24 | 0.25 | 0.22 | 0.75 | 0.80 | 0.71 | 1.02 | 1.14 | 0.90 | 0.08 | 0.10 | 0.06 | 0.48 | 0.51 | 0.45 | 0.74 | 1.00 | 0.58 | 0.42 | 0.44 | 0.39 | 0.31 | 0.33 |
| 2001 | Physical violence by firearm | 0.11 | 0.16 | 0.08 | 0.26 | 0.28 | 0.24 | 0.19 | 0.20 | 0.17 | 0.71 | 0.75 | 0.67 | 1.14 | 1.28 | 1.00 | 0.08 | 0.10 | 0.06 | 0.49 | 0.52 | 0.46 | 0.75 | 0.98 | 0.58 | 0.41 | 0.43 | 0.39 | 0.31 | 0.33 |
| 2002 | Physical violence by firearm | 0.11 | 0.15 | 0.07 | 0.25 | 0.26 | 0.23 | 0.17 | 0.18 | 0.16 | 0.69 | 0.73 | 0.65 | 1.31 | 1.48 | 1.15 | 0.08 | 0.10 | 0.06 | 0.46 | 0.49 | 0.44 | 0.72 | 0.94 | 0.58 | 0.41 | 0.44 | 0.39 | 0.27 | 0.29 |
| 2003 | Physical violence by firearm | 0.10 | 0.15 | 0.07 | 0.24 | 0.25 | 0.22 | 0.17 | 0.18 | 0.16 | 0.63 | 0.66 | 0.59 | 1.23 | 1.41 | 1.07 | 0.07 | 0.10 | 0.06 | 0.45 | 0.48 | 0.43 | 0.69 | 0.90 | 0.57 | 0.40 | 0.42 | 0.37 | 0.19 | 0.21 |
| 2004 | Physical violence by firearm | 0.10 | 0.14 | 0.07 | 0.14 | 0.15 | 0.13 | 0.15 | 0.16 | 0.14 | 0.65 | 0.68 | 0.61 | 1.30 | 1.50 | 1.13 | 0.07 | 0.10 | 0.06 | 0.50 | 0.53 | 0.47 | 0.67 | 0.88 | 0.56 | 0.29 | 0.30 | 0.27 | 0.25 | 0.26 |
| 2005 | Physical violence by firearm | 0.10 | 0.14 | 0.07 | 0.13 | 0.14 | 0.12 | 0.15 | 0.16 | 0.14 | 0.53 | 0.56 | 0.50 | 1.59 | 1.81 | 1.39 | 0.07 | 0.09 | 0.06 | 0.62 | 0.65 | 0.59 | 0.67 | 0.87 | 0.57 | 0.25 | 0.26 | 0.23 | 0.21 | 0.23 |
| 2006 | Physical violence by firearm | 0.10 | 0.14 | 0.07 | 0.19 | 0.21 | 0.17 | 0.13 | 0.14 | 0.12 | 0.44 | 0.47 | 0.41 | 1.40 | 1.57 | 1.23 | 0.07 | 0.09 | 0.05 | 0.60 | 0.63 | 0.56 | 0.65 | 0.84 | 0.55 | 0.29 | 0.31 | 0.28 | 0.24 | 0.26 |
| 2007 | Physical violence by firearm | 0.10 | 0.13 | 0.06 | 0.16 | 0.18 | 0.15 | 0.15 | 0.16 | 0.14 | 0.44 | 0.47 | 0.41 | 1.41 | 1.60 | 1.25 | 0.07 | 0.09 | 0.06 | 0.56 | 0.59 | 0.53 | 0.61 | 0.80 | 0.52 | 0.27 | 0.29 | 0.25 | 0.19 | 0.20 |
| 2008 | Physical violence by firearm | 0.09 | 0.13 | 0.06 | 0.16 | 0.18 | 0.15 | 0.15 | 0.17 | 0.14 | 0.37 | 0.40 | 0.35 | 1.55 | 1.74 | 1.37 | 0.07 | 0.09 | 0.06 | 0.63 | 0.67 | 0.59 | 0.59 | 0.76 | 0.51 | 0.28 | 0.30 | 0.26 | 0.18 | 0.20 |
| 2009 | Physical violence by firearm | 0.09 | 0.13 | 0.06 | 0.19 | 0.20 | 0.17 | 0.19 | 0.20 | 0.18 | 0.38 | 0.41 | 0.36 | 1.81 | 2.03 | 1.61 | 0.07 | 0.09 | 0.05 | 0.55 | 0.58 | 0.52 | 0.58 | 0.74 | 0.49 | 0.25 | 0.27 | 0.23 | 0.20 | 0.22 |
| 2010 | Physical violence by firearm | 0.09 | 0.12 | 0.06 | 0.19 | 0.21 | 0.18 | 0.15 | 0.16 | 0.14 | 0.42 | 0.45 | 0.39 | 1.97 | 2.22 | 1.75 | 0.07 | 0.09 | 0.05 | 0.60 | 0.63 | 0.56 | 0.57 | 0.69 | 0.49 | 0.19 | 0.21 | 0.18 | 0.19 | 0.21 |
| 2011 | Physical violence by firearm | 0.09 | 0.12 | 0.06 | 0.17 | 0.18 | 0.15 | 0.11 | 0.12 | 0.11 | 0.40 | 0.42 | 0.37 | 1.79 | 2.01 | 1.58 | 0.07 | 0.09 | 0.05 | 0.51 | 0.54 | 0.48 | 0.55 | 0.66 | 0.47 | 0.21 | 0.22 | 0.19 | 0.20 | 0.21 |
| 2012 | Physical violence by firearm | 0.09 | 0.12 | 0.05 | 0.18 | 0.20 | 0.17 | 0.10 | 0.11 | 0.09 | 0.33 | 0.36 | 0.31 | 1.69 | 1.91 | 1.49 | 0.07 | 0.09 | 0.05 | 0.56 | 0.60 | 0.53 | 0.58 | 0.69 | 0.49 | 0.20 | 0.21 | 0.18 | 0.10 | 0.11 |
| 2013 | Physical violence by firearm | 0.08 | 0.12 | 0.05 | 0.18 | 0.19 | 0.17 | 0.14 | 0.15 | 0.13 | 0.27 | 0.28 | 0.25 | 1.68 | 1.90 | 1.46 | 0.07 | 0.09 | 0.05 | 0.47 | 0.50 | 0.44 | 0.56 | 0.67 | 0.48 | 0.18 | 0.19 | 0.16 | 0.12 | 0.13 |
| 2014 | Physical violence by firearm | 0.09 | 0.12 | 0.05 | 0.18 | 0.19 | 0.17 | 0.13 | 0.14 | 0.12 | 0.37 | 0.39 | 0.34 | 1.39 | 1.59 | 1.21 | 0.07 | 0.09 | 0.06 | 0.48 | 0.51 | 0.44 | 0.54 | 0.66 | 0.47 | 0.17 | 0.18 | 0.15 | 0.14 | 0.15 |
| 2015 | Physical violence by firearm | 0.08 | 0.12 | 0.05 | 0.15 | 0.16 | 0.14 | 0.12 | 0.13 | 0.11 | 0.34 | 0.36 | 0.32 | 1.42 | 1.64 | 1.22 | 0.08 | 0.10 | 0.06 | 0.51 | 0.55 | 0.47 | 0.54 | 0.66 | 0.47 | 0.16 | 0.17 | 0.15 | 0.17 | 0.18 |
| 2016 | Physical violence by firearm | 0.08 | 0.12 | 0.05 | 0.19 | 0.21 | 0.18 | 0.15 | 0.16 | 0.14 | 0.29 | 0.31 | 0.27 | 1.45 | 1.67 | 1.24 | 0.08 | 0.10 | 0.06 | 0.55 | 0.60 | 0.49 | 0.54 | 0.66 | 0.46 | 0.12 | 0.13 | 0.11 | 0.15 | 0.16 |
| 2017 | Physical violence by firearm | 0.08 | 0.12 | 0.05 | 0.18 | 0.19 | 0.17 | 0.13 | 0.14 | 0.12 | 0.32 | 0.35 | 0.30 | 1.49 | 1.73 | 1.25 | 0.08 | 0.10 | 0.06 | 0.52 | 0.57 | 0.47 | 0.52 | 0.64 | 0.44 | 0.12 | 0.13 | 0.11 | 0.14 | 0.16 |
| 2018 | Physical violence by firearm | 0.08 | 0.12 | 0.05 | 0.18 | 0.19 | 0.16 | 0.13 | 0.14 | 0.12 | 0.33 | 0.36 | 0.31 | 1.50 | 1.77 | 1.24 | 0.07 | 0.09 | 0.06 | 0.50 | 0.55 | 0.45 | 0.52 | 0.64 | 0.42 | 0.14 | 0.17 | 0.12 | 0.15 | 0.16 |
| 2019 | Physical violence by firearm | 0.08 | 0.12 | 0.05 | 0.18 | 0.19 | 0.16 | 0.13 | 0.14 | 0.12 | 0.34 | 0.36 | 0.31 | 1.50 | 1.81 | 1.23 | 0.07 | 0.09 | 0.05 | 0.50 | 0.55 | 0.45 | 0.51 | 0.64 | 0.41 | 0.15 | 0.18 | 0.12 | 0.15 | 0.16 |
| 2000 | Self-harm by firearm | 0.59 | 2.02 | 0.25 | 1.22 | 1.66 | 1.10 | 2.58 | 2.97 | 2.04 | 2.56 | 3.13 | 2.12 | 0.06 | 0.08 | 0.05 | 0.04 | 0.07 | 0.03 | 2.34 | 2.99 | 2.08 | 0.78 | 1.25 | 0.48 | 1.62 | 1.98 | 1.29 | 1.34 | 1.78 |
| 2001 | Self-harm by firearm | 0.57 | 1.97 | 0.24 | 1.11 | 1.49 | 0.99 | 2.43 | 2.76 | 1.90 | 2.49 | 3.05 | 2.06 | 0.06 | 0.07 | 0.05 | 0.04 | 0.07 | 0.03 | 2.19 | 2.80 | 1.93 | 0.76 | 1.19 | 0.47 | 1.63 | 1.99 | 1.29 | 1.25 | 1.64 |
| 2002 | Self-harm by firearm | 0.55 | 1.93 | 0.23 | 1.00 | 1.33 | 0.87 | 2.34 | 2.66 | 1.81 | 2.36 | 2.85 | 1.95 | 0.06 | 0.07 | 0.04 | 0.04 | 0.07 | 0.03 | 2.04 | 2.64 | 1.78 | 0.74 | 1.13 | 0.44 | 1.58 | 1.91 | 1.25 | 1.28 | 1.64 |
| 2003 | Self-harm by firearm | 0.54 | 1.90 | 0.23 | 0.93 | 1.21 | 0.79 | 2.20 | 2.47 | 1.65 | 2.22 | 2.66 | 1.82 | 0.06 | 0.07 | 0.04 | 0.05 | 0.07 | 0.03 | 1.95 | 2.53 | 1.68 | 0.69 | 1.04 | 0.42 | 1.64 | 2.00 | 1.30 | 1.22 | 1.53 |
| 2004 | Self-harm by firearm | 0.53 | 1.88 | 0.22 | 0.85 | 1.11 | 0.72 | 2.12 | 2.38 | 1.57 | 2.03 | 2.44 | 1.66 | 0.06 | 0.07 | 0.04 | 0.05 | 0.07 | 0.03 | 1.81 | 2.33 | 1.55 | 0.68 | 1.01 | 0.40 | 1.53 | 1.87 | 1.21 | 1.21 | 1.46 |
| 2005 | Self-harm by firearm | 0.51 | 1.78 | 0.22 | 0.80 | 1.05 | 0.67 | 2.05 | 2.29 | 1.49 | 1.95 | 2.34 | 1.58 | 0.05 | 0.07 | 0.04 | 0.05 | 0.07 | 0.03 | 1.75 | 2.25 | 1.48 | 0.67 | 0.96 | 0.38 | 1.44 | 1.76 | 1.13 | 1.09 | 1.29 |
| 2006 | Self-harm by firearm | 0.50 | 1.74 | 0.21 | 0.77 | 1.01 | 0.64 | 1.94 | 2.15 | 1.39 | 1.79 | 2.16 | 1.45 | 0.05 | 0.06 | 0.04 | 0.05 | 0.07 | 0.03 | 1.65 | 2.12 | 1.39 | 0.69 | 0.91 | 0.37 | 1.36 | 1.68 | 1.05 | 1.08 | 1.28 |
| 2007 | Self-harm by firearm | 0.49 | 1.71 | 0.21 | 0.77 | 1.03 | 0.65 | 1.89 | 2.11 | 1.33 | 1.71 | 2.10 | 1.39 | 0.05 | 0.06 | 0.04 | 0.05 | 0.07 | 0.03 | 1.62 | 2.09 | 1.36 | 0.72 | 0.91 | 0.35 | 1.35 | 1.65 | 1.03 | 1.03 | 1.21 |
| 2008 | Self-harm by firearm | 0.48 | 1.70 | 0.20 | 0.77 | 1.04 | 0.64 | 1.81 | 2.02 | 1.28 | 1.73 | 2.14 | 1.42 | 0.05 | 0.06 | 0.04 | 0.05 | 0.07 | 0.03 | 1.55 | 2.03 | 1.31 | 0.71 | 0.87 | 0.33 | 1.31 | 1.63 | 1.00 | 0.99 | 1.15 |
| 2009 | Self-harm by firearm | 0.47 | 1.67 | 0.19 | 0.73 | 0.99 | 0.62 | 1.90 | 2.12 | 1.34 | 1.60 | 2.04 | 1.31 | 0.05 | 0.06 | 0.03 | 0.04 | 0.07 | 0.03 | 1.52 | 2.05 | 1.29 | 0.71 | 0.86 | 0.31 | 1.26 | 1.60 | 0.95 | 0.98 | 1.16 |
| 2010 | Self-harm by firearm | 0.46 | 1.63 | 0.19 | 0.70 | 0.95 | 0.60 | 1.91 | 2.13 | 1.34 | 1.50 | 1.94 | 1.22 | 0.05 | 0.06 | 0.03 | 0.05 | 0.07 | 0.03 | 1.50 | 2.00 | 1.27 | 0.67 | 0.82 | 0.29 | 1.27 | 1.59 | 0.95 | 0.93 | 1.11 |
| 2011 | Self-harm by firearm | 0.45 | 1.61 | 0.18 | 0.65 | 0.91 | 0.55 | 1.82 | 2.02 | 1.27 | 1.45 | 1.88 | 1.18 | 0.05 | 0.06 | 0.03 | 0.05 | 0.07 | 0.03 | 1.42 | 1.91 | 1.20 | 0.66 | 0.80 | 0.27 | 1.28 | 1.63 | 0.96 | 0.84 | 1.02 |
| 2012 | Self-harm by firearm | 0.45 | 1.60 | 0.18 | 0.63 | 0.89 | 0.54 | 1.80 | 2.00 | 1.25 | 1.38 | 1.80 | 1.12 | 0.04 | 0.05 | 0.03 | 0.05 | 0.07 | 0.03 | 1.46 | 1.96 | 1.24 | 0.67 | 0.83 | 0.26 | 1.24 | 1.56 | 0.91 | 0.76 | 0.98 |
| 2013 | Self-harm by firearm | 0.44 | 1.57 | 0.17 | 0.63 | 0.90 | 0.54 | 1.76 | 1.97 | 1.23 | 1.24 | 1.62 | 1.00 | 0.04 | 0.05 | 0.03 | 0.05 | 0.07 | 0.03 | 1.45 | 1.97 | 1.25 | 0.68 | 0.84 | 0.25 | 1.20 | 1.48 | 0.87 | 0.73 | 0.95 |
| 2014 | Self-harm by firearm | 0.44 | 1.57 | 0.17 | 0.64 | 0.93 | 0.56 | 1.72 | 1.93 | 1.21 | 1.16 | 1.52 | 0.92 | 0.04 | 0.05 | 0.03 | 0.05 | 0.07 | 0.03 | 1.50 | 2.03 | 1.29 | 0.67 | 0.83 | 0.25 | 1.11 | 1.35 | 0.79 | 0.72 | 0.91 |
| 2015 | Self-harm by firearm | 0.44 | 1.59 | 0.16 | 0.66 | 0.97 | 0.58 | 1.75 | 1.96 | 1.22 | 1.14 | 1.49 | 0.89 | 0.04 | 0.05 | 0.03 | 0.05 | 0.07 | 0.03 | 1.54 | 2.08 | 1.30 | 0.66 | 0.82 | 0.24 | 1.07 | 1.28 | 0.75 | 0.65 | 0.81 |
| 2016 | Self-harm by firearm | 0.43 | 1.59 | 0.16 | 0.61 | 0.89 | 0.53 | 1.69 | 1.91 | 1.16 | 1.12 | 1.46 | 0.88 | 0.04 | 0.05 | 0.03 | 0.05 | 0.07 | 0.03 | 1.57 | 2.09 | 1.33 | 0.64 | 0.80 | 0.24 | 1.02 | 1.22 | 0.71 | 0.68 | 0.84 |
| 2017 | Self-harm by firearm | 0.43 | 1.60 | 0.16 | 0.58 | 0.83 | 0.50 | 1.68 | 1.90 | 1.15 | 1.13 | 1.47 | 0.90 | 0.04 | 0.05 | 0.03 | 0.05 | 0.07 | 0.03 | 1.55 | 2.04 | 1.32 | 0.64 | 0.81 | 0.24 | 1.01 | 1.21 | 0.71 | 0.69 | 0.85 |
| 2018 | Self-harm by firearm | 0.43 | 1.58 | 0.16 | 0.58 | 0.83 | 0.49 | 1.61 | 1.86 | 1.11 | 1.13 | 1.46 | 0.89 | 0.04 | 0.05 | 0.03 | 0.05 | 0.07 | 0.03 | 1.51 | 1.97 | 1.28 | 0.64 | 0.81 | 0.24 | 0.98 | 1.21 | 0.69 | 0.68 | 0.84 |
| 2019 | Self-harm by firearm | 0.42 | 1.55 | 0.15 | 0.58 | 0.83 | 0.49 | 1.55 | 1.81 | 1.06 | 1.10 | 1.43 | 0.88 | 0.04 | 0.05 | 0.03 | 0.05 | 0.07 | 0.03 | 1.47 | 1.90 | 1.24 | 0.64 | 0.80 | 0.25 | 0.92 | 1.23 | 0.63 | 0.66 | 0.81 |
| 2000 | Unintentional firearm injuries | 0.02 | 0.05 | 0.01 | 0.16 | 0.18 | 0.09 | 0.04 | 0.07 | 0.03 | 0.08 | 0.10 | 0.06 | 0.23 | 0.28 | 0.19 | 0.02 | 0.03 | 0.01 | 0.11 | 0.13 | 0.08 | 0.23 | 0.32 | 0.09 | 0.13 | 0.15 | 0.09 | 0.05 | 0.07 |
| 2001 | Unintentional firearm injuries | 0.02 | 0.05 | 0.01 | 0.14 | 0.16 | 0.09 | 0.04 | 0.06 | 0.03 | 0.08 | 0.10 | 0.06 | 0.25 | 0.30 | 0.19 | 0.02 | 0.03 | 0.01 | 0.11 | 0.12 | 0.08 | 0.21 | 0.29 | 0.08 | 0.13 | 0.15 | 0.09 | 0.05 | 0.07 |
| 2002 | Unintentional firearm injuries | 0.02 | 0.05 | 0.01 | 0.14 | 0.16 | 0.08 | 0.04 | 0.06 | 0.03 | 0.08 | 0.09 | 0.06 | 0.25 | 0.30 | 0.19 | 0.02 | 0.03 | 0.01 | 0.10 | 0.12 | 0.07 | 0.19 | 0.26 | 0.08 | 0.12 | 0.14 | 0.09 | 0.06 | 0.07 |
| 2003 | Unintentional firearm injuries | 0.02 | 0.05 | 0.01 | 0.14 | 0.16 | 0.08 | 0.04 | 0.06 | 0.03 | 0.07 | 0.09 | 0.06 | 0.26 | 0.31 | 0.20 | 0.02 | 0.03 | 0.01 | 0.09 | 0.11 | 0.07 | 0.18 | 0.23 | 0.07 | 0.11 | 0.14 | 0.08 | 0.05 | 0.07 |
| 2004 | Unintentional firearm injuries | 0.02 | 0.05 | 0.01 | 0.13 | 0.15 | 0.08 | 0.03 | 0.06 | 0.03 | 0.07 | 0.08 | 0.05 | 0.26 | 0.31 | 0.19 | 0.02 | 0.03 | 0.01 | 0.09 | 0.11 | 0.07 | 0.17 | 0.22 | 0.07 | 0.12 | 0.14 | 0.08 | 0.05 | 0.07 |
| 2005 | Unintentional firearm injuries | 0.02 | 0.05 | 0.01 | 0.11 | 0.13 | 0.07 | 0.03 | 0.06 | 0.03 | 0.07 | 0.08 | 0.05 | 0.24 | 0.29 | 0.17 | 0.02 | 0.03 | 0.01 | 0.08 | 0.11 | 0.06 | 0.15 | 0.19 | 0.06 | 0.12 | 0.14 | 0.08 | 0.05 | 0.06 |
| 2006 | Unintentional firearm injuries | 0.02 | 0.05 | 0.01 | 0.09 | 0.10 | 0.06 | 0.03 | 0.05 | 0.03 | 0.06 | 0.08 | 0.05 | 0.22 | 0.26 | 0.16 | 0.02 | 0.03 | 0.01 | 0.07 | 0.10 | 0.06 | 0.13 | 0.16 | 0.06 | 0.11 | 0.13 | 0.07 | 0.05 | 0.06 |
| 2007 | Unintentional firearm injuries | 0.02 | 0.05 | 0.01 | 0.07 | 0.09 | 0.06 | 0.03 | 0.05 | 0.03 | 0.06 | 0.07 | 0.05 | 0.23 | 0.27 | 0.16 | 0.02 | 0.02 | 0.01 | 0.07 | 0.10 | 0.06 | 0.11 | 0.14 | 0.06 | 0.12 | 0.14 | 0.08 | 0.05 | 0.06 |
| 2008 | Unintentional firearm injuries | 0.02 | 0.05 | 0.01 | 0.07 | 0.09 | 0.06 | 0.04 | 0.05 | 0.03 | 0.06 | 0.07 | 0.05 | 0.22 | 0.26 | 0.15 | 0.02 | 0.02 | 0.01 | 0.07 | 0.09 | 0.06 | 0.09 | 0.12 | 0.05 | 0.12 | 0.13 | 0.07 | 0.04 | 0.05 |
| 2009 | Unintentional firearm injuries | 0.02 | 0.05 | 0.01 | 0.06 | 0.09 | 0.05 | 0.04 | 0.05 | 0.03 | 0.05 | 0.06 | 0.04 | 0.20 | 0.24 | 0.14 | 0.02 | 0.02 | 0.01 | 0.06 | 0.09 | 0.05 | 0.08 | 0.10 | 0.05 | 0.11 | 0.13 | 0.07 | 0.03 | 0.05 |
| 2010 | Unintentional firearm injuries | 0.02 | 0.04 | 0.01 | 0.06 | 0.08 | 0.05 | 0.04 | 0.05 | 0.03 | 0.05 | 0.06 | 0.04 | 0.18 | 0.22 | 0.14 | 0.02 | 0.02 | 0.01 | 0.06 | 0.08 | 0.05 | 0.07 | 0.09 | 0.05 | 0.11 | 0.12 | 0.07 | 0.03 | 0.04 |
| 2011 | Unintentional firearm injuries | 0.02 | 0.04 | 0.01 | 0.05 | 0.08 | 0.04 | 0.03 | 0.05 | 0.03 | 0.05 | 0.06 | 0.04 | 0.17 | 0.20 | 0.13 | 0.02 | 0.02 | 0.01 | 0.06 | 0.08 | 0.05 | 0.06 | 0.09 | 0.04 | 0.10 | 0.12 | 0.06 | 0.02 | 0.04 |
| 2012 | Unintentional firearm injuries | 0.02 | 0.04 | 0.01 | 0.04 | 0.07 | 0.04 | 0.03 | 0.04 | 0.03 | 0.05 | 0.06 | 0.04 | 0.16 | 0.19 | 0.13 | 0.02 | 0.02 | 0.01 | 0.05 | 0.08 | 0.05 | 0.06 | 0.09 | 0.04 | 0.09 | 0.11 | 0.06 | 0.02 | 0.04 |
| 2013 | Unintentional firearm injuries | 0.02 | 0.04 | 0.01 | 0.04 | 0.07 | 0.03 | 0.04 | 0.04 | 0.03 | 0.05 | 0.06 | 0.04 | 0.16 | 0.19 | 0.13 | 0.02 | 0.02 | 0.01 | 0.05 | 0.08 | 0.04 | 0.05 | 0.09 | 0.04 | 0.09 | 0.10 | 0.06 | 0.02 | 0.04 |
| 2014 | Unintentional firearm injuries | 0.02 | 0.04 | 0.01 | 0.04 | 0.07 | 0.03 | 0.04 | 0.04 | 0.03 | 0.05 | 0.06 | 0.04 | 0.16 | 0.20 | 0.13 | 0.02 | 0.02 | 0.01 | 0.05 | 0.08 | 0.04 | 0.06 | 0.09 | 0.04 | 0.08 | 0.10 | 0.06 | 0.02 | 0.04 |
| 2015 | Unintentional firearm injuries | 0.02 | 0.04 | 0.01 | 0.04 | 0.07 | 0.03 | 0.04 | 0.04 | 0.03 | 0.05 | 0.06 | 0.04 | 0.15 | 0.19 | 0.12 | 0.02 | 0.02 | 0.01 | 0.05 | 0.08 | 0.04 | 0.06 | 0.09 | 0.04 | 0.08 | 0.10 | 0.06 | 0.02 | 0.04 |
| 2016 | Unintentional firearm injuries | 0.02 | 0.04 | 0.01 | 0.04 | 0.07 | 0.03 | 0.04 | 0.04 | 0.02 | 0.05 | 0.06 | 0.04 | 0.14 | 0.19 | 0.11 | 0.02 | 0.02 | 0.01 | 0.05 | 0.08 | 0.04 | 0.06 | 0.09 | 0.04 | 0.08 | 0.09 | 0.06 | 0.02 | 0.04 |
| 2017 | Unintentional firearm injuries | 0.02 | 0.04 | 0.01 | 0.04 | 0.07 | 0.03 | 0.04 | 0.04 | 0.02 | 0.05 | 0.06 | 0.03 | 0.15 | 0.20 | 0.11 | 0.02 | 0.02 | 0.01 | 0.05 | 0.08 | 0.04 | 0.06 | 0.09 | 0.04 | 0.07 | 0.09 | 0.06 | 0.02 | 0.04 |
| 2018 | Unintentional firearm injuries | 0.02 | 0.04 | 0.01 | 0.04 | 0.06 | 0.03 | 0.03 | 0.04 | 0.02 | 0.05 | 0.06 | 0.03 | 0.15 | 0.20 | 0.11 | 0.02 | 0.02 | 0.01 | 0.05 | 0.08 | 0.04 | 0.06 | 0.09 | 0.04 | 0.07 | 0.09 | 0.06 | 0.02 | 0.04 |
| 2019 | Unintentional firearm injuries | 0.02 | 0.04 | 0.01 | 0.04 | 0.06 | 0.03 | 0.03 | 0.04 | 0.02 | 0.05 | 0.06 | 0.03 | 0.15 | 0.20 | 0.11 | 0.02 | 0.02 | 0.01 | 0.05 | 0.08 | 0.04 | 0.06 | 0.09 | 0.04 | 0.07 | 0.09 | 0.05 | 0.02 | 0.04 |

| ark | | England | | | Estonia | | | Finland | | | France | | | Germany | | | Guam | | | Iceland | | | Ireland | | | Japan | | | Kuwait | | | Latvia | | | Lithuan |
|---|---|---|---|---|---|---|---|---|---|---|---|---|---|---|---|---|---|---|---|---|---|---|---|---|---|---|---|---|---|---|---|---|---|---|---|
| | Englan | AAPC | | Estoni | AAPC | | Finlanc | AAPC | | France | AAPC | | Germa | AAPC | | Guam | AAPC | | Icelanc | AAPC | | Irelanc | AAPC | | Japan | AAPC | | Kuwait | AAPC | | Latvia | AAPC | | Lithuan | AAPC |
| ##### | | -3.8 | ##### | | -7.7 | ##### | | -4.8 | ##### | | -3.5 | ##### | | -2.8 | ##### | | -2.2 | ##### | | -3.1 | ##### | | -4.5 | ##### | | -4.0 | ##### | | -8.4 | ##### | | -5.5 | ##### | | -5.1 |
| ##### | -0.46 | **-2.3** | ##### | -8.55 | **-6.5** | ##### | -10.9 | **-4.4** | ##### | -7.79 | **-3.2** | ##### | -1.01 | **-1.3** | ##### | -2.88 | **-1.3** | ##### | -4.02 | **-3.7** | ##### | -4.02 | **-5.7** | ##### | -0.2 | **-3.2** | ##### | 0.66 | **2.3** | -100 | -3.49 | **-2.8** | ##### | -4.91 | **-4.6** |
| | | Ln | | | Ln | | | Ln | | | Ln | | | Ln | | | Ln | | | Ln | | | Ln | | | Ln | | | Ln | | | Ln | | | Ln |
| | 0.32 | -1.14 | | 4.36 | 1.47 | | 5.13 | 1.64 | | 4.29 | 1.46 | | 1.18 | 0.17 | | 3.32 | 1.20 | | 1.85 | 0.62 | | 1.35 | 0.30 | | 0.11 | -2.21 | | 0.89 | -0.12 | | 3.11 | 1.13 | | 2.63 | 0.97 |
| | 0.34 | -1.08 | | 3.95 | 1.37 | | 4.81 | 1.57 | | 4.14 | 1.42 | | 1.11 | 0.10 | | 3.09 | 1.13 | | 1.73 | 0.55 | | 1.17 | 0.16 | | 0.11 | -2.21 | | 1.03 | 0.03 | | 3.27 | 1.18 | | 2.25 | 0.81 |
| | 0.33 | -1.11 | | 3.70 | 1.31 | | 4.29 | 1.46 | | 3.92 | 1.37 | | 1.10 | 0.10 | | 2.84 | 1.04 | | 1.59 | 0.46 | | 1.19 | 0.17 | | 0.11 | -2.21 | | 0.76 | -0.27 | | 2.71 | 1.00 | | 1.95 | 0.67 |
| | 0.31 | -1.17 | | 3.15 | 1.15 | | 4.09 | 1.41 | | 3.71 | 1.31 | | 1.09 | 0.09 | | 2.84 | 1.04 | | 1.51 | 0.41 | | 1.12 | 0.11 | | 0.10 | -2.30 | | 1.80 | 0.59 | | 2.31 | 0.84 | | 1.85 | 0.62 |
| | 0.29 | -1.24 | | 2.60 | 0.96 | | 3.93 | 1.37 | | 3.32 | 1.20 | | 1.03 | 0.03 | | 2.67 | 0.98 | | 1.47 | 0.39 | | 1.02 | 0.02 | | 0.10 | -2.30 | | 2.55 | 0.94 | | 1.99 | 0.69 | | 1.80 | 0.59 |
| | 0.28 | -1.27 | | 2.42 | 0.88 | | 3.49 | 1.25 | | 3.19 | 1.16 | | 0.96 | -0.04 | | 2.63 | 0.97 | | 1.41 | 0.34 | | 0.98 | -0.02 | | 0.09 | -2.41 | | 1.11 | 0.10 | | 2.15 | 0.77 | | 1.82 | 0.60 |
| | 0.28 | -1.27 | | 2.39 | 0.87 | | 3.53 | 1.26 | | 3.05 | 1.12 | | 0.89 | -0.12 | | 2.58 | 0.95 | | 1.41 | 0.34 | | 1.00 | 0.00 | | 0.09 | -2.41 | | 0.75 | -0.29 | | 2.25 | 0.81 | | 1.75 | 0.56 |
| | 0.25 | -1.39 | | 2.30 | 0.83 | | 3.59 | 1.28 | | 2.95 | 1.08 | | 0.87 | -0.14 | | 2.48 | 0.91 | | 1.37 | 0.31 | | 0.98 | -0.02 | | 0.08 | -2.53 | | 0.60 | -0.51 | | 2.03 | 0.71 | | 1.79 | 0.58 |
| | 0.26 | -1.35 | | 1.92 | 0.65 | | 3.58 | 1.28 | | 2.95 | 1.08 | | 0.84 | -0.17 | | 2.47 | 0.90 | | 1.34 | 0.29 | | 1.00 | 0.00 | | 0.08 | -2.53 | | 0.59 | -0.53 | | 1.85 | 0.62 | | 1.61 | 0.48 |
| | 0.23 | -1.45 | | 2.01 | 0.70 | | 3.39 | 1.22 | | 2.95 | 1.08 | | 0.84 | -0.17 | | 2.46 | 0.90 | | 1.32 | 0.27 | | 1.11 | 0.11 | | 0.08 | -2.57 | | 0.45 | -0.81 | | 1.44 | 0.36 | | 1.43 | 0.36 |
| | 0.22 | -1.49 | | 1.68 | 0.52 | | 3.19 | 1.16 | | 2.86 | 1.05 | | 0.81 | -0.21 | | 2.37 | 0.86 | | 1.27 | 0.24 | | 0.91 | -0.09 | | 0.07 | -2.60 | | 0.33 | -1.11 | | 1.45 | 0.37 | | 1.33 | 0.28 |
| | 0.21 | -1.54 | | 1.80 | 0.59 | | 2.95 | 1.08 | | 2.79 | 1.03 | | 0.78 | -0.24 | | 2.27 | 0.82 | | 1.24 | 0.22 | | 0.84 | -0.18 | | 0.07 | -2.66 | | 0.38 | -0.98 | | 1.38 | 0.32 | | 1.23 | 0.21 |
| | 0.19 | -1.67 | | 1.57 | 0.45 | | 2.66 | 0.98 | | 2.67 | 0.98 | | 0.76 | -0.28 | | 2.23 | 0.80 | | 1.24 | 0.22 | | 0.81 | -0.21 | | 0.07 | -2.73 | | 0.23 | -1.48 | | 1.28 | 0.24 | | 1.12 | 0.11 |
| | 0.20 | -1.63 | | 1.32 | 0.28 | | 2.50 | 0.92 | | 2.51 | 0.92 | | 0.75 | -0.29 | | 2.19 | 0.79 | | 1.20 | 0.18 | | 0.74 | -0.30 | | 0.07 | -2.72 | | 0.25 | -1.38 | | 1.25 | 0.23 | | 1.27 | 0.24 |
| | 0.18 | -1.70 | | 1.23 | 0.21 | | 2.21 | 0.79 | | 2.34 | 0.85 | | 0.71 | -0.34 | | 2.18 | 0.78 | | 1.12 | 0.12 | | 0.70 | -0.36 | | 0.06 | -2.80 | | 0.31 | -1.19 | | 1.37 | 0.31 | | 1.18 | 0.17 |
| | 0.18 | -1.71 | | 0.94 | -0.06 | | 2.02 | 0.70 | | 2.36 | 0.86 | | 0.73 | -0.31 | | 2.19 | 0.78 | | 1.10 | 0.10 | | 0.59 | -0.52 | | 0.06 | -2.84 | | 0.33 | -1.10 | | 1.34 | 0.29 | | 1.18 | 0.17 |
| | 0.18 | -1.73 | | 1.07 | 0.06 | | 2.03 | 0.71 | | 2.21 | 0.79 | | 0.72 | -0.33 | | 2.05 | 0.72 | | 1.09 | 0.08 | | 0.58 | -0.55 | | 0.06 | -2.86 | | 0.34 | -1.07 | | 1.12 | 0.12 | | 1.06 | 0.06 |
| | 0.17 | -1.75 | | 1.05 | 0.05 | | 2.05 | 0.72 | | 2.21 | 0.79 | | 0.71 | -0.34 | | 2.07 | 0.73 | | 1.07 | 0.07 | | 0.57 | -0.56 | | 0.06 | -2.89 | | 0.35 | -1.05 | | 1.13 | 0.12 | | 0.94 | -0.06 |
| | 0.18 | -1.71 | | 1.05 | 0.05 | | 2.11 | 0.75 | | 2.20 | 0.79 | | 0.71 | -0.34 | | 2.09 | 0.74 | | 0.87 | -0.14 | | 0.58 | -0.55 | | 0.06 | -2.88 | | 0.36 | -1.03 | | 1.12 | 0.12 | | 0.87 | -0.14 |
| | 0.18 | -1.71 | | 1.03 | 0.03 | | 2.26 | 0.81 | | 2.18 | 0.78 | | 0.71 | -0.34 | | 2.11 | 0.75 | | 0.95 | -0.06 | | 0.56 | -0.58 | | 0.06 | -2.88 | | 0.36 | -1.01 | | 1.13 | 0.12 | | 0.85 | -0.16 |
| lower | val | upper | lower | val | upper | lower | val | upper | lower | val | upper | lower | val | upper | lower | val | upper | lower | val | upper | lower | val | upper | lower | val | upper | lower | val | upper | lower | val | upper | lower | val | upper |
| 0.29 | 0.06 | 0.07 | 0.06 | 2.19 | 2.33 | 2.06 | 0.57 | 0.60 | 0.53 | 0.46 | 0.49 | 0.44 | 0.18 | 0.19 | 0.17 | 1.17 | 1.41 | 0.98 | 0.13 | 0.14 | 0.12 | 0.29 | 0.31 | 0.26 | 0.03 | 0.03 | 0.03 | 0.77 | 0.83 | 0.70 | 1.50 | 1.60 | 1.42 | 1.34 | 1.41 |
| 0.29 | 0.08 | 0.08 | 0.08 | 1.73 | 1.86 | 1.62 | 0.52 | 0.55 | 0.49 | 0.48 | 0.50 | 0.45 | 0.15 | 0.16 | 0.15 | 1.15 | 1.37 | 0.96 | 0.13 | 0.15 | 0.12 | 0.16 | 0.18 | 0.15 | 0.03 | 0.03 | 0.03 | 0.94 | 1.02 | 0.87 | 1.58 | 1.68 | 1.50 | 0.94 | 1.00 |
| 0.25 | 0.08 | 0.08 | 0.07 | 1.60 | 1.73 | 1.49 | 0.41 | 0.44 | 0.39 | 0.48 | 0.50 | 0.46 | 0.16 | 0.17 | 0.15 | 1.06 | 1.28 | 0.88 | 0.13 | 0.14 | 0.12 | 0.24 | 0.26 | 0.22 | 0.03 | 0.03 | 0.03 | 0.66 | 0.71 | 0.61 | 1.11 | 1.19 | 1.04 | 0.74 | 0.79 |
| 0.18 | 0.08 | 0.08 | 0.08 | 1.28 | 1.40 | 1.17 | 0.39 | 0.41 | 0.36 | 0.44 | 0.46 | 0.42 | 0.14 | 0.15 | 0.13 | 1.08 | 1.29 | 0.89 | 0.11 | 0.13 | 0.10 | 0.32 | 0.35 | 0.30 | 0.03 | 0.03 | 0.03 | 1.71 | 1.83 | 1.59 | 0.96 | 1.03 | 0.90 | 0.71 | 0.76 |
| 0.23 | 0.08 | 0.08 | 0.08 | 0.73 | 0.80 | 0.66 | 0.46 | 0.49 | 0.44 | 0.41 | 0.42 | 0.39 | 0.14 | 0.15 | 0.13 | 1.05 | 1.27 | 0.87 | 0.11 | 0.12 | 0.10 | 0.23 | 0.25 | 0.21 | 0.03 | 0.03 | 0.03 | 2.46 | 2.63 | 2.30 | 0.71 | 0.76 | 0.66 | 0.69 | 0.73 |
| 0.20 | 0.07 | 0.07 | 0.07 | 0.72 | 0.80 | 0.65 | 0.29 | 0.31 | 0.27 | 0.36 | 0.38 | 0.35 | 0.12 | 0.13 | 0.11 | 1.00 | 1.20 | 0.83 | 0.09 | 0.10 | 0.09 | 0.25 | 0.27 | 0.23 | 0.02 | 0.02 | 0.02 | 1.02 | 1.10 | 0.95 | 0.77 | 0.82 | 0.72 | 0.63 | 0.67 |
| 0.23 | 0.07 | 0.07 | 0.07 | 0.74 | 0.81 | 0.67 | 0.35 | 0.37 | 0.33 | 0.36 | 0.38 | 0.35 | 0.10 | 0.11 | 0.10 | 0.95 | 1.14 | 0.78 | 0.09 | 0.10 | 0.08 | 0.29 | 0.32 | 0.27 | 0.02 | 0.02 | 0.02 | 0.66 | 0.72 | 0.61 | 0.80 | 0.85 | 0.74 | 0.58 | 0.63 |
| 0.17 | 0.05 | 0.06 | 0.05 | 0.54 | 0.61 | 0.48 | 0.37 | 0.39 | 0.35 | 0.35 | 0.37 | 0.33 | 0.10 | 0.10 | 0.09 | 0.87 | 1.03 | 0.70 | 0.08 | 0.09 | 0.07 | 0.31 | 0.34 | 0.28 | 0.02 | 0.02 | 0.02 | 0.51 | 0.56 | 0.47 | 0.58 | 0.62 | 0.54 | 0.57 | 0.61 |
| 0.17 | 0.06 | 0.06 | 0.06 | 0.45 | 0.50 | 0.40 | 0.43 | 0.46 | 0.40 | 0.37 | 0.38 | 0.35 | 0.09 | 0.10 | 0.09 | 0.84 | 1.01 | 0.69 | 0.09 | 0.10 | 0.08 | 0.33 | 0.35 | 0.30 | 0.02 | 0.02 | 0.02 | 0.49 | 0.53 | 0.45 | 0.61 | 0.65 | 0.57 | 0.51 | 0.54 |
| 0.19 | 0.05 | 0.05 | 0.05 | 0.64 | 0.71 | 0.58 | 0.35 | 0.38 | 0.33 | 0.37 | 0.38 | 0.35 | 0.10 | 0.11 | 0.10 | 0.81 | 0.98 | 0.66 | 0.09 | 0.10 | 0.08 | 0.39 | 0.43 | 0.36 | 0.02 | 0.02 | 0.02 | 0.36 | 0.39 | 0.33 | 0.32 | 0.34 | 0.30 | 0.52 | 0.55 |
| 0.18 | 0.05 | 0.05 | 0.05 | 0.48 | 0.54 | 0.43 | 0.27 | 0.29 | 0.25 | 0.34 | 0.36 | 0.32 | 0.08 | 0.09 | 0.08 | 0.77 | 0.93 | 0.62 | 0.09 | 0.10 | 0.08 | 0.30 | 0.33 | 0.28 | 0.02 | 0.02 | 0.02 | 0.25 | 0.28 | 0.23 | 0.36 | 0.39 | 0.33 | 0.41 | 0.44 |
| 0.18 | 0.05 | 0.05 | 0.05 | 0.55 | 0.61 | 0.50 | 0.30 | 0.32 | 0.28 | 0.37 | 0.39 | 0.35 | 0.08 | 0.09 | 0.08 | 0.72 | 0.88 | 0.57 | 0.09 | 0.09 | 0.08 | 0.23 | 0.25 | 0.21 | 0.02 | 0.02 | 0.02 | 0.30 | 0.33 | 0.27 | 0.43 | 0.46 | 0.40 | 0.38 | 0.40 |
| 0.09 | 0.04 | 0.04 | 0.03 | 0.32 | 0.36 | 0.30 | 0.26 | 0.28 | 0.24 | 0.38 | 0.40 | 0.36 | 0.08 | 0.09 | 0.08 | 0.69 | 0.84 | 0.55 | 0.09 | 0.10 | 0.08 | 0.24 | 0.27 | 0.22 | 0.02 | 0.02 | 0.02 | 0.14 | 0.15 | 0.13 | 0.34 | 0.37 | 0.32 | 0.27 | 0.29 |
| 0.11 | 0.04 | 0.04 | 0.04 | 0.35 | 0.38 | 0.32 | 0.27 | 0.29 | 0.25 | 0.34 | 0.36 | 0.33 | 0.08 | 0.08 | 0.07 | 0.67 | 0.81 | 0.52 | 0.08 | 0.09 | 0.07 | 0.22 | 0.24 | 0.20 | 0.02 | 0.02 | 0.02 | 0.17 | 0.18 | 0.15 | 0.32 | 0.34 | 0.29 | 0.38 | 0.41 |
| 0.13 | 0.03 | 0.04 | 0.03 | 0.27 | 0.29 | 0.24 | 0.23 | 0.24 | 0.21 | 0.31 | 0.33 | 0.30 | 0.07 | 0.08 | 0.07 | 0.64 | 0.79 | 0.49 | 0.08 | 0.08 | 0.07 | 0.19 | 0.21 | 0.18 | 0.02 | 0.02 | 0.02 | 0.22 | 0.24 | 0.20 | 0.49 | 0.53 | 0.45 | 0.36 | 0.39 |
| 0.16 | 0.03 | 0.03 | 0.03 | 0.10 | 0.11 | 0.09 | 0.21 | 0.22 | 0.19 | 0.31 | 0.33 | 0.29 | 0.08 | 0.08 | 0.07 | 0.63 | 0.79 | 0.49 | 0.07 | 0.08 | 0.06 | 0.16 | 0.18 | 0.15 | 0.02 | 0.02 | 0.02 | 0.25 | 0.28 | 0.22 | 0.48 | 0.52 | 0.44 | 0.38 | 0.41 |
| 0.13 | 0.03 | 0.03 | 0.03 | 0.28 | 0.30 | 0.25 | 0.19 | 0.20 | 0.17 | 0.30 | 0.33 | 0.28 | 0.08 | 0.09 | 0.08 | 0.51 | 0.64 | 0.38 | 0.07 | 0.08 | 0.06 | 0.17 | 0.19 | 0.15 | 0.02 | 0.02 | 0.02 | 0.26 | 0.30 | 0.23 | 0.40 | 0.44 | 0.36 | 0.28 | 0.30 |
| 0.13 | 0.03 | 0.03 | 0.03 | 0.25 | 0.30 | 0.21 | 0.21 | 0.23 | 0.19 | 0.31 | 0.34 | 0.29 | 0.08 | 0.09 | 0.08 | 0.54 | 0.70 | 0.40 | 0.06 | 0.07 | 0.05 | 0.18 | 0.20 | 0.16 | 0.02 | 0.02 | 0.02 | 0.27 | 0.31 | 0.22 | 0.40 | 0.47 | 0.34 | 0.31 | 0.33 |
| 0.13 | 0.03 | 0.03 | 0.03 | 0.26 | 0.32 | 0.21 | 0.21 | 0.23 | 0.19 | 0.31 | 0.34 | 0.29 | 0.08 | 0.09 | 0.08 | 0.56 | 0.74 | 0.41 | 0.04 | 0.04 | 0.03 | 0.18 | 0.20 | 0.16 | 0.02 | 0.02 | 0.02 | 0.27 | 0.33 | 0.22 | 0.41 | 0.50 | 0.33 | 0.29 | 0.34 |
| 0.13 | 0.04 | 0.04 | 0.03 | 0.27 | 0.34 | 0.20 | 0.21 | 0.24 | 0.20 | 0.32 | 0.35 | 0.29 | 0.08 | 0.09 | 0.08 | 0.58 | 0.78 | 0.42 | 0.06 | 0.06 | 0.05 | 0.18 | 0.20 | 0.16 | 0.02 | 0.02 | 0.02 | 0.28 | 0.35 | 0.22 | 0.42 | 0.53 | 0.34 | 0.30 | 0.36 |
| 1.16 | 0.23 | 0.36 | 0.21 | 2.00 | 2.95 | 1.73 | 4.49 | 6.91 | 3.98 | 3.58 | 4.19 | 2.91 | 0.94 | 1.17 | 0.78 | 1.80 | 2.43 | 0.99 | 1.68 | 2.03 | 1.30 | 1.00 | 2.15 | 0.78 | 0.06 | 0.10 | 0.06 | 0.09 | 0.12 | 0.07 | 1.44 | 2.12 | 1.24 | 1.06 | 1.64 |
| 1.06 | 0.23 | 0.35 | 0.21 | 2.08 | 2.97 | 1.79 | 4.22 | 6.39 | 3.75 | 3.42 | 3.96 | 2.77 | 0.90 | 1.11 | 0.74 | 1.64 | 2.36 | 0.95 | 1.56 | 1.83 | 1.19 | 0.96 | 2.04 | 0.75 | 0.06 | 0.09 | 0.05 | 0.07 | 0.10 | 0.06 | 1.52 | 2.17 | 1.30 | 1.09 | 1.66 |
| 1.04 | 0.22 | 0.32 | 0.20 | 1.95 | 2.62 | 1.65 | 3.81 | 5.66 | 3.40 | 3.20 | 3.70 | 2.59 | 0.89 | 1.08 | 0.72 | 1.52 | 2.34 | 0.96 | 1.43 | 1.67 | 1.08 | 0.90 | 1.92 | 0.71 | 0.06 | 0.09 | 0.05 | 0.08 | 0.10 | 0.06 | 1.44 | 1.96 | 1.23 | 1.00 | 1.50 |
| 0.97 | 0.20 | 0.30 | 0.19 | 1.73 | 2.24 | 1.41 | 3.63 | 5.29 | 3.26 | 3.03 | 3.50 | 2.44 | 0.90 | 1.08 | 0.72 | 1.53 | 2.32 | 0.97 | 1.37 | 1.59 | 1.01 | 0.75 | 1.61 | 0.59 | 0.05 | 0.09 | 0.05 | 0.07 | 0.09 | 0.06 | 1.21 | 1.57 | 0.99 | 0.94 | 1.37 |
| 0.93 | 0.18 | 0.27 | 0.17 | 1.74 | 2.17 | 1.35 | 3.41 | 4.90 | 3.07 | 2.69 | 3.11 | 2.15 | 0.84 | 1.01 | 0.67 | 1.43 | 2.40 | 0.97 | 1.33 | 1.55 | 0.98 | 0.74 | 1.58 | 0.58 | 0.05 | 0.08 | 0.05 | 0.07 | 0.08 | 0.05 | 1.14 | 1.42 | 0.89 | 0.91 | 1.29 |
| 0.82 | 0.18 | 0.26 | 0.16 | 1.58 | 1.87 | 1.17 | 3.14 | 4.49 | 2.84 | 2.61 | 3.00 | 2.08 | 0.80 | 0.94 | 0.62 | 1.45 | 2.44 | 0.98 | 1.29 | 1.50 | 0.95 | 0.68 | 1.44 | 0.53 | 0.05 | 0.08 | 0.05 | 0.07 | 0.09 | 0.06 | 1.23 | 1.48 | 0.94 | 0.99 | 1.37 |
| 0.82 | 0.18 | 0.27 | 0.17 | 1.53 | 1.77 | 1.09 | 3.12 | 4.47 | 2.81 | 2.47 | 2.86 | 1.97 | 0.75 | 0.89 | 0.58 | 1.47 | 2.44 | 0.99 | 1.29 | 1.50 | 0.96 | 0.66 | 1.39 | 0.52 | 0.05 | 0.07 | 0.04 | 0.07 | 0.09 | 0.06 | 1.32 | 1.55 | 0.98 | 0.98 | 1.32 |
| 0.77 | 0.17 | 0.26 | 0.16 | 1.65 | 1.93 | 1.15 | 3.17 | 4.47 | 2.86 | 2.39 | 2.82 | 1.89 | 0.73 | 0.84 | 0.55 | 1.47 | 2.49 | 0.99 | 1.26 | 1.47 | 0.94 | 0.62 | 1.32 | 0.49 | 0.04 | 0.07 | 0.04 | 0.07 | 0.08 | 0.05 | 1.30 | 1.52 | 0.94 | 1.03 | 1.35 |
| 0.73 | 0.17 | 0.25 | 0.16 | 1.36 | 1.56 | 0.91 | 3.11 | 4.39 | 2.80 | 2.38 | 2.81 | 1.87 | 0.71 | 0.83 | 0.54 | 1.50 | 2.48 | 0.99 | 1.22 | 1.43 | 0.91 | 0.62 | 1.33 | 0.49 | 0.04 | 0.07 | 0.04 | 0.08 | 0.10 | 0.06 | 1.11 | 1.30 | 0.80 | 0.92 | 1.22 |
| 0.73 | 0.16 | 0.24 | 0.15 | 1.26 | 1.45 | 0.83 | 3.00 | 4.19 | 2.70 | 2.39 | 2.83 | 1.88 | 0.70 | 0.81 | 0.53 | 1.53 | 2.46 | 1.00 | 1.20 | 1.41 | 0.90 | 0.66 | 1.42 | 0.52 | 0.04 | 0.06 | 0.04 | 0.07 | 0.09 | 0.06 | 1.00 | 1.17 | 0.71 | 0.75 | 0.97 |
| 0.69 | 0.15 | 0.23 | 0.14 | 1.10 | 1.30 | 0.68 | 2.88 | 3.95 | 2.58 | 2.32 | 2.77 | 1.81 | 0.69 | 0.80 | 0.51 | 1.49 | 2.42 | 0.98 | 1.16 | 1.37 | 0.87 | 0.56 | 1.21 | 0.44 | 0.04 | 0.06 | 0.03 | 0.06 | 0.08 | 0.05 | 0.97 | 1.16 | 0.70 | 0.78 | 0.99 |
| 0.63 | 0.14 | 0.22 | 0.12 | 1.15 | 1.35 | 0.69 | 2.61 | 3.54 | 2.35 | 2.23 | 2.65 | 1.72 | 0.66 | 0.77 | 0.49 | 1.45 | 2.35 | 0.93 | 1.13 | 1.34 | 0.85 | 0.56 | 1.21 | 0.44 | 0.04 | 0.06 | 0.03 | 0.06 | 0.07 | 0.05 | 0.85 | 1.01 | 0.59 | 0.73 | 0.91 |
| 0.57 | 0.13 | 0.20 | 0.12 | 1.16 | 1.35 | 0.68 | 2.36 | 3.18 | 2.11 | 2.11 | 2.51 | 1.62 | 0.64 | 0.74 | 0.47 | 1.44 | 2.34 | 0.91 | 1.13 | 1.34 | 0.85 | 0.51 | 1.11 | 0.40 | 0.03 | 0.05 | 0.03 | 0.07 | 0.09 | 0.06 | 0.84 | 1.00 | 0.56 | 0.74 | 0.91 |
| 0.55 | 0.13 | 0.20 | 0.12 | 0.90 | 1.10 | 0.52 | 2.20 | 2.95 | 1.97 | 1.99 | 2.38 | 1.52 | 0.64 | 0.75 | 0.47 | 1.45 | 2.31 | 0.90 | 1.10 | 1.34 | 0.84 | 0.47 | 1.02 | 0.37 | 0.03 | 0.05 | 0.03 | 0.07 | 0.09 | 0.06 | 0.84 | 0.99 | 0.55 | 0.77 | 0.95 |
| 0.53 | 0.13 | 0.19 | 0.11 | 0.90 | 1.07 | 0.50 | 1.95 | 2.64 | 1.71 | 1.85 | 2.20 | 1.39 | 0.61 | 0.72 | 0.45 | 1.46 | 2.28 | 0.90 | 1.02 | 1.25 | 0.77 | 0.45 | 0.96 | 0.35 | 0.03 | 0.05 | 0.03 | 0.07 | 0.10 | 0.06 | 0.79 | 0.93 | 0.50 | 0.71 | 0.88 |
| 0.47 | 0.13 | 0.19 | 0.11 | 0.78 | 0.95 | 0.43 | 1.77 | 2.41 | 1.54 | 1.88 | 2.23 | 1.42 | 0.62 | 0.74 | 0.46 | 1.49 | 2.24 | 0.88 | 1.01 | 1.23 | 0.75 | 0.39 | 0.83 | 0.30 | 0.03 | 0.04 | 0.03 | 0.07 | 0.09 | 0.06 | 0.78 | 0.93 | 0.49 | 0.71 | 0.86 |
| 0.49 | 0.13 | 0.19 | 0.11 | 0.73 | 0.91 | 0.40 | 1.80 | 2.44 | 1.57 | 1.75 | 2.10 | 1.31 | 0.61 | 0.72 | 0.46 | 1.47 | 2.16 | 0.85 | 1.00 | 1.23 | 0.74 | 0.36 | 0.78 | 0.27 | 0.03 | 0.04 | 0.02 | 0.07 | 0.09 | 0.06 | 0.65 | 0.84 | 0.42 | 0.69 | 0.83 |
| 0.50 | 0.12 | 0.19 | 0.11 | 0.74 | 0.97 | 0.40 | 1.80 | 2.45 | 1.56 | 1.75 | 2.12 | 1.32 | 0.60 | 0.72 | 0.45 | 1.47 | 2.17 | 0.84 | 0.98 | 1.23 | 0.73 | 0.35 | 0.76 | 0.26 | 0.03 | 0.04 | 0.02 | 0.07 | 0.09 | 0.06 | 0.64 | 0.85 | 0.41 | 0.55 | 0.70 |
| 0.50 | 0.13 | 0.19 | 0.11 | 0.73 | 0.99 | 0.39 | 1.86 | 2.54 | 1.60 | 1.73 | 2.09 | 1.30 | 0.60 | 0.72 | 0.45 | 1.46 | 2.18 | 0.85 | 0.82 | 1.02 | 0.59 | 0.35 | 0.76 | 0.26 | 0.03 | 0.04 | 0.02 | 0.07 | 0.09 | 0.05 | 0.64 | 0.87 | 0.40 | 0.50 | 0.67 |
| 0.47 | 0.12 | 0.19 | 0.11 | 0.71 | 1.00 | 0.40 | 2.01 | 2.73 | 1.71 | 1.70 | 2.07 | 1.28 | 0.60 | 0.73 | 0.44 | 1.46 | 2.15 | 0.85 | 0.87 | 1.08 | 0.64 | 0.34 | 0.73 | 0.25 | 0.03 | 0.04 | 0.02 | 0.07 | 0.10 | 0.06 | 0.63 | 0.89 | 0.40 | 0.47 | 0.66 |
| 0.04 | 0.03 | 0.04 | 0.03 | 0.17 | 0.24 | 0.13 | 0.07 | 0.09 | 0.04 | 0.25 | 0.27 | 0.18 | 0.06 | 0.07 | 0.05 | 0.35 | 0.47 | 0.13 | 0.04 | 0.05 | 0.03 | 0.06 | 0.09 | 0.05 | 0.02 | 0.03 | 0.02 | 0.03 | 0.03 | 0.02 | 0.17 | 0.24 | 0.13 | 0.23 | 0.27 |
| 0.04 | 0.03 | 0.04 | 0.03 | 0.14 | 0.24 | 0.11 | 0.07 | 0.09 | 0.04 | 0.24 | 0.27 | 0.18 | 0.06 | 0.07 | 0.04 | 0.30 | 0.40 | 0.12 | 0.04 | 0.05 | 0.03 | 0.05 | 0.08 | 0.04 | 0.02 | 0.03 | 0.02 | 0.02 | 0.03 | 0.02 | 0.17 | 0.24 | 0.13 | 0.22 | 0.27 |
| 0.04 | 0.03 | 0.04 | 0.03 | 0.15 | 0.23 | 0.12 | 0.07 | 0.09 | 0.04 | 0.24 | 0.27 | 0.17 | 0.05 | 0.06 | 0.04 | 0.26 | 0.34 | 0.12 | 0.03 | 0.05 | 0.03 | 0.05 | 0.08 | 0.04 | 0.02 | 0.03 | 0.02 | 0.02 | 0.03 | 0.02 | 0.16 | 0.22 | 0.13 | 0.21 | 0.26 |
| 0.04 | 0.03 | 0.04 | 0.03 | 0.14 | 0.20 | 0.11 | 0.07 | 0.08 | 0.04 | 0.24 | 0.26 | 0.16 | 0.05 | 0.06 | 0.04 | 0.23 | 0.30 | 0.12 | 0.03 | 0.04 | 0.03 | 0.05 | 0.07 | 0.04 | 0.02 | 0.03 | 0.02 | 0.02 | 0.03 | 0.02 | 0.14 | 0.20 | 0.11 | 0.20 | 0.25 |
| 0.04 | 0.03 | 0.04 | 0.02 | 0.13 | 0.19 | 0.11 | 0.06 | 0.07 | 0.04 | 0.22 | 0.24 | 0.15 | 0.05 | 0.06 | 0.04 | 0.19 | 0.25 | 0.12 | 0.03 | 0.04 | 0.02 | 0.05 | 0.07 | 0.04 | 0.02 | 0.03 | 0.02 | 0.02 | 0.03 | 0.02 | 0.14 | 0.19 | 0.11 | 0.20 | 0.25 |
| 0.04 | 0.03 | 0.04 | 0.02 | 0.12 | 0.17 | 0.10 | 0.06 | 0.07 | 0.04 | 0.22 | 0.24 | 0.15 | 0.04 | 0.05 | 0.04 | 0.18 | 0.24 | 0.11 | 0.03 | 0.04 | 0.02 | 0.05 | 0.07 | 0.04 | 0.02 | 0.03 | 0.02 | 0.02 | 0.03 | 0.02 | 0.15 | 0.19 | 0.11 | 0.20 | 0.25 |
| 0.04 | 0.03 | 0.04 | 0.02 | 0.12 | 0.17 | 0.09 | 0.06 | 0.07 | 0.04 | 0.22 | 0.24 | 0.14 | 0.04 | 0.05 | 0.03 | 0.16 | 0.23 | 0.11 | 0.03 | 0.04 | 0.02 | 0.05 | 0.06 | 0.04 | 0.02 | 0.02 | 0.02 | 0.02 | 0.03 | 0.02 | 0.13 | 0.18 | 0.11 | 0.19 | 0.25 |
| 0.04 | 0.03 | 0.04 | 0.02 | 0.11 | 0.16 | 0.09 | 0.05 | 0.06 | 0.04 | 0.21 | 0.23 | 0.13 | 0.04 | 0.05 | 0.03 | 0.14 | 0.23 | 0.10 | 0.03 | 0.04 | 0.02 | 0.05 | 0.06 | 0.04 | 0.02 | 0.02 | 0.02 | 0.02 | 0.02 | 0.02 | 0.15 | 0.18 | 0.10 | 0.19 | 0.25 |
| 0.03 | 0.03 | 0.03 | 0.02 | 0.11 | 0.14 | 0.08 | 0.04 | 0.06 | 0.03 | 0.20 | 0.22 | 0.13 | 0.04 | 0.05 | 0.03 | 0.13 | 0.22 | 0.10 | 0.03 | 0.04 | 0.02 | 0.05 | 0.06 | 0.04 | 0.02 | 0.02 | 0.02 | 0.02 | 0.03 | 0.02 | 0.13 | 0.16 | 0.09 | 0.18 | 0.22 |
| 0.03 | 0.03 | 0.03 | 0.02 | 0.11 | 0.14 | 0.08 | 0.04 | 0.06 | 0.03 | 0.20 | 0.22 | 0.13 | 0.04 | 0.05 | 0.03 | 0.12 | 0.21 | 0.09 | 0.03 | 0.04 | 0.02 | 0.06 | 0.07 | 0.04 | 0.02 | 0.02 | 0.02 | 0.02 | 0.02 | 0.02 | 0.12 | 0.15 | 0.08 | 0.16 | 0.19 |
| 0.02 | 0.02 | 0.03 | 0.02 | 0.10 | 0.13 | 0.07 | 0.04 | 0.05 | 0.03 | 0.19 | 0.21 | 0.12 | 0.04 | 0.05 | 0.03 | 0.11 | 0.21 | 0.08 | 0.03 | 0.04 | 0.02 | 0.05 | 0.06 | 0.04 | 0.02 | 0.02 | 0.01 | 0.02 | 0.02 | 0.02 | 0.11 | 0.14 | 0.08 | 0.14 | 0.17 |
| 0.02 | 0.02 | 0.03 | 0.02 | 0.10 | 0.13 | 0.07 | 0.04 | 0.05 | 0.03 | 0.19 | 0.21 | 0.12 | 0.04 | 0.05 | 0.03 | 0.10 | 0.21 | 0.06 | 0.03 | 0.03 | 0.02 | 0.05 | 0.06 | 0.03 | 0.02 | 0.02 | 0.01 | 0.02 | 0.02 | 0.01 | 0.10 | 0.13 | 0.07 | 0.13 | 0.16 |
| 0.02 | 0.02 | 0.03 | 0.02 | 0.09 | 0.12 | 0.06 | 0.04 | 0.05 | 0.03 | 0.18 | 0.20 | 0.11 | 0.03 | 0.04 | 0.03 | 0.09 | 0.21 | 0.06 | 0.03 | 0.03 | 0.02 | 0.06 | 0.06 | 0.03 | 0.02 | 0.02 | 0.01 | 0.02 | 0.02 | 0.01 | 0.09 | 0.12 | 0.07 | 0.12 | 0.15 |
| 0.02 | 0.02 | 0.03 | 0.02 | 0.07 | 0.10 | 0.06 | 0.04 | 0.05 | 0.03 | 0.17 | 0.19 | 0.11 | 0.03 | 0.04 | 0.03 | 0.08 | 0.21 | 0.05 | 0.03 | 0.03 | 0.02 | 0.05 | 0.06 | 0.03 | 0.01 | 0.02 | 0.01 | 0.02 | 0.02 | 0.01 | 0.09 | 0.12 | 0.07 | 0.12 | 0.15 |
| 0.02 | 0.02 | 0.03 | 0.02 | 0.07 | 0.10 | 0.05 | 0.04 | 0.05 | 0.03 | 0.17 | 0.19 | 0.11 | 0.03 | 0.04 | 0.03 | 0.07 | 0.22 | 0.04 | 0.02 | 0.03 | 0.02 | 0.05 | 0.06 | 0.03 | 0.01 | 0.02 | 0.01 | 0.01 | 0.02 | 0.01 | 0.09 | 0.11 | 0.06 | 0.11 | 0.15 |
| 0.02 | 0.02 | 0.03 | 0.02 | 0.06 | 0.09 | 0.05 | 0.04 | 0.04 | 0.03 | 0.17 | 0.19 | 0.10 | 0.03 | 0.04 | 0.03 | 0.07 | 0.22 | 0.03 | 0.02 | 0.03 | 0.02 | 0.05 | 0.06 | 0.03 | 0.01 | 0.02 | 0.01 | 0.01 | 0.02 | 0.01 | 0.08 | 0.11 | 0.06 | 0.10 | 0.14 |
| 0.02 | 0.02 | 0.03 | 0.02 | 0.06 | 0.09 | 0.04 | 0.04 | 0.05 | 0.03 | 0.16 | 0.18 | 0.10 | 0.03 | 0.04 | 0.03 | 0.07 | 0.22 | 0.03 | 0.02 | 0.03 | 0.02 | 0.04 | 0.05 | 0.03 | 0.01 | 0.02 | 0.01 | 0.01 | 0.02 | 0.01 | 0.08 | 0.11 | 0.06 | 0.10 | 0.13 |
| 0.02 | 0.02 | 0.03 | 0.02 | 0.06 | 0.09 | 0.04 | 0.04 | 0.04 | 0.03 | 0.16 | 0.18 | 0.10 | 0.03 | 0.04 | 0.03 | 0.07 | 0.21 | 0.04 | 0.02 | 0.03 | 0.02 | 0.04 | 0.05 | 0.03 | 0.01 | 0.02 | 0.01 | 0.01 | 0.02 | 0.01 | 0.08 | 0.11 | 0.05 | 0.08 | 0.11 |
| 0.02 | 0.02 | 0.03 | 0.02 | 0.06 | 0.09 | 0.04 | 0.04 | 0.04 | 0.03 | 0.16 | 0.18 | 0.10 | 0.03 | 0.04 | 0.03 | 0.07 | 0.22 | 0.04 | 0.02 | 0.03 | 0.02 | 0.04 | 0.05 | 0.03 | 0.01 | 0.02 | 0.01 | 0.01 | 0.02 | 0.01 | 0.08 | 0.11 | 0.06 | 0.08 | 0.11 |
| 0.02 | 0.02 | 0.03 | 0.02 | 0.06 | 0.09 | 0.04 | 0.04 | 0.04 | 0.03 | 0.15 | 0.18 | 0.10 | 0.03 | 0.04 | 0.03 | 0.07 | 0.21 | 0.04 | 0.02 | 0.03 | 0.02 | 0.04 | 0.05 | 0.02 | 0.01 | 0.02 | 0.01 | 0.01 | 0.02 | 0.01 | 0.08 | 0.12 | 0.05 | 0.08 | 0.11 |

| | | | | | | | | | | | | | | | | | | | | | | | | | | | | | | | | | | | |
|---|---|---|---|---|---|---|---|---|---|---|---|---|---|---|---|---|---|---|---|---|---|---|---|---|---|---|---|---|---|---|---|---|---|---|---|
| nia | Luxembourg | | | Monaco | | | Netherlands | | | New Zealand | | | Northern Ireland | | | Norway | | | Puerto Rico | | | Qatar | | | South Korea | | | Russia | | | San Marino | | | Saudi A | |
| | Luxem | AAPC | | Monac | AAPC | | Nether | AAPC | | New Z | AAPC | | Northe | AAPC | | Norwa | AAPC | | Puertc | AAPC | | Qatar | AAPC | | South | AAPC | | Russia | AAPC | | San M | AAPC | | Saudi A | AAPC |
| ##### | | -3.8 | ##### | | -0.7 | ##### | | -3.5 | ##### | | -2.9 | ##### | | -2.7 | ##### | | -4.5 | ##### | | 0.7 | -100 | | -3.3 | ##### | | -3.9 | ##### | | -6.8 | ##### | | -0.1 | 840 | | -2.5 |
| ##### | -2.1 | **-1.9** | ##### | -2.86 | **-1.4** | ##### | -0.89 | **-1.9** | ##### | -1.02 | **-1.1** | ##### | -1.24 | **-3.7** | ##### | -5.79 | **-4.1** | ##### | -53.2 | **-3.2** | ##### | -0.84 | **-3.0** | ##### | -0.24 | **-3.1** | ##### | -10.2 | **-5.6** | ##### | 1.51 | **0.5** | -100 | -4.56 | **-3.2** |
| | | Ln | | | Ln | | | Ln | | | Ln | | | Ln | | | Ln | | | Ln | | | Ln | | | Ln | | | Ln | | | Ln | | | Ln |
| | 2.03 | 0.71 | | 2.28 | 0.82 | | 0.82 | -0.20 | | 1.53 | 0.43 | | 0.45 | -0.80 | | 2.93 | 1.08 | | 18.1 | 2.90 | | 0.43 | -0.84 | | 0.15 | -1.90 | | 4.52 | 1.51 | | 3.18 | 1.16 | | 2.04 | 0.71 |
| | 1.93 | 0.66 | | 2.26 | 0.82 | | 0.81 | -0.21 | | 1.52 | 0.42 | | 0.42 | -0.87 | | 2.61 | 0.96 | | 20.1 | 3.00 | | 0.43 | -0.84 | | 0.13 | -2.04 | | 4.73 | 1.55 | | 3.09 | 1.13 | | 2.04 | 0.71 |
| | 1.91 | 0.65 | | 2.25 | 0.81 | | 0.77 | -0.26 | | 1.46 | 0.38 | | 0.43 | -0.84 | | 2.56 | 0.94 | | 19.3 | 2.96 | | 0.41 | -0.89 | | 0.13 | -2.04 | | 4.51 | 1.51 | | 3.00 | 1.10 | | 2.02 | 0.70 |
| | 1.82 | 0.60 | | 2.24 | 0.81 | | 0.74 | -0.30 | | 1.36 | 0.31 | | 0.38 | -0.97 | | 2.41 | 0.88 | | 19.3 | 2.96 | | 0.39 | -0.94 | | 0.13 | -2.04 | | 4.30 | 1.46 | | 2.90 | 1.06 | | 1.96 | 0.67 |
| | 1.72 | 0.54 | | 2.24 | 0.81 | | 0.70 | -0.36 | | 1.27 | 0.24 | | 0.41 | -0.89 | | 2.24 | 0.81 | | 19.2 | 2.95 | | 0.37 | -0.99 | | 0.11 | -2.21 | | 4.13 | 1.42 | | 2.89 | 1.06 | | 1.88 | 0.63 |
| | 1.61 | 0.48 | | 2.23 | 0.80 | | 0.66 | -0.42 | | 1.29 | 0.25 | | 0.42 | -0.87 | | 2.00 | 0.69 | | 20.5 | 3.02 | | 0.37 | -0.99 | | 0.10 | -2.30 | | 4.08 | 1.41 | | 2.86 | 1.05 | | 1.81 | 0.59 |
| | 1.52 | 0.42 | | 2.23 | 0.80 | | 0.55 | -0.60 | | 1.22 | 0.20 | | 0.45 | -0.80 | | 1.96 | 0.67 | | 20.5 | 3.02 | | 0.36 | -1.02 | | 0.10 | -2.30 | | 3.35 | 1.21 | | 2.86 | 1.05 | | 1.74 | 0.55 |
| | 1.37 | 0.31 | | 2.21 | 0.79 | | 0.56 | -0.58 | | 1.18 | 0.17 | | 0.42 | -0.87 | | 1.77 | 0.57 | | 20.9 | 3.04 | | 0.39 | -0.94 | | 0.10 | -2.30 | | 3.01 | 1.10 | | 2.86 | 1.05 | | 1.69 | 0.52 |
| | 1.30 | 0.26 | | 2.19 | 0.78 | | 0.55 | -0.60 | | 1.17 | 0.16 | | 0.42 | -0.87 | | 1.79 | 0.58 | | 22.5 | 3.11 | | 0.36 | -1.02 | | 0.10 | -2.30 | | 2.85 | 1.05 | | 2.84 | 1.04 | | 1.65 | 0.50 |
| | 1.25 | 0.23 | | 2.20 | 0.79 | | 0.52 | -0.66 | | 1.18 | 0.17 | | 0.39 | -0.94 | | 1.85 | 0.61 | | 25.3 | 3.23 | | 0.34 | -1.08 | | 0.09 | -2.36 | | 2.54 | 0.93 | | 2.85 | 1.05 | | 1.62 | 0.49 |
| | 1.19 | 0.18 | | 2.19 | 0.78 | | 0.47 | -0.75 | | 1.05 | 0.04 | | 0.40 | -0.92 | | 1.67 | 0.51 | | 25.6 | 3.24 | | 0.32 | -1.13 | | 0.09 | -2.41 | | 2.49 | 0.91 | | 2.86 | 1.05 | | 1.64 | 0.50 |
| | 1.19 | 0.18 | | 2.19 | 0.79 | | 0.53 | -0.64 | | 0.96 | -0.04 | | 0.38 | -0.98 | | 1.68 | 0.52 | | 27.1 | 3.30 | | 0.30 | -1.20 | | 0.09 | -2.45 | | 2.16 | 0.77 | | 2.88 | 1.06 | | 1.62 | 0.48 |
| | 1.18 | 0.17 | | 2.17 | 0.78 | | 0.53 | -0.64 | | 0.97 | -0.03 | | 0.33 | -1.10 | | 1.55 | 0.44 | | 25.6 | 3.24 | | 0.29 | -1.25 | | 0.08 | -2.50 | | 2.03 | 0.71 | | 2.90 | 1.06 | | 1.59 | 0.47 |
| | 1.12 | 0.11 | | 2.15 | 0.77 | | 0.45 | -0.79 | | 1.00 | 0.00 | | 0.32 | -1.14 | | 1.49 | 0.40 | | 24.7 | 3.21 | | 0.28 | -1.29 | | 0.08 | -2.54 | | 1.93 | 0.66 | | 2.92 | 1.07 | | 1.54 | 0.43 |
| | 1.12 | 0.11 | | 2.13 | 0.76 | | 0.47 | -0.75 | | 0.95 | -0.06 | | 0.29 | -1.24 | | 1.39 | 0.33 | | 21.5 | 3.07 | | 0.27 | -1.32 | | 0.08 | -2.59 | | 1.84 | 0.61 | | 2.94 | 1.08 | | 1.50 | 0.40 |
| | 1.11 | 0.11 | | 2.09 | 0.74 | | 0.44 | -0.83 | | 0.95 | -0.05 | | 0.30 | -1.21 | | 1.29 | 0.26 | | 20.3 | 3.01 | | 0.26 | -1.35 | | 0.07 | -2.63 | | 1.74 | 0.55 | | 2.96 | 1.09 | | 1.43 | 0.36 |
| | 1.05 | 0.05 | | 2.06 | 0.72 | | 0.42 | -0.87 | | 0.95 | -0.05 | | 0.27 | -1.30 | | 1.30 | 0.26 | | 20.9 | 3.04 | | 0.25 | -1.38 | | 0.07 | -2.66 | | 1.57 | 0.45 | | 2.98 | 1.09 | | 1.38 | 0.33 |
| | 1.05 | 0.05 | | 2.02 | 0.70 | | 0.44 | -0.82 | | 0.93 | -0.07 | | 0.28 | -1.27 | | 1.21 | 0.19 | | 20.5 | 3.02 | | 0.25 | -1.41 | | 0.07 | -2.67 | | 1.41 | 0.34 | | 2.99 | 1.10 | | 1.34 | 0.29 |
| | 1.04 | 0.04 | | 1.98 | 0.68 | | 0.44 | -0.82 | | 0.93 | -0.07 | | 0.29 | -1.24 | | 1.22 | 0.20 | | 20.8 | 3.04 | | 0.25 | -1.40 | | 0.07 | -2.68 | | 1.50 | 0.41 | | 2.99 | 1.09 | | 1.29 | 0.25 |
| | 1.02 | 0.02 | | 1.95 | 0.67 | | 0.44 | -0.82 | | 0.92 | -0.08 | | 0.28 | -1.26 | | 1.20 | 0.18 | | 20.8 | 3.04 | | 0.25 | -1.41 | | 0.07 | -2.68 | | 1.50 | 0.41 | | 2.97 | 1.09 | | 1.25 | 0.22 |
| lower | val | upper | lower | val | upper | lower | val | upper | lower | val | upper | lower | val | upper | lower | val | upper | lower | val | upper | lower | val | upper | lower | val | upper | lower | val | upper | lower | val | upper | lower | val | upper |
| 1.27 | 0.38 | 0.41 | 0.36 | 0.10 | 0.14 | 0.07 | 0.48 | 0.51 | 0.45 | 0.25 | 0.27 | 0.24 | 0.09 | 0.10 | 0.09 | 0.35 | 0.36 | 0.34 | 16.3 | 16.9 | 15.7 | 0.15 | 0.21 | 0.10 | 0.05 | 0.05 | 0.04 | 2.17 | 2.20 | 2.13 | 0.07 | 0.09 | 0.06 | 0.09 | 0.12 |
| 0.88 | 0.38 | 0.41 | 0.36 | 0.10 | 0.14 | 0.07 | 0.48 | 0.51 | 0.45 | 0.24 | 0.26 | 0.23 | 0.10 | 0.11 | 0.09 | 0.19 | 0.20 | 0.19 | 18.3 | 18.9 | 17.7 | 0.16 | 0.22 | 0.11 | 0.04 | 0.05 | 0.04 | 2.42 | 2.45 | 2.39 | 0.07 | 0.09 | 0.06 | 0.10 | 0.12 |
| 0.69 | 0.38 | 0.41 | 0.36 | 0.10 | 0.14 | 0.07 | 0.44 | 0.47 | 0.42 | 0.23 | 0.25 | 0.22 | 0.11 | 0.12 | 0.10 | 0.31 | 0.31 | 0.30 | 17.7 | 18.3 | 17.1 | 0.16 | 0.23 | 0.11 | 0.04 | 0.05 | 0.03 | 2.27 | 2.30 | 2.24 | 0.07 | 0.09 | 0.06 | 0.10 | 0.12 |
| 0.66 | 0.34 | 0.37 | 0.32 | 0.10 | 0.15 | 0.07 | 0.41 | 0.43 | 0.39 | 0.20 | 0.21 | 0.19 | 0.11 | 0.12 | 0.10 | 0.27 | 0.28 | 0.26 | 17.7 | 18.4 | 17.1 | 0.16 | 0.22 | 0.11 | 0.04 | 0.04 | 0.03 | 2.07 | 2.10 | 2.04 | 0.07 | 0.09 | 0.06 | 0.10 | 0.13 |
| 0.64 | 0.32 | 0.35 | 0.30 | 0.10 | 0.14 | 0.07 | 0.39 | 0.41 | 0.37 | 0.17 | 0.19 | 0.17 | 0.12 | 0.13 | 0.11 | 0.24 | 0.25 | 0.24 | 17.6 | 18.2 | 17.0 | 0.16 | 0.22 | 0.12 | 0.04 | 0.04 | 0.03 | 2.00 | 2.03 | 1.97 | 0.07 | 0.09 | 0.06 | 0.11 | 0.13 |
| 0.58 | 0.28 | 0.30 | 0.26 | 0.10 | 0.14 | 0.07 | 0.37 | 0.40 | 0.35 | 0.19 | 0.20 | 0.18 | 0.11 | 0.12 | 0.10 | 0.15 | 0.16 | 0.15 | 18.9 | 19.5 | 18.3 | 0.17 | 0.23 | 0.13 | 0.03 | 0.04 | 0.03 | 1.80 | 1.84 | 1.73 | 0.07 | 0.10 | 0.06 | 0.11 | 0.14 |
| 0.54 | 0.25 | 0.28 | 0.24 | 0.10 | 0.15 | 0.07 | 0.27 | 0.29 | 0.26 | 0.18 | 0.19 | 0.17 | 0.12 | 0.13 | 0.11 | 0.19 | 0.20 | 0.19 | 18.9 | 19.5 | 18.2 | 0.17 | 0.22 | 0.13 | 0.03 | 0.04 | 0.03 | 1.43 | 1.47 | 1.41 | 0.07 | 0.10 | 0.06 | 0.12 | 0.15 |
| 0.52 | 0.23 | 0.25 | 0.21 | 0.10 | 0.14 | 0.07 | 0.29 | 0.31 | 0.27 | 0.15 | 0.16 | 0.14 | 0.09 | 0.10 | 0.08 | 0.12 | 0.12 | 0.11 | 19.3 | 20.0 | 18.7 | 0.18 | 0.24 | 0.13 | 0.03 | 0.04 | 0.03 | 1.33 | 1.36 | 1.29 | 0.08 | 0.10 | 0.05 | 0.13 | 0.16 |
| 0.47 | 0.24 | 0.26 | 0.22 | 0.10 | 0.14 | 0.07 | 0.28 | 0.29 | 0.26 | 0.19 | 0.20 | 0.18 | 0.10 | 0.11 | 0.09 | 0.10 | 0.10 | 0.10 | 21.0 | 21.7 | 20.3 | 0.16 | 0.21 | 0.11 | 0.03 | 0.03 | 0.02 | 1.23 | 1.26 | 1.18 | 0.07 | 0.10 | 0.05 | 0.14 | 0.19 |
| 0.49 | 0.24 | 0.26 | 0.22 | 0.10 | 0.14 | 0.07 | 0.26 | 0.28 | 0.25 | 0.22 | 0.24 | 0.21 | 0.07 | 0.08 | 0.07 | 0.15 | 0.15 | 0.14 | 23.8 | 24.6 | 23.1 | 0.15 | 0.20 | 0.11 | 0.03 | 0.03 | 0.02 | 1.10 | 1.13 | 1.08 | 0.08 | 0.11 | 0.05 | 0.16 | 0.22 |
| 0.38 | 0.22 | 0.24 | 0.20 | 0.10 | 0.13 | 0.07 | 0.23 | 0.24 | 0.21 | 0.15 | 0.16 | 0.15 | 0.08 | 0.09 | 0.07 | 0.07 | 0.08 | 0.07 | 24.2 | 25.0 | 23.4 | 0.15 | 0.20 | 0.10 | 0.03 | 0.03 | 0.02 | 1.06 | 1.08 | 1.04 | 0.07 | 0.11 | 0.05 | 0.19 | 0.33 |
| 0.35 | 0.22 | 0.24 | 0.21 | 0.10 | 0.13 | 0.07 | 0.28 | 0.30 | 0.26 | 0.10 | 0.10 | 0.09 | 0.09 | 0.10 | 0.08 | 0.11 | 0.12 | 0.11 | 25.6 | 26.4 | 24.8 | 0.14 | 0.19 | 0.10 | 0.02 | 0.03 | 0.02 | 0.93 | 0.95 | 0.92 | 0.08 | 0.11 | 0.05 | 0.20 | 0.41 |
| 0.25 | 0.22 | 0.24 | 0.21 | 0.10 | 0.13 | 0.07 | 0.29 | 0.31 | 0.27 | 0.10 | 0.10 | 0.09 | 0.06 | 0.07 | 0.05 | 0.12 | 0.12 | 0.12 | 24.2 | 25.0 | 23.5 | 0.13 | 0.18 | 0.09 | 0.02 | 0.03 | 0.02 | 0.87 | 0.89 | 0.86 | 0.08 | 0.11 | 0.05 | 0.21 | 0.44 |
| 0.35 | 0.21 | 0.23 | 0.19 | 0.09 | 0.13 | 0.07 | 0.22 | 0.23 | 0.20 | 0.18 | 0.19 | 0.17 | 0.06 | 0.07 | 0.06 | 0.09 | 0.10 | 0.09 | 23.3 | 24.1 | 22.6 | 0.13 | 0.19 | 0.08 | 0.02 | 0.03 | 0.02 | 0.84 | 0.85 | 0.82 | 0.08 | 0.11 | 0.05 | 0.21 | 0.43 |
| 0.34 | 0.20 | 0.22 | 0.19 | 0.10 | 0.13 | 0.07 | 0.26 | 0.28 | 0.24 | 0.14 | 0.15 | 0.13 | 0.05 | 0.06 | 0.05 | 0.10 | 0.10 | 0.09 | 20.4 | 21.1 | 19.7 | 0.13 | 0.19 | 0.08 | 0.02 | 0.03 | 0.02 | 0.73 | 0.75 | 0.72 | 0.08 | 0.12 | 0.05 | 0.21 | 0.43 |
| 0.35 | 0.21 | 0.23 | 0.19 | 0.10 | 0.13 | 0.07 | 0.21 | 0.23 | 0.20 | 0.17 | 0.18 | 0.16 | 0.05 | 0.05 | 0.04 | 0.08 | 0.08 | 0.08 | 19.3 | 20.1 | 18.6 | 0.13 | 0.19 | 0.08 | 0.02 | 0.03 | 0.02 | 0.73 | 0.75 | 0.71 | 0.08 | 0.12 | 0.05 | 0.20 | 0.41 |
| 0.26 | 0.19 | 0.21 | 0.17 | 0.09 | 0.13 | 0.06 | 0.20 | 0.21 | 0.18 | 0.16 | 0.17 | 0.15 | 0.04 | 0.04 | 0.04 | 0.09 | 0.09 | 0.08 | 19.9 | 20.7 | 19.1 | 0.13 | 0.19 | 0.08 | 0.02 | 0.03 | 0.02 | 0.66 | 0.67 | 0.64 | 0.08 | 0.12 | 0.05 | 0.20 | 0.40 |
| 0.29 | 0.21 | 0.23 | 0.18 | 0.09 | 0.13 | 0.06 | 0.22 | 0.24 | 0.21 | 0.15 | 0.16 | 0.14 | 0.05 | 0.06 | 0.05 | 0.04 | 0.04 | 0.04 | 19.4 | 22.1 | 17.1 | 0.12 | 0.18 | 0.07 | 0.02 | 0.03 | 0.02 | 0.60 | 0.61 | 0.57 | 0.08 | 0.12 | 0.05 | 0.19 | 0.37 |
| 0.25 | 0.21 | 0.24 | 0.18 | 0.09 | 0.13 | 0.06 | 0.23 | 0.25 | 0.21 | 0.15 | 0.16 | 0.14 | 0.06 | 0.06 | 0.05 | 0.07 | 0.07 | 0.06 | 19.7 | 23.6 | 16.3 | 0.12 | 0.19 | 0.07 | 0.02 | 0.03 | 0.02 | 0.69 | 0.78 | 0.61 | 0.08 | 0.12 | 0.05 | 0.18 | 0.35 |
| 0.24 | 0.21 | 0.24 | 0.18 | 0.09 | 0.13 | 0.06 | 0.23 | 0.25 | 0.21 | 0.15 | 0.16 | 0.14 | 0.06 | 0.06 | 0.05 | 0.07 | 0.08 | 0.07 | 19.7 | 24.4 | 15.9 | 0.13 | 0.20 | 0.07 | 0.02 | 0.03 | 0.02 | 0.72 | 0.86 | 0.60 | 0.08 | 0.12 | 0.05 | 0.17 | 0.34 |
| 0.91 | 1.56 | 1.86 | 1.26 | 2.12 | 3.22 | 1.49 | 0.31 | 0.39 | 0.25 | 1.19 | 1.71 | 1.06 | 0.32 | 0.56 | 0.26 | 2.54 | 3.60 | 2.32 | 1.17 | 1.70 | 1.03 | 0.09 | 0.18 | 0.06 | 0.07 | 0.11 | 0.04 | 1.91 | 3.62 | 1.67 | 3.07 | 5.05 | 2.38 | 0.21 | 0.39 |
| 0.94 | 1.47 | 1.74 | 1.15 | 2.11 | 3.19 | 1.49 | 0.30 | 0.38 | 0.24 | 1.20 | 1.70 | 1.07 | 0.29 | 0.51 | 0.24 | 2.39 | 3.39 | 2.20 | 1.14 | 1.63 | 1.01 | 0.09 | 0.18 | 0.06 | 0.06 | 0.10 | 0.04 | 1.88 | 3.53 | 1.65 | 2.98 | 4.87 | 2.30 | 0.22 | 0.39 |
| 0.86 | 1.45 | 1.70 | 1.10 | 2.10 | 3.15 | 1.49 | 0.30 | 0.38 | 0.24 | 1.15 | 1.60 | 1.03 | 0.29 | 0.51 | 0.24 | 2.22 | 3.15 | 2.06 | 0.98 | 1.38 | 0.86 | 0.09 | 0.17 | 0.06 | 0.06 | 0.09 | 0.04 | 1.82 | 3.36 | 1.60 | 2.89 | 4.84 | 2.22 | 0.22 | 0.39 |
| 0.81 | 1.40 | 1.61 | 1.03 | 2.09 | 3.16 | 1.50 | 0.31 | 0.39 | 0.24 | 1.07 | 1.49 | 0.96 | 0.24 | 0.42 | 0.20 | 2.11 | 2.99 | 1.95 | 0.93 | 1.31 | 0.81 | 0.08 | 0.16 | 0.06 | 0.06 | 0.08 | 0.03 | 1.81 | 3.29 | 1.59 | 2.79 | 4.77 | 2.10 | 0.23 | 0.40 |
| 0.79 | 1.33 | 1.54 | 0.98 | 2.09 | 3.14 | 1.49 | 0.29 | 0.37 | 0.23 | 1.02 | 1.42 | 0.90 | 0.26 | 0.47 | 0.21 | 1.97 | 2.81 | 1.82 | 0.92 | 1.30 | 0.80 | 0.08 | 0.16 | 0.00 | 0.05 | 0.07 | 0.03 | 1.73 | 3.09 | 1.51 | 2.78 | 4.59 | 2.06 | 0.23 | 0.39 |
| 0.84 | 1.26 | 1.45 | 0.90 | 2.08 | 3.15 | 1.49 | 0.27 | 0.36 | 0.21 | 1.03 | 1.43 | 0.92 | 0.28 | 0.54 | 0.22 | 1.82 | 2.60 | 1.68 | 0.94 | 1.34 | 0.82 | 0.08 | 0.16 | 0.05 | 0.05 | 0.06 | 0.03 | 1.87 | 3.28 | 1.55 | 2.75 | 4.44 | 1.99 | 0.23 | 0.41 |
| 0.81 | 1.21 | 1.41 | 0.86 | 2.08 | 3.17 | 1.49 | 0.26 | 0.35 | 0.20 | 0.97 | 1.35 | 0.85 | 0.30 | 0.60 | 0.23 | 1.74 | 2.52 | 1.61 | 0.89 | 1.27 | 0.78 | 0.08 | 0.15 | 0.05 | 0.05 | 0.06 | 0.02 | 1.55 | 2.64 | 1.37 | 2.75 | 4.39 | 1.92 | 0.23 | 0.41 |
| 0.82 | 1.08 | 1.25 | 0.75 | 2.06 | 3.15 | 1.48 | 0.25 | 0.34 | 0.19 | 0.96 | 1.32 | 0.85 | 0.30 | 0.61 | 0.23 | 1.62 | 2.35 | 1.49 | 0.85 | 1.23 | 0.74 | 0.07 | 0.15 | 0.05 | 0.05 | 0.06 | 0.02 | 1.35 | 2.23 | 1.23 | 2.74 | 4.33 | 1.86 | 0.24 | 0.41 |
| 0.74 | 1.00 | 1.16 | 0.69 | 2.04 | 3.17 | 1.49 | 0.25 | 0.34 | 0.19 | 0.91 | 1.26 | 0.79 | 0.29 | 0.63 | 0.23 | 1.66 | 2.41 | 1.53 | 0.82 | 1.19 | 0.72 | 0.07 | 0.14 | 0.05 | 0.05 | 0.06 | 0.02 | 1.30 | 2.08 | 1.18 | 2.73 | 4.29 | 1.80 | 0.25 | 0.40 |
| 0.60 | 0.96 | 1.13 | 0.65 | 2.05 | 3.18 | 1.52 | 0.24 | 0.34 | 0.19 | 0.89 | 1.23 | 0.77 | 0.28 | 0.62 | 0.22 | 1.67 | 2.43 | 1.52 | 0.84 | 1.24 | 0.73 | 0.07 | 0.14 | 0.05 | 0.05 | 0.06 | 0.02 | 1.13 | 1.77 | 1.05 | 2.74 | 4.32 | 1.76 | 0.26 | 0.40 |
| 0.60 | 0.92 | 1.09 | 0.63 | 2.05 | 3.21 | 1.51 | 0.23 | 0.34 | 0.19 | 0.83 | 1.18 | 0.73 | 0.29 | 0.64 | 0.22 | 1.57 | 2.31 | 1.44 | 0.75 | 1.09 | 0.65 | 0.07 | 0.13 | 0.05 | 0.05 | 0.06 | 0.02 | 1.13 | 1.72 | 1.05 | 2.74 | 4.38 | 1.76 | 0.28 | 0.39 |
| 0.56 | 0.92 | 1.09 | 0.63 | 2.05 | 3.18 | 1.50 | 0.23 | 0.34 | 0.19 | 0.80 | 1.13 | 0.70 | 0.26 | 0.57 | 0.19 | 1.54 | 2.28 | 1.41 | 0.82 | 1.15 | 0.71 | 0.06 | 0.12 | 0.04 | 0.04 | 0.05 | 0.02 | 0.97 | 1.45 | 0.89 | 2.76 | 4.43 | 1.75 | 0.31 | 0.41 |
| 0.55 | 0.91 | 1.07 | 0.62 | 2.03 | 3.08 | 1.53 | 0.23 | 0.34 | 0.19 | 0.81 | 1.13 | 0.70 | 0.24 | 0.53 | 0.18 | 1.40 | 2.08 | 1.29 | 0.73 | 0.99 | 0.63 | 0.06 | 0.12 | 0.04 | 0.04 | 0.05 | 0.02 | 0.91 | 1.37 | 0.84 | 2.79 | 4.49 | 1.74 | 0.32 | 0.43 |
| 0.58 | 0.87 | 1.03 | 0.59 | 2.01 | 3.04 | 1.51 | 0.22 | 0.34 | 0.19 | 0.77 | 1.05 | 0.66 | 0.23 | 0.49 | 0.17 | 1.37 | 2.07 | 1.25 | 0.75 | 0.98 | 0.64 | 0.06 | 0.12 | 0.04 | 0.04 | 0.05 | 0.02 | 0.87 | 1.28 | 0.79 | 2.81 | 4.51 | 1.75 | 0.31 | 0.42 |
| 0.51 | 0.87 | 1.02 | 0.58 | 1.99 | 3.04 | 1.48 | 0.20 | 0.31 | 0.17 | 0.75 | 1.04 | 0.64 | 0.21 | 0.46 | 0.16 | 1.26 | 1.91 | 1.15 | 0.67 | 0.87 | 0.57 | 0.06 | 0.12 | 0.04 | 0.04 | 0.05 | 0.02 | 0.89 | 1.27 | 0.79 | 2.83 | 4.59 | 1.77 | 0.30 | 0.41 |
| 0.49 | 0.86 | 1.02 | 0.55 | 1.95 | 3.00 | 1.44 | 0.21 | 0.33 | 0.18 | 0.71 | 0.99 | 0.59 | 0.22 | 0.49 | 0.17 | 1.19 | 1.81 | 1.07 | 0.63 | 0.83 | 0.53 | 0.05 | 0.12 | 0.03 | 0.04 | 0.05 | 0.02 | 0.80 | 1.13 | 0.70 | 2.85 | 4.60 | 1.79 | 0.29 | 0.40 |
| 0.46 | 0.81 | 0.99 | 0.52 | 1.92 | 2.91 | 1.40 | 0.21 | 0.32 | 0.18 | 0.73 | 1.00 | 0.62 | 0.21 | 0.45 | 0.16 | 1.19 | 1.84 | 1.07 | 0.63 | 0.81 | 0.52 | 0.05 | 0.12 | 0.03 | 0.03 | 0.05 | 0.02 | 0.73 | 1.02 | 0.62 | 2.86 | 4.61 | 1.80 | 0.28 | 0.39 |
| 0.36 | 0.80 | 0.98 | 0.52 | 1.88 | 2.82 | 1.37 | 0.20 | 0.31 | 0.17 | 0.72 | 1.00 | 0.60 | 0.20 | 0.45 | 0.15 | 1.14 | 1.80 | 1.01 | 0.65 | 0.85 | 0.52 | 0.05 | 0.12 | 0.03 | 0.03 | 0.05 | 0.02 | 0.64 | 0.91 | 0.54 | 2.88 | 4.65 | 1.77 | 0.27 | 0.38 |
| 0.34 | 0.79 | 0.96 | 0.50 | 1.85 | 2.85 | 1.33 | 0.20 | 0.30 | 0.17 | 0.72 | 1.00 | 0.60 | 0.21 | 0.45 | 0.15 | 1.13 | 1.78 | 0.99 | 0.66 | 0.89 | 0.48 | 0.05 | 0.12 | 0.03 | 0.03 | 0.05 | 0.02 | 0.63 | 0.91 | 0.51 | 2.87 | 4.83 | 1.72 | 0.26 | 0.38 |
| 0.32 | 0.77 | 0.95 | 0.49 | 1.81 | 2.80 | 1.30 | 0.20 | 0.29 | 0.16 | 0.71 | 0.99 | 0.60 | 0.20 | 0.43 | 0.15 | 1.11 | 1.73 | 0.97 | 0.65 | 0.89 | 0.47 | 0.05 | 0.12 | 0.03 | 0.03 | 0.05 | 0.02 | 0.60 | 0.88 | 0.45 | 2.86 | 4.76 | 1.68 | 0.26 | 0.37 |
| 0.16 | 0.09 | 0.10 | 0.07 | 0.06 | 0.09 | 0.04 | 0.03 | 0.03 | 0.02 | 0.09 | 0.14 | 0.07 | 0.04 | 0.06 | 0.03 | 0.04 | 0.06 | 0.03 | 0.62 | 0.73 | 0.56 | 0.19 | 0.35 | 0.09 | 0.03 | 0.04 | 0.02 | 0.44 | 0.62 | 0.37 | 0.04 | 0.05 | 0.03 | 1.74 | 2.29 |
| 0.16 | 0.08 | 0.10 | 0.06 | 0.05 | 0.08 | 0.04 | 0.03 | 0.03 | 0.02 | 0.08 | 0.13 | 0.07 | 0.03 | 0.06 | 0.03 | 0.03 | 0.06 | 0.03 | 0.68 | 0.77 | 0.62 | 0.18 | 0.29 | 0.09 | 0.03 | 0.04 | 0.02 | 0.43 | 0.60 | 0.36 | 0.04 | 0.05 | 0.03 | 1.72 | 2.23 |
| 0.16 | 0.08 | 0.10 | 0.06 | 0.05 | 0.08 | 0.04 | 0.03 | 0.03 | 0.02 | 0.08 | 0.13 | 0.07 | 0.03 | 0.06 | 0.03 | 0.03 | 0.06 | 0.03 | 0.65 | 0.74 | 0.59 | 0.16 | 0.25 | 0.09 | 0.03 | 0.04 | 0.02 | 0.42 | 0.58 | 0.35 | 0.04 | 0.05 | 0.03 | 1.70 | 2.18 |
| 0.15 | 0.08 | 0.09 | 0.06 | 0.05 | 0.08 | 0.04 | 0.02 | 0.03 | 0.02 | 0.09 | 0.13 | 0.07 | 0.03 | 0.05 | 0.03 | 0.03 | 0.05 | 0.03 | 0.63 | 0.71 | 0.57 | 0.15 | 0.23 | 0.08 | 0.03 | 0.03 | 0.02 | 0.42 | 0.58 | 0.35 | 0.04 | 0.05 | 0.03 | 1.63 | 2.08 |
| 0.15 | 0.07 | 0.08 | 0.05 | 0.05 | 0.08 | 0.04 | 0.02 | 0.03 | 0.02 | 0.08 | 0.12 | 0.06 | 0.03 | 0.05 | 0.03 | 0.03 | 0.05 | 0.03 | 0.65 | 0.73 | 0.58 | 0.13 | 0.20 | 0.07 | 0.02 | 0.03 | 0.02 | 0.40 | 0.55 | 0.33 | 0.04 | 0.05 | 0.02 | 1.54 | 1.96 |
| 0.15 | 0.07 | 0.08 | 0.00 | 0.05 | 0.07 | 0.03 | 0.02 | 0.03 | 0.02 | 0.07 | 0.11 | 0.06 | 0.03 | 0.05 | 0.03 | 0.03 | 0.05 | 0.03 | 0.66 | 0.73 | 0.58 | 0.12 | 0.19 | 0.07 | 0.02 | 0.03 | 0.02 | 0.41 | 0.55 | 0.33 | 0.04 | 0.05 | 0.02 | 1.47 | 1.87 |
| 0.15 | 0.06 | 0.08 | 0.05 | 0.05 | 0.07 | 0.03 | 0.02 | 0.03 | 0.02 | 0.07 | 0.11 | 0.06 | 0.03 | 0.05 | 0.03 | 0.03 | 0.05 | 0.03 | 0.72 | 0.80 | 0.62 | 0.11 | 0.17 | 0.07 | 0.02 | 0.03 | 0.02 | 0.37 | 0.48 | 0.29 | 0.04 | 0.05 | 0.02 | 1.39 | 1.76 |
| 0.15 | 0.06 | 0.07 | 0.04 | 0.05 | 0.07 | 0.03 | 0.02 | 0.03 | 0.01 | 0.07 | 0.10 | 0.06 | 0.03 | 0.05 | 0.03 | 0.03 | 0.05 | 0.03 | 0.76 | 0.84 | 0.64 | 0.14 | 0.20 | 0.07 | 0.02 | 0.03 | 0.02 | 0.33 | 0.43 | 0.26 | 0.04 | 0.05 | 0.02 | 1.32 | 1.70 |
| 0.13 | 0.06 | 0.07 | 0.04 | 0.05 | 0.07 | 0.03 | 0.02 | 0.02 | 0.01 | 0.07 | 0.10 | 0.06 | 0.03 | 0.05 | 0.03 | 0.03 | 0.05 | 0.03 | 0.70 | 0.77 | 0.58 | 0.13 | 0.19 | 0.07 | 0.02 | 0.03 | 0.01 | 0.32 | 0.40 | 0.24 | 0.04 | 0.05 | 0.02 | 1.26 | 1.66 |
| 0.11 | 0.05 | 0.06 | 0.04 | 0.05 | 0.07 | 0.03 | 0.02 | 0.02 | 0.01 | 0.07 | 0.10 | 0.06 | 0.03 | 0.05 | 0.03 | 0.03 | 0.04 | 0.03 | 0.67 | 0.74 | 0.57 | 0.12 | 0.18 | 0.07 | 0.02 | 0.03 | 0.01 | 0.30 | 0.36 | 0.22 | 0.04 | 0.05 | 0.02 | 1.21 | 1.61 |
| 0.10 | 0.05 | 0.06 | 0.04 | 0.05 | 0.07 | 0.03 | 0.02 | 0.02 | 0.01 | 0.06 | 0.09 | 0.05 | 0.03 | 0.05 | 0.03 | 0.03 | 0.04 | 0.03 | 0.66 | 0.73 | 0.55 | 0.11 | 0.16 | 0.06 | 0.02 | 0.03 | 0.01 | 0.30 | 0.34 | 0.21 | 0.04 | 0.05 | 0.02 | 1.17 | 1.56 |
| 0.09 | 0.05 | 0.06 | 0.04 | 0.05 | 0.07 | 0.03 | 0.01 | 0.02 | 0.01 | 0.06 | 0.09 | 0.05 | 0.03 | 0.05 | 0.03 | 0.03 | 0.04 | 0.03 | 0.74 | 0.83 | 0.60 | 0.10 | 0.15 | 0.06 | 0.02 | 0.02 | 0.01 | 0.26 | 0.30 | 0.19 | 0.04 | 0.05 | 0.02 | 1.10 | 1.53 |
| 0.09 | 0.05 | 0.06 | 0.04 | 0.05 | 0.07 | 0.03 | 0.01 | 0.02 | 0.01 | 0.06 | 0.09 | 0.05 | 0.03 | 0.05 | 0.03 | 0.03 | 0.04 | 0.02 | 0.64 | 0.72 | 0.53 | 0.09 | 0.14 | 0.05 | 0.02 | 0.02 | 0.01 | 0.24 | 0.28 | 0.18 | 0.04 | 0.05 | 0.02 | 1.06 | 1.50 |
| 0.09 | 0.05 | 0.06 | 0.03 | 0.04 | 0.07 | 0.03 | 0.01 | 0.02 | 0.01 | 0.05 | 0.08 | 0.04 | 0.03 | 0.04 | 0.02 | 0.02 | 0.04 | 0.02 | 0.58 | 0.65 | 0.48 | 0.09 | 0.13 | 0.05 | 0.02 | 0.02 | 0.01 | 0.22 | 0.26 | 0.16 | 0.04 | 0.05 | 0.02 | 1.02 | 1.47 |
| 0.08 | 0.05 | 0.06 | 0.03 | 0.04 | 0.07 | 0.03 | 0.01 | 0.02 | 0.01 | 0.06 | 0.08 | 0.05 | 0.03 | 0.04 | 0.02 | 0.02 | 0.04 | 0.02 | 0.49 | 0.55 | 0.42 | 0.08 | 0.13 | 0.05 | 0.02 | 0.02 | 0.01 | 0.22 | 0.26 | 0.16 | 0.04 | 0.05 | 0.02 | 0.98 | 1.45 |
| 0.08 | 0.05 | 0.06 | 0.03 | 0.04 | 0.07 | 0.03 | 0.01 | 0.02 | 0.01 | 0.06 | 0.08 | 0.05 | 0.03 | 0.04 | 0.02 | 0.02 | 0.04 | 0.02 | 0.42 | 0.47 | 0.36 | 0.08 | 0.12 | 0.04 | 0.02 | 0.02 | 0.01 | 0.20 | 0.24 | 0.14 | 0.04 | 0.06 | 0.02 | 0.94 | 1.43 |
| 0.07 | 0.04 | 0.05 | 0.03 | 0.04 | 0.06 | 0.03 | 0.01 | 0.02 | 0.01 | 0.06 | 0.08 | 0.05 | 0.03 | 0.04 | 0.02 | 0.02 | 0.03 | 0.02 | 0.39 | 0.45 | 0.34 | 0.07 | 0.12 | 0.04 | 0.01 | 0.02 | 0.01 | 0.19 | 0.23 | 0.13 | 0.04 | 0.06 | 0.02 | 0.90 | 1.42 |
| 0.06 | 0.04 | 0.05 | 0.03 | 0.04 | 0.06 | 0.03 | 0.01 | 0.02 | 0.01 | 0.06 | 0.08 | 0.05 | 0.03 | 0.04 | 0.02 | 0.02 | 0.03 | 0.02 | 0.42 | 0.50 | 0.35 | 0.07 | 0.12 | 0.04 | 0.01 | 0.02 | 0.01 | 0.17 | 0.22 | 0.12 | 0.04 | 0.06 | 0.02 | 0.87 | 1.40 |
| 0.06 | 0.04 | 0.05 | 0.03 | 0.04 | 0.06 | 0.03 | 0.01 | 0.02 | 0.01 | 0.06 | 0.08 | 0.05 | 0.03 | 0.04 | 0.02 | 0.02 | 0.03 | 0.02 | 0.43 | 0.54 | 0.34 | 0.07 | 0.12 | 0.04 | 0.01 | 0.02 | 0.01 | 0.18 | 0.23 | 0.12 | 0.04 | 0.06 | 0.02 | 0.84 | 1.40 |
| 0.06 | 0.04 | 0.05 | 0.03 | 0.04 | 0.06 | 0.03 | 0.01 | 0.02 | 0.01 | 0.06 | 0.08 | 0.05 | 0.03 | 0.04 | 0.02 | 0.02 | 0.03 | 0.02 | 0.44 | 0.56 | 0.34 | 0.07 | 0.12 | 0.04 | 0.01 | 0.02 | 0.01 | 0.18 | 0.23 | 0.13 | 0.04 | 0.06 | 0.02 | 0.82 | 1.40 |

| | | | | | | | | | | | | | | | | | | | | | | | | | | | | | | | | | |
|---|---|---|---|---|---|---|---|---|---|---|---|---|---|---|---|---|---|---|---|---|---|---|---|---|---|---|---|---|---|---|---|---|---|
| Arabia | Scotland | | | Singapore | | | Slovakia | | | Slovenia | | | Sweden | | | Switzerland | | | Taiwan | | | UAE | | | UK | | | | U.S. | | Wales | | |
| | Scotlaı | AAPC | | Singap | AAPC | | Slovak | AAPC | | Sloven | AAPC | | Swede | AAPC | | Switze | AAPC | | Taiwaı | AAPC | | UAE | AAPC | | UK | AAPC | | U.S. | AAPC | | Wales | AAPC | |
| ##### | | -3.4 | ##### | | -3.9 | ##### | | -3.8 | ##### | | -4.9 | ##### | | -2.0 | ##### | | -5.5 | ##### | | -5.6 | ##### | | -0.9 | ##### | | -3.7 | ##### | | | | -1.0 | ##### | |
| ##### | -0.48 | **-1.4** | ##### | -0.08 | **-1.6** | ##### | -3.22 | **-2.5** | ##### | -6.25 | **-4.2** | ##### | -1.46 | **-1.2** | ##### | -7.21 | **-3.3** | ##### | -0.27 | **-1.3** | ##### | -2.43 | **-2.9** | ##### | -0.49 | **-2.2** | ##### | 5.71 | **0.5** | -100 | -0.54 | **-2.0** | ##### |
| | | | | | | | | | | | | | | | | | | | | | | | | | | Used WISQARS on USannual sheetinstead | | | | | | | |
| | | Ln | | | Ln | | | Ln | | | Ln | | | Ln | | | Ln | | | Ln | | | Ln | | | Ln | | | Ln | | | Ln | |
| | 0.52 | -0.65 | | 0.11 | -2.21 | | 2.38 | 0.87 | | 2.73 | 1.00 | | 1.76 | 0.57 | | 5.12 | 1.63 | | 0.43 | -0.84 | | 0.90 | -0.11 | | 0.35 | -1.05 | | 10.6 | 2.36 | | 0.42 | -0.87 | |
| | 0.54 | -0.62 | | 0.10 | -2.30 | | 2.21 | 0.79 | | 2.70 | 0.99 | | 1.73 | 0.55 | | 4.99 | 1.61 | | 0.40 | -0.92 | | 0.91 | -0.09 | | 0.35 | -1.05 | | 10.7 | 2.37 | | 0.41 | -0.89 | |
| | 0.54 | -0.62 | | 0.09 | -2.41 | | 2.15 | 0.77 | | 2.81 | 1.03 | | 1.69 | 0.52 | | 4.72 | 1.55 | | 0.46 | -0.78 | | 0.92 | -0.08 | | 0.34 | -1.08 | | 10.8 | 2.38 | | 0.39 | -0.94 | |
| | 0.49 | -0.71 | | 0.09 | -2.41 | | 2.05 | 0.72 | | 2.93 | 1.08 | | 1.55 | 0.44 | | 4.35 | 1.47 | | 0.39 | -0.94 | | 0.91 | -0.09 | | 0.33 | -1.11 | | 10.7 | 2.37 | | 0.40 | -0.92 | |
| | 0.47 | -0.76 | | 0.08 | -2.53 | | 1.88 | 0.63 | | 2.76 | 1.02 | | 1.56 | 0.44 | | 4.08 | 1.41 | | 0.40 | -0.92 | | 0.88 | -0.13 | | 0.32 | -1.14 | | 10.4 | 2.35 | | 0.38 | -0.97 | |
| | 0.42 | -0.87 | | 0.07 | -2.66 | | 1.80 | 0.59 | | 2.38 | 0.87 | | 1.42 | 0.35 | | 3.73 | 1.32 | | 0.41 | -0.89 | | 0.89 | -0.12 | | 0.30 | -1.20 | | 10.7 | 2.37 | | 0.35 | -1.05 | |
| | 0.48 | -0.73 | | 0.08 | -2.53 | | 1.66 | 0.51 | | 2.35 | 0.85 | | 1.38 | 0.32 | | 3.40 | 1.22 | | 0.35 | -1.05 | | 0.89 | -0.12 | | 0.31 | -1.17 | | 10.8 | 2.38 | | 0.37 | -0.99 | |
| | 0.44 | -0.82 | | 0.06 | -2.81 | | 1.63 | 0.49 | | 2.38 | 0.87 | | 1.43 | 0.36 | | 3.21 | 1.17 | | 0.31 | -1.17 | | 0.91 | -0.09 | | 0.28 | -1.27 | | 10.7 | 2.37 | | 0.34 | -1.08 | |
| | 0.44 | -0.82 | | 0.06 | -2.81 | | 1.60 | 0.47 | | 2.10 | 0.74 | | 1.38 | 0.32 | | 2.98 | 1.09 | | 0.25 | -1.39 | | 0.95 | -0.05 | | 0.29 | -1.24 | | 10.5 | 2.35 | | 0.34 | -1.08 | |
| | 0.38 | -0.97 | | 0.06 | -2.82 | | 1.50 | 0.41 | | 1.85 | 0.61 | | 1.39 | 0.33 | | 2.93 | 1.08 | | 0.27 | -1.31 | | 0.97 | -0.03 | | 0.25 | -1.37 | | 10.3 | 2.33 | | 0.30 | -1.19 | |
| | 0.38 | -0.97 | | 0.06 | -2.88 | | 1.41 | 0.35 | | 1.71 | 0.54 | | 1.29 | 0.26 | | 2.60 | 0.95 | | 0.23 | -1.49 | | 0.96 | -0.04 | | 0.25 | -1.40 | | 10.1 | 2.31 | | 0.30 | -1.20 | |
| | 0.37 | -0.99 | | 0.05 | -2.90 | | 1.40 | 0.34 | | 1.83 | 0.60 | | 1.28 | 0.25 | | 2.46 | 0.90 | | 0.18 | -1.70 | | 0.93 | -0.07 | | 0.24 | -1.44 | | 10.1 | 2.31 | | 0.30 | -1.20 | |
| | 0.32 | -1.13 | | 0.05 | -2.91 | | 1.36 | 0.31 | | 1.65 | 0.50 | | 1.24 | 0.21 | | 2.35 | 0.85 | | 0.21 | -1.58 | | 0.91 | -0.10 | | 0.21 | -1.57 | | 10.2 | 2.32 | | 0.27 | -1.29 | |
| | 0.32 | -1.15 | | 0.05 | -2.92 | | 1.29 | 0.26 | | 1.64 | 0.49 | | 1.27 | 0.24 | | 2.30 | 0.83 | | 0.19 | -1.65 | | 0.89 | -0.12 | | 0.21 | -1.55 | | 10.0 | 2.30 | | 0.27 | -1.29 | |
| | 0.28 | -1.28 | | 0.06 | -2.85 | | 1.29 | 0.25 | | 1.41 | 0.35 | | 1.28 | 0.25 | | 2.09 | 0.73 | | 0.19 | -1.68 | | 0.85 | -0.16 | | 0.20 | -1.63 | | 10.0 | 2.30 | | 0.24 | -1.43 | |
| | 0.28 | -1.26 | | 0.05 | -2.90 | | 1.30 | 0.26 | | 1.34 | 0.30 | | 1.30 | 0.26 | | 2.08 | 0.73 | | 0.18 | -1.71 | | 0.83 | -0.19 | | 0.20 | -1.63 | | 10.6 | 2.36 | | 0.25 | -1.37 | |
| | 0.30 | -1.19 | | 0.05 | -2.98 | | 1.19 | 0.17 | | 1.35 | 0.30 | | 1.21 | 0.19 | | 1.98 | 0.69 | | 0.17 | -1.80 | | 0.81 | -0.21 | | 0.19 | -1.64 | | 11.1 | 2.41 | | 0.25 | -1.38 | |
| | 0.33 | -1.11 | | 0.05 | -2.99 | | 1.16 | 0.15 | | 1.34 | 0.29 | | 1.26 | 0.23 | | 1.98 | 0.68 | | 0.18 | -1.70 | | 0.79 | -0.24 | | 0.19 | -1.65 | | 10.8 | 2.38 | | 0.25 | -1.40 | |
| | 0.33 | -1.11 | | 0.05 | -3.01 | | 1.16 | 0.15 | | 1.28 | 0.25 | | 1.16 | 0.15 | | 1.97 | 0.68 | | 0.19 | -1.66 | | 0.76 | -0.27 | | 0.20 | -1.61 | | 10.4 | 2.34 | | 0.26 | -1.36 | |
| | 0.32 | -1.13 | | 0.05 | -3.03 | | 1.15 | 0.14 | | 1.22 | 0.20 | | 1.13 | 0.12 | | 1.91 | 0.65 | | 0.19 | -1.65 | | 0.74 | -0.30 | | 0.20 | -1.61 | | 10.1 | 2.31 | | 0.26 | -1.35 | |

| lower | val | upper | lower | val | upper | lower | val | upper | lower | val | upper | lower | val | upper | lower | val | upper | lower | val | upper | lower | val | upper | lower | val | upper | lower | val | upper | lower | val | upper | lower |
|---|---|---|---|---|---|---|---|---|---|---|---|---|---|---|---|---|---|---|---|---|---|---|---|---|---|---|---|---|---|---|---|---|---|
| 0.06 | 0.12 | 0.13 | 0.11 | 0.03 | 0.03 | 0.02 | 0.70 | 0.81 | 0.60 | 0.49 | 0.52 | 0.46 | 0.27 | 0.28 | 0.25 | 0.35 | 0.37 | 0.33 | 0.32 | 0.35 | 0.29 | 0.40 | 0.56 | 0.26 | 0.07 | 0.07 | 0.07 | 4.46 | 4.51 | 4.41 | 0.08 | 0.08 | 0.07 |
| 0.06 | 0.14 | 0.15 | 0.13 | 0.02 | 0.02 | 0.02 | 0.61 | 0.71 | 0.53 | 0.38 | 0.41 | 0.35 | 0.23 | 0.25 | 0.22 | 0.53 | 0.57 | 0.50 | 0.29 | 0.32 | 0.27 | 0.41 | 0.57 | 0.27 | 0.08 | 0.09 | 0.08 | 4.60 | 4.65 | 4.55 | 0.09 | 0.10 | 0.08 |
| 0.06 | 0.15 | 0.16 | 0.13 | 0.02 | 0.02 | 0.02 | 0.57 | 0.67 | 0.49 | 0.50 | 0.53 | 0.46 | 0.24 | 0.25 | 0.22 | 0.46 | 0.49 | 0.43 | 0.35 | 0.38 | 0.32 | 0.41 | 0.56 | 0.27 | 0.08 | 0.09 | 0.08 | 4.70 | 4.75 | 4.65 | 0.09 | 0.10 | 0.08 |
| 0.07 | 0.15 | 0.16 | 0.13 | 0.03 | 0.03 | 0.03 | 0.52 | 0.61 | 0.45 | 0.45 | 0.48 | 0.42 | 0.22 | 0.24 | 0.21 | 0.37 | 0.39 | 0.35 | 0.30 | 0.33 | 0.28 | 0.41 | 0.56 | 0.27 | 0.09 | 0.09 | 0.08 | 4.68 | 4.74 | 4.61 | ## | ## | ## |
| 0.07 | 0.16 | 0.17 | 0.14 | 0.02 | 0.02 | 0.02 | 0.47 | 0.55 | 0.40 | 0.57 | 0.61 | 0.52 | 0.20 | 0.21 | 0.18 | 0.44 | 0.47 | 0.42 | 0.30 | 0.33 | 0.27 | 0.38 | 0.54 | 0.25 | 0.09 | 0.09 | 0.09 | 4.61 | 4.67 | 4.55 | ## | ## | ## |
| 0.08 | 0.13 | 0.14 | 0.12 | 0.01 | 0.02 | 0.01 | 0.42 | 0.50 | 0.36 | 0.39 | 0.42 | 0.36 | 0.18 | 0.19 | 0.17 | 0.35 | 0.37 | 0.33 | 0.30 | 0.33 | 0.28 | 0.39 | 0.55 | 0.25 | 0.08 | 0.08 | 0.08 | 4.80 | 4.86 | 4.73 | 0.09 | 0.10 | 0.08 |
| 0.09 | 0.15 | 0.17 | 0.14 | 0.02 | 0.02 | 0.02 | 0.39 | 0.47 | 0.34 | 0.20 | 0.22 | 0.18 | 0.19 | 0.21 | 0.18 | 0.26 | 0.27 | 0.24 | 0.25 | 0.28 | 0.23 | 0.39 | 0.54 | 0.25 | 0.08 | 0.08 | 0.08 | 4.89 | 4.97 | 4.82 | ## | ## | ## |
| 0.10 | 0.11 | 0.12 | 0.10 | 0.01 | 0.01 | 0.01 | 0.37 | 0.45 | 0.32 | 0.33 | 0.36 | 0.31 | 0.25 | 0.27 | 0.23 | 0.24 | 0.25 | 0.22 | 0.21 | 0.23 | 0.20 | 0.42 | 0.58 | 0.26 | 0.06 | 0.06 | 0.06 | 4.76 | 4.83 | 4.70 | ## | ## | ## |
| 0.11 | 0.12 | 0.13 | 0.11 | 0.01 | 0.01 | 0.01 | 0.33 | 0.40 | 0.28 | 0.22 | 0.23 | 0.20 | 0.22 | 0.23 | 0.20 | 0.22 | 0.24 | 0.21 | 0.16 | 0.18 | 0.15 | 0.45 | 0.64 | 0.26 | 0.07 | 0.07 | 0.07 | 4.54 | 4.60 | 4.47 | ## | ## | ## |
| 0.12 | 0.09 | 0.10 | 0.08 | 0.01 | 0.01 | 0.01 | 0.30 | 0.36 | 0.25 | 0.21 | 0.22 | 0.19 | 0.25 | 0.26 | 0.23 | 0.26 | 0.27 | 0.24 | 0.19 | 0.20 | 0.17 | 0.47 | 0.67 | 0.27 | 0.05 | 0.05 | 0.05 | 4.31 | 4.37 | 4.24 | 0.06 | 0.06 | 0.05 |
| 0.14 | 0.09 | 0.10 | 0.09 | 0.01 | 0.01 | 0.01 | 0.28 | 0.33 | 0.24 | 0.12 | 0.12 | 0.11 | 0.22 | 0.24 | 0.20 | 0.20 | 0.21 | 0.18 | 0.14 | 0.16 | 0.13 | 0.46 | 0.65 | 0.27 | 0.05 | 0.06 | 0.05 | 4.14 | 4.19 | 4.08 | 0.06 | 0.07 | 0.06 |
| 0.14 | 0.11 | 0.12 | 0.10 | 0.01 | 0.01 | 0.01 | 0.28 | 0.33 | 0.24 | 0.34 | 0.36 | 0.32 | 0.23 | 0.24 | 0.21 | 0.21 | 0.22 | 0.20 | 0.09 | 0.10 | 0.08 | 0.43 | 0.63 | 0.25 | 0.06 | 0.06 | 0.06 | 4.10 | 4.15 | 4.05 | 0.07 | 0.08 | 0.07 |
| 0.15 | 0.07 | 0.08 | 0.06 | 0.01 | 0.01 | 0.01 | 0.27 | 0.32 | 0.23 | 0.20 | 0.22 | 0.19 | 0.21 | 0.22 | 0.19 | 0.21 | 0.22 | 0.20 | 0.12 | 0.13 | 0.11 | 0.41 | 0.61 | 0.24 | 0.04 | 0.04 | 0.04 | 4.19 | 4.26 | 4.11 | 0.05 | 0.05 | 0.05 |
| 0.15 | 0.07 | 0.08 | 0.07 | 0.01 | 0.01 | 0.01 | 0.26 | 0.31 | 0.22 | 0.28 | 0.31 | 0.26 | 0.25 | 0.27 | 0.23 | 0.20 | 0.22 | 0.19 | 0.11 | 0.12 | 0.10 | 0.39 | 0.60 | 0.23 | 0.05 | 0.05 | 0.04 | 3.95 | 4.01 | 3.88 | 0.06 | 0.06 | 0.05 |
| 0.14 | 0.06 | 0.06 | 0.05 | 0.02 | 0.02 | 0.02 | 0.27 | 0.32 | 0.22 | 0.25 | 0.27 | 0.23 | 0.28 | 0.30 | 0.26 | 0.14 | 0.15 | 0.13 | 0.11 | 0.12 | 0.10 | 0.37 | 0.61 | 0.20 | 0.04 | 0.04 | 0.04 | 3.88 | 3.96 | 3.79 | 0.04 | 0.05 | 0.04 |
| 0.14 | 0.05 | 0.06 | 0.05 | 0.01 | 0.02 | 0.01 | 0.27 | 0.33 | 0.23 | 0.09 | 0.10 | 0.08 | 0.31 | 0.33 | 0.28 | 0.19 | 0.21 | 0.18 | 0.10 | 0.11 | 0.09 | 0.36 | 0.61 | 0.18 | 0.03 | 0.04 | 0.03 | 4.36 | 4.45 | 4.26 | 0.04 | 0.05 | 0.04 |
| 0.13 | 0.05 | 0.06 | 0.05 | 0.01 | 0.01 | 0.01 | 0.26 | 0.31 | 0.21 | 0.16 | 0.18 | 0.15 | 0.28 | 0.31 | 0.26 | 0.15 | 0.17 | 0.14 | 0.09 | 0.10 | 0.08 | 0.34 | 0.61 | 0.15 | 0.03 | 0.03 | 0.03 | 4.65 | 4.81 | 4.47 | 0.04 | 0.04 | 0.03 |
| 0.12 | 0.07 | 0.07 | 0.06 | 0.01 | 0.02 | 0.01 | 0.25 | 0.32 | 0.20 | 0.17 | 0.20 | 0.15 | 0.34 | 0.36 | 0.31 | 0.17 | 0.19 | 0.16 | 0.10 | 0.12 | 0.09 | 0.32 | 0.60 | 0.14 | 0.03 | 0.03 | 0.03 | 4.47 | 4.58 | 4.15 | 0.04 | 0.04 | 0.03 |
| 0.12 | 0.07 | 0.07 | 0.06 | 0.01 | 0.01 | 0.01 | 0.25 | 0.34 | 0.19 | 0.18 | 0.22 | 0.14 | 0.27 | 0.29 | 0.24 | 0.18 | 0.20 | 0.17 | 0.11 | 0.14 | 0.09 | 0.31 | 0.61 | 0.13 | 0.04 | 0.04 | 0.04 | 4.24 | 4.37 | 3.96 | 0.04 | 0.05 | 0.04 |
| 0.11 | 0.07 | 0.08 | 0.06 | 0.01 | 0.01 | 0.01 | 0.25 | 0.34 | 0.18 | 0.18 | 0.23 | 0.14 | 0.25 | 0.28 | 0.23 | 0.18 | 0.20 | 0.17 | 0.11 | 0.14 | 0.09 | 0.30 | 0.58 | 0.12 | 0.04 | 0.04 | 0.04 | 4.12 | 4.25 | 3.83 | 0.05 | 0.05 | 0.04 |
| 0.15 | 0.36 | 0.84 | 0.27 | 0.06 | 0.07 | 0.04 | 1.37 | 1.82 | 0.76 | 2.20 | 3.06 | 1.92 | 1.44 | 1.92 | 1.29 | 4.65 | 5.34 | 4.03 | 0.07 | 0.10 | 0.06 | 0.37 | 0.63 | 0.19 | 0.25 | 0.42 | 0.22 | 5.79 | 6.24 | 5.19 | 0.31 | 0.59 | 0.24 |
| 0.16 | 0.36 | 0.79 | 0.27 | 0.05 | 0.06 | 0.04 | 1.31 | 1.76 | 0.72 | 2.27 | 3.09 | 1.97 | 1.45 | 1.91 | 1.28 | 4.35 | 5.01 | 3.72 | 0.07 | 0.10 | 0.06 | 0.37 | 0.64 | 0.19 | 0.24 | 0.40 | 0.22 | 5.76 | 6.23 | 5.17 | 0.29 | 0.54 | 0.23 |
| 0.17 | 0.35 | 0.77 | 0.27 | 0.05 | 0.06 | 0.03 | 1.30 | 1.71 | 0.70 | 2.27 | 3.02 | 1.95 | 1.41 | 1.87 | 1.24 | 4.16 | 4.81 | 3.52 | 0.07 | 0.10 | 0.06 | 0.38 | 0.66 | 0.19 | 0.23 | 0.38 | 0.21 | 5.76 | 6.23 | 5.20 | 0.27 | 0.51 | 0.22 |
| 0.17 | 0.30 | 0.64 | 0.24 | 0.04 | 0.05 | 0.03 | 1.27 | 1.65 | 0.66 | 2.44 | 3.17 | 2.07 | 1.29 | 1.74 | 1.12 | 3.88 | 4.45 | 3.24 | 0.06 | 0.10 | 0.05 | 0.37 | 0.60 | 0.18 | 0.21 | 0.34 | 0.20 | 5.68 | 6.17 | 5.12 | ## | ## | ## |
| 0.17 | 0.28 | 0.58 | 0.22 | 0.04 | 0.05 | 0.03 | 1.18 | 1.56 | 0.62 | 2.16 | 2.72 | 1.75 | 1.32 | 1.76 | 1.16 | 3.55 | 4.10 | 2.97 | 0.07 | 0.11 | 0.06 | 0.37 | 0.61 | 0.17 | 0.20 | 0.31 | 0.18 | 5.53 | 6.03 | 5.00 | ## | ## | ## |
| 0.17 | 0.26 | 0.54 | 0.20 | 0.04 | 0.04 | 0.02 | 1.17 | 1.57 | 0.61 | 1.96 | 2.48 | 1.55 | 1.20 | 1.63 | 1.06 | 3.30 | 3.84 | 2.73 | 0.08 | 0.13 | 0.00 | 0.37 | 0.62 | 0.17 | 0.19 | 0.30 | 0.17 | 5.62 | 6.14 | 5.09 | 0.23 | 0.40 | 0.19 |
| 0.17 | 0.30 | 0.63 | 0.23 | 0.04 | 0.04 | 0.02 | 1.09 | 1.52 | 0.58 | 2.12 | 2.61 | 1.64 | 1.15 | 1.59 | 1.01 | 3.07 | 3.55 | 2.51 | 0.07 | 0.13 | 0.06 | 0.37 | 0.62 | 0.17 | 0.20 | 0.31 | 0.18 | 5.66 | 6.23 | 5.19 | ## | ## | ## |
| 0.17 | 0.30 | 0.63 | 0.23 | 0.03 | 0.04 | 0.02 | 1.08 | 1.49 | 0.59 | 2.02 | 2.38 | 1.49 | 1.14 | 1.57 | 1.00 | 2.90 | 3.32 | 2.35 | 0.07 | 0.12 | 0.06 | 0.36 | 0.62 | 0.17 | 0.19 | 0.30 | 0.18 | 5.66 | 6.29 | 5.22 | ## | ## | ## |
| 0.16 | 0.29 | 0.62 | 0.23 | 0.03 | 0.04 | 0.02 | 1.06 | 1.45 | 0.57 | 1.85 | 2.17 | 1.31 | 1.12 | 1.57 | 0.98 | 2.70 | 3.10 | 2.14 | 0.06 | 0.11 | 0.05 | 0.37 | 0.63 | 0.18 | 0.19 | 0.30 | 0.17 | 5.71 | 6.37 | 5.30 | ## | ## | ## |
| 0.16 | 0.26 | 0.55 | 0.20 | 0.03 | 0.04 | 0.02 | 0.99 | 1.36 | 0.56 | 1.62 | 1.87 | 1.11 | 1.11 | 1.56 | 0.97 | 2.62 | 2.98 | 2.04 | 0.06 | 0.10 | 0.05 | 0.37 | 0.63 | 0.18 | 0.18 | 0.28 | 0.16 | 5.73 | 6.43 | 5.34 | 0.22 | 0.38 | 0.18 |
| 0.16 | 0.25 | 0.53 | 0.20 | 0.03 | 0.03 | 0.02 | 0.93 | 1.25 | 0.52 | 1.57 | 1.80 | 1.08 | 1.04 | 1.45 | 0.90 | 2.35 | 2.69 | 1.81 | 0.06 | 0.10 | 0.05 | 0.37 | 0.64 | 0.17 | 0.17 | 0.27 | 0.15 | 5.69 | 6.44 | 5.35 | 0.21 | 0.37 | 0.17 |
| 0.16 | 0.24 | 0.50 | 0.19 | 0.03 | 0.03 | 0.02 | 0.91 | 1.22 | 0.51 | 1.47 | 1.71 | 0.99 | 1.02 | 1.43 | 0.88 | 2.20 | 2.52 | 1.66 | 0.07 | 0.11 | 0.06 | 0.37 | 0.64 | 0.17 | 0.15 | 0.25 | 0.14 | 5.78 | 6.56 | 5.44 | 0.20 | 0.37 | 0.17 |
| 0.15 | 0.22 | 0.47 | 0.18 | 0.03 | 0.03 | 0.02 | 0.88 | 1.19 | 0.48 | 1.43 | 1.68 | 0.97 | 1.00 | 1.39 | 0.85 | 2.09 | 2.42 | 1.57 | 0.06 | 0.10 | 0.05 | 0.36 | 0.65 | 0.16 | 0.14 | 0.24 | 0.13 | 5.78 | 6.59 | 5.46 | 0.20 | 0.37 | 0.16 |
| 0.15 | 0.21 | 0.44 | 0.17 | 0.03 | 0.03 | 0.02 | 0.83 | 1.13 | 0.46 | 1.33 | 1.58 | 0.90 | 0.99 | 1.37 | 0.85 | 2.05 | 2.38 | 1.54 | 0.06 | 0.09 | 0.05 | 0.36 | 0.64 | 0.16 | 0.14 | 0.24 | 0.13 | 5.80 | 6.62 | 5.48 | 0.19 | 0.36 | 0.16 |
| 0.15 | 0.19 | 0.38 | 0.15 | 0.02 | 0.03 | 0.02 | 0.82 | 1.13 | 0.45 | 1.14 | 1.38 | 0.75 | 0.97 | 1.34 | 0.82 | 1.90 | 2.20 | 1.42 | 0.06 | 0.09 | 0.05 | 0.35 | 0.64 | 0.15 | 0.14 | 0.22 | 0.12 | 5.86 | 6.70 | 5.55 | 0.17 | 0.32 | 0.14 |
| 0.15 | 0.20 | 0.41 | 0.16 | 0.02 | 0.03 | 0.01 | 0.83 | 1.13 | 0.47 | 1.24 | 1.48 | 0.82 | 0.97 | 1.33 | 0.81 | 1.85 | 2.12 | 1.36 | 0.06 | 0.09 | 0.05 | 0.34 | 0.62 | 0.15 | 0.14 | 0.23 | 0.13 | 6.03 | 6.87 | 5.71 | 0.19 | 0.35 | 0.15 |
| 0.15 | 0.22 | 0.45 | 0.18 | 0.02 | 0.03 | 0.01 | 0.76 | 1.10 | 0.44 | 1.17 | 1.43 | 0.78 | 0.90 | 1.24 | 0.75 | 1.79 | 2.06 | 1.31 | 0.06 | 0.09 | 0.05 | 0.34 | 0.61 | 0.15 | 0.14 | 0.23 | 0.13 | 6.26 | 7.08 | 5.93 | 0.19 | 0.36 | 0.16 |
| 0.14 | 0.23 | 0.48 | 0.18 | 0.02 | 0.02 | 0.01 | 0.74 | 1.09 | 0.43 | 1.15 | 1.50 | 0.76 | 0.90 | 1.22 | 0.74 | 1.77 | 2.05 | 1.30 | 0.06 | 0.09 | 0.05 | 0.33 | 0.61 | 0.14 | 0.14 | 0.23 | 0.13 | 6.11 | 6.86 | 5.78 | 0.19 | 0.35 | 0.15 |
| 0.13 | 0.23 | 0.47 | 0.18 | 0.02 | 0.02 | 0.01 | 0.74 | 1.12 | 0.42 | 1.09 | 1.48 | 0.71 | 0.86 | 1.19 | 0.72 | 1.76 | 2.05 | 1.28 | 0.06 | 0.09 | 0.04 | 0.33 | 0.60 | 0.14 | 0.14 | 0.23 | 0.13 | 5.93 | 6.69 | 5.62 | 0.19 | 0.35 | 0.15 |
| 0.13 | 0.23 | 0.46 | 0.17 | 0.02 | 0.03 | 0.01 | 0.73 | 1.10 | 0.43 | 1.02 | 1.45 | 0.67 | 0.85 | 1.17 | 0.70 | 1.69 | 1.99 | 1.23 | 0.06 | 0.09 | 0.04 | 0.33 | 0.60 | 0.14 | 0.14 | 0.22 | 0.12 | 5.76 | 6.50 | 5.42 | 0.19 | 0.36 | 0.15 |
| 0.98 | 0.04 | 0.06 | 0.03 | 0.02 | 0.03 | 0.02 | 0.31 | 0.37 | 0.17 | 0.04 | 0.07 | 0.04 | 0.05 | 0.06 | 0.03 | 0.12 | 0.14 | 0.07 | 0.04 | 0.06 | 0.04 | 0.13 | 0.48 | 0.03 | 0.03 | 0.04 | 0.03 | 0.35 | 0.41 | 0.29 | 0.03 | 0.06 | 0.03 |
| 0.96 | 0.04 | 0.05 | 0.03 | 0.03 | 0.03 | 0.02 | 0.29 | 0.35 | 0.16 | 0.05 | 0.07 | 0.04 | 0.05 | 0.05 | 0.03 | 0.11 | 0.12 | 0.06 | 0.04 | 0.06 | 0.03 | 0.13 | 0.47 | 0.03 | 0.03 | 0.04 | 0.03 | 0.34 | 0.39 | 0.28 | 0.03 | 0.05 | 0.03 |
| 0.95 | 0.04 | 0.05 | 0.03 | 0.02 | 0.03 | 0.02 | 0.28 | 0.34 | 0.16 | 0.04 | 0.07 | 0.03 | 0.04 | 0.05 | 0.03 | 0.10 | 0.11 | 0.06 | 0.04 | 0.05 | 0.03 | 0.13 | 0.46 | 0.03 | 0.03 | 0.04 | 0.03 | 0.33 | 0.38 | 0.28 | 0.03 | 0.05 | 0.03 |
| 0.94 | 0.04 | 0.05 | 0.03 | 0.02 | 0.02 | 0.02 | 0.26 | 0.32 | 0.16 | 0.04 | 0.06 | 0.03 | 0.04 | 0.05 | 0.03 | 0.10 | 0.11 | 0.06 | 0.03 | 0.05 | 0.03 | 0.13 | 0.44 | 0.03 | 0.03 | 0.04 | 0.03 | 0.31 | 0.37 | 0.26 | ## | ## | ## |
| 0.89 | 0.03 | 0.05 | 0.03 | 0.02 | 0.02 | 0.02 | 0.23 | 0.30 | 0.15 | 0.03 | 0.06 | 0.03 | 0.04 | 0.05 | 0.03 | 0.09 | 0.10 | 0.06 | 0.03 | 0.05 | 0.03 | 0.13 | 0.43 | 0.03 | 0.03 | 0.04 | 0.03 | 0.30 | 0.36 | 0.25 | ## | ## | ## |
| 0.87 | 0.03 | 0.05 | 0.03 | 0.02 | 0.02 | 0.01 | 0.21 | 0.30 | 0.14 | 0.03 | 0.06 | 0.02 | 0.04 | 0.05 | 0.03 | 0.08 | 0.09 | 0.05 | 0.03 | 0.05 | 0.03 | 0.13 | 0.41 | 0.03 | 0.03 | 0.04 | 0.03 | 0.30 | 0.35 | 0.25 | ## | ## | ## |
| 0.83 | 0.03 | 0.05 | 0.03 | 0.02 | 0.02 | 0.01 | 0.18 | 0.29 | 0.13 | 0.03 | 0.06 | 0.02 | 0.04 | 0.05 | 0.03 | 0.07 | 0.08 | 0.05 | 0.03 | 0.05 | 0.03 | 0.13 | 0.42 | 0.03 | 0.03 | 0.04 | 0.03 | 0.28 | 0.34 | 0.24 | 0.03 | 0.05 | 0.03 |
| 0.80 | 0.03 | 0.05 | 0.03 | 0.02 | 0.02 | 0.01 | 0.18 | 0.29 | 0.13 | 0.03 | 0.05 | 0.02 | 0.04 | 0.05 | 0.03 | 0.07 | 0.08 | 0.05 | 0.03 | 0.04 | 0.03 | 0.13 | 0.43 | 0.03 | 0.03 | 0.04 | 0.02 | 0.27 | 0.33 | 0.23 | ## | ## | ## |
| 0.76 | 0.03 | 0.05 | 0.03 | 0.02 | 0.02 | 0.01 | 0.21 | 0.28 | 0.13 | 0.03 | 0.05 | 0.02 | 0.04 | 0.04 | 0.03 | 0.06 | 0.07 | 0.04 | 0.03 | 0.04 | 0.02 | 0.13 | 0.45 | 0.03 | 0.03 | 0.04 | 0.02 | 0.25 | 0.31 | 0.22 | ## | ## | ## |
| 0.71 | 0.03 | 0.04 | 0.03 | 0.02 | 0.02 | 0.01 | 0.21 | 0.27 | 0.12 | 0.03 | 0.05 | 0.02 | 0.03 | 0.04 | 0.02 | 0.05 | 0.07 | 0.04 | 0.03 | 0.04 | 0.02 | 0.14 | 0.47 | 0.04 | 0.03 | 0.03 | 0.02 | 0.24 | 0.29 | 0.21 | 0.03 | 0.04 | 0.02 |
| 0.70 | 0.03 | 0.04 | 0.03 | 0.02 | 0.02 | 0.01 | 0.21 | 0.26 | 0.11 | 0.02 | 0.04 | 0.02 | 0.04 | 0.04 | 0.02 | 0.05 | 0.06 | 0.04 | 0.02 | 0.04 | 0.02 | 0.14 | 0.47 | 0.03 | 0.03 | 0.03 | 0.02 | 0.23 | 0.28 | 0.20 | 0.03 | 0.04 | 0.02 |
| 0.64 | 0.03 | 0.04 | 0.02 | 0.02 | 0.02 | 0.01 | 0.21 | 0.26 | 0.11 | 0.02 | 0.04 | 0.02 | 0.03 | 0.04 | 0.02 | 0.05 | 0.06 | 0.04 | 0.02 | 0.04 | 0.02 | 0.14 | 0.49 | 0.04 | 0.02 | 0.03 | 0.02 | 0.22 | 0.27 | 0.20 | 0.03 | 0.04 | 0.02 |
| 0.61 | 0.03 | 0.04 | 0.02 | 0.02 | 0.02 | 0.01 | 0.21 | 0.26 | 0.10 | 0.02 | 0.04 | 0.02 | 0.03 | 0.04 | 0.02 | 0.04 | 0.06 | 0.04 | 0.02 | 0.03 | 0.02 | 0.14 | 0.49 | 0.04 | 0.02 | 0.03 | 0.02 | 0.21 | 0.26 | 0.19 | 0.03 | 0.04 | 0.02 |
| 0.57 | 0.03 | 0.04 | 0.02 | 0.02 | 0.02 | 0.01 | 0.20 | 0.26 | 0.10 | 0.02 | 0.04 | 0.02 | 0.03 | 0.04 | 0.02 | 0.04 | 0.06 | 0.04 | 0.02 | 0.03 | 0.02 | 0.14 | 0.50 | 0.04 | 0.02 | 0.03 | 0.02 | 0.21 | 0.26 | 0.18 | 0.03 | 0.04 | 0.02 |
| 0.56 | 0.03 | 0.03 | 0.02 | 0.02 | 0.02 | 0.01 | 0.20 | 0.26 | 0.09 | 0.02 | 0.04 | 0.02 | 0.03 | 0.03 | 0.02 | 0.04 | 0.05 | 0.03 | 0.02 | 0.03 | 0.02 | 0.13 | 0.51 | 0.03 | 0.02 | 0.03 | 0.02 | 0.21 | 0.26 | 0.18 | 0.02 | 0.04 | 0.02 |
| 0.52 | 0.03 | 0.03 | 0.02 | 0.02 | 0.02 | 0.01 | 0.20 | 0.26 | 0.09 | 0.02 | 0.04 | 0.02 | 0.03 | 0.03 | 0.02 | 0.03 | 0.05 | 0.03 | 0.02 | 0.03 | 0.02 | 0.13 | 0.49 | 0.03 | 0.02 | 0.03 | 0.02 | 0.21 | 0.26 | 0.18 | 0.02 | 0.04 | 0.02 |
| 0.50 | 0.03 | 0.04 | 0.02 | 0.01 | 0.02 | 0.01 | 0.18 | 0.24 | 0.08 | 0.02 | 0.04 | 0.02 | 0.03 | 0.03 | 0.02 | 0.04 | 0.05 | 0.03 | 0.02 | 0.03 | 0.02 | 0.13 | 0.48 | 0.03 | 0.02 | 0.03 | 0.02 | 0.21 | 0.26 | 0.18 | 0.02 | 0.04 | 0.02 |
| 0.48 | 0.03 | 0.04 | 0.02 | 0.01 | 0.02 | 0.01 | 0.17 | 0.24 | 0.08 | 0.02 | 0.04 | 0.01 | 0.03 | 0.03 | 0.02 | 0.04 | 0.05 | 0.03 | 0.02 | 0.03 | 0.02 | 0.13 | 0.48 | 0.03 | 0.02 | 0.03 | 0.02 | 0.21 | 0.26 | 0.18 | 0.02 | 0.04 | 0.02 |
| 0.46 | 0.03 | 0.04 | 0.02 | 0.01 | 0.02 | 0.01 | 0.17 | 0.25 | 0.08 | 0.02 | 0.04 | 0.01 | 0.03 | 0.03 | 0.02 | 0.04 | 0.05 | 0.03 | 0.02 | 0.03 | 0.02 | 0.12 | 0.47 | 0.03 | 0.02 | 0.03 | 0.02 | 0.20 | 0.25 | 0.17 | 0.02 | 0.04 | 0.02 |
| 0.44 | 0.03 | 0.04 | 0.02 | 0.01 | 0.02 | 0.01 | 0.16 | 0.24 | 0.08 | 0.02 | 0.04 | 0.01 | 0.03 | 0.03 | 0.02 | 0.04 | 0.05 | 0.03 | 0.02 | 0.03 | 0.02 | 0.12 | 0.48 | 0.03 | 0.02 | 0.03 | 0.02 | 0.20 | 0.24 | 0.17 | 0.02 | 0.04 | 0.02 |
