# Supplementary material for: Fallacy of attributing the U.S. firearm mortality epidemic to mental health
Source: PLoS One. 2024 Aug 5;19(8):e0290138. doi: 10.1371/journal.pone.0290138 (PMC11299823; doi:10.1371/journal.pone.0290138)
Supplement: S2 File — (PDF) [file pone.0290138.s004.pdf]

FA Deaths

	M&F Deaths	M&F Pop	M&F Age Adj Ra	M&F Lo CI	M&F Hi CI	Female Deaths	Female Pop	Female Age Adj Ra	Female Lo CI	Female Hi CI	Male Deaths	Male Pop	Male Age Adj Ra	Male Lo CI	Male Hi CI
2000	28,663	281,421,906	10.16	10.04	10.28	4,081	143,368,343	2.84	2.76	2.93	24,582	138,053,563	18.11	17.88	18.34
2001	29,573	284,968,955	10.32	10.20	10.43	4,093	145,077,463	2.81	2.72	2.89	25,480	139,891,492	18.46	18.23	18.69
2002	30,242	287,625,193	10.45	10.34	10.57	4,144	146,394,634	2.82	2.73	2.90	26,098	141,230,559	18.72	18.49	18.94
2003	30,136	290,107,933	10.31	10.19	10.42	4,012	147,679,036	2.70	2.62	2.79	26,124	142,428,897	18.50	18.28	18.73
2004	29,569	292,805,298	10.01	9.90	10.13	4,071	148,977,286	2.72	2.64	2.80	25,498	143,828,012	17.88	17.66	18.10
2005	30,694	295,516,599	10.30	10.19	10.42	4,037	150,319,521	2.68	2.60	2.76	26,657	145,197,078	18.45	18.23	18.68
2006	30,896	298,379,912	10.27	10.15	10.38	4,184	151,732,647	2.73	2.64	2.81	26,712	146,647,265	18.24	18.02	18.46
2007	31,224	301,231,207	10.28	10.16	10.39	4,177	153,166,353	2.71	2.63	2.80	27,047	148,064,854	18.29	18.07	18.51
2008	31,593	304,093,966	10.26	10.15	10.37	4,257	154,604,015	2.74	2.65	2.82	27,336	149,489,951	18.25	18.03	18.47
2009	31,347	306,771,529	10.07	9.96	10.18	4,426	155,964,075	2.81	2.73	2.90	26,921	150,807,454	17.83	17.61	18.04
2010	31,672	308,745,538	10.10	9.99	10.21	4,316	156,964,212	2.70	2.62	2.79	27,356	151,781,326	17.94	17.72	18.15
2011	32,351	311,591,917	10.22	10.11	10.34	4,613	158,301,098	2.88	2.79	2.96	27,738	153,290,819	17.96	17.75	18.18
2012	33,563	313,914,040	10.51	10.40	10.63	4,725	159,421,973	2.95	2.87	3.04	28,838	154,492,067	18.49	18.28	18.71
2013	33,636	316,128,839	10.43	10.32	10.54	4,842	160,477,237	2.98	2.89	3.06	28,794	155,651,602	18.33	18.12	18.55
2014	33,594	318,857,056	10.31	10.20	10.42	4,879	161,920,569	2.98	2.90	3.07	28,715	156,936,487	18.04	17.83	18.26
2015	36,252	321,418,820	11.06	10.94	11.18	5,220	163,189,523	3.16	3.07	3.24	31,032	158,229,297	19.36	19.14	19.58
2016	38,658	323,127,513	11.78	11.66	11.90	5,664	164,048,590	3.42	3.33	3.51	32,994	159,078,923	20.50	20.27	20.72
2017	39,773	325,719,178	11.99	11.87	12.11	5,711	165,311,059	3.42	3.33	3.51	34,062	160,408,119	20.95	20.72	21.17
2018	39,740	327,167,434	11.90	11.78	12.02	5,785	166,038,755	3.44	3.35	3.53	33,955	161,128,679	20.71	20.48	20.93
2019	39,707	328,239,523	11.86	11.74	11.98	5,666	166,582,199	3.39	3.30	3.48	34,041	161,657,324	20.70	20.48	20.92
2020	45,222	329,484,123	13.62	13.49	13.75	6,241	167,227,921	3.77	3.67	3.86	38,981	162,256,202	23.80	23.56	24.04
2021	48,830	331,893,745	14.65	14.51	14.78										
			35%	increase in last decade											

	**WISQARS**					**CDC WONDER**	
	Deaths	Pop	AgeAdj	Ln		AgeAdj	Ln
2000	28,663	282,171,936	10.11	2.313525		10.161	2.31855686
2001	29,573	284,968,955	10.31	2.333114		10.315	2.33359915
2002	30,242	287,625,193	10.43	2.344686		10.453	2.34688902
2003	30,136	290,107,933	10.29	2.331173		10.307	2.33282328
2004	29,569	292,805,298	9.99	2.301585		10.011	2.30368449
2005	30,694	295,516,599	10.27	2.329227		10.3	2.3321439
2006	30,896	298,379,912	10.22	2.324347		10.267	2.32893487
2007	31,224	301,231,207	10.24	2.326302		10.279	2.33010298
2008	31,593	304,093,966	10.23	2.325325		10.259	2.32815537
2009	31,347	306,771,529	10.05	2.307573		10.069	2.3094614
2010	31,672	308,758,105	10.07	2.309561		10.1	2.31253542
2011	32,351	311,583,481	10.16	2.318458		10.223	2.32464008
2012	33,563	313,877,662	10.45	2.346602		10.512	2.35251746
2013	33,636	316,059,947	10.38	2.339881		10.429	2.34459039
2014	33,594	318,386,329	10.27	2.329227		10.312	2.33330827
2015	36,252	320,738,994	11.03	2.400619		11.06	2.403335
2016	38,658	323,071,755	11.73	2.46215		11.776	2.46606356
2017	39,773	325,122,128	11.97	2.482404		11.994	2.48440652
2018	39,740	326,838,199	11.86	2.473171		11.9	2.4765384
2019	39,707	328,329,953	11.83	2.470639		11.859	2.47308707
2020	45,222	329,484,123	13.58	2.608598		13.618	2.61139245
			AAPC 2010-2020	**2.7**	-100	**2.6**	-100
