# Supplementary material for: Fallacy of attributing the U.S. firearm mortality epidemic to mental health
Source: PLoS One. 2024 Aug 5;19(8):e0290138. doi: 10.1371/journal.pone.0290138 (PMC11299823; doi:10.1371/journal.pone.0290138)
Supplement: S3 File — (PDF) [file pone.0290138.s005.pdf]

IHME		Hi SDI Rate	Hi CI	Lo CI	Deaths	% of all Deaths	U.S. AA Rate	Hi CI	Lo CI	Deaths	Pop	% of all Deaths	I excluding U.S. S. Pop Mill	DI Pop Mill	Pop	Deaths
**All Firearm**	1990	5.66					13.85									
**Deaths**	1991	5.72					14.07									
	1992	5.65					13.86									
	1993	5.73					14.14									
	1994	5.62					13.87									
	1995	5.38					13.23									
	1996	5.07					12.39									
	1997	4.82					11.75									
	1998	4.59					11.16									
	1999	4.43					10.31			28,874	279,040,168					
	2000	4.33					10.16			28,663	281,421,906					
	2001	4.33					10.31			29,573	284,968,955					
	2002	4.32					10.45			30,242	287,625,193					
	2003	4.27					10.31			30,136	290,107,933					
	2004	4.13					10.01			29,569	292,805,298					
	2005	4.21					10.3			30,694	295,516,599					
	2006	4.21					10.27			30,896	298,379,912					
	2007	4.15					10.28			31,224	301,231,207					
	2008	4.08					10.26			31,593	304,093,966					
	2009	4.01					10.07			31,347	306,771,529					
	2010	3.92					10.1			31,672	308,745,538					
	2011	3.92					10.22			32,351	311,591,917					
	2012	3.94					10.51			33,563	313,914,040					
	2013	3.83					10.43			33,636	316,128,839					
	2014	3.80					10.31			33,594	318,857,056					
	2015	4.01					11.06			36,252	321,418,820					
	2016	4.17					11.78			38,658	323,127,513					
	2017	4.07					11.99			39,773	325,719,178					
	2018	3.93					11.9			39,740	327,167,434	% of all				
	2019	3.84					11.86			39,707	328,239,523	all	U.S.	Hi SDI		
	2020						13.62			45,222	329,484,123	firearm	Pop	Pop		
	2021						14.65			48,830	331,893,745	deaths	Million	Millions	Pop	Deaths
**Firearm**	1990	2.23	2.26	2.2	19,167	39%	6.34	6.43	6.24	17,570		46%	277.1	859.5	582.4	1,597
**Homicides**	1991	2.34	2.36	2.31	20,105	41%	6.66	6.74	6.58	16,739		47%	251.3	859.2	607.8	3,366
	1992	2.34	2.36	2.31	16,388	41%	6.6	6.68	6.52	17,457		48%	264.5	700.3	435.8	-1,070
	1993	2.41	2.43	2.38	20,093	42%	6.78	6.87	6.7	17,371		48%	256.2	833.7	577.5	2,722
	1994	2.36	2.39	2.34	20,685	42%	6.61	6.69	6.54	12,886		48%	194.9	876.5	681.6	7,799
	1995	2.2	2.23	2.18	15,038	41%	6.11	6.18	6.04	13,176		46%	215.7	683.5	467.9	1,861
	1996	2.02	2.05	2	20,348	40%	5.55	5.62	5.49	14,152		45%	255.0	1007.3	752.4	6,196
	1997	1.89	1.91	1.87	15,865	39%	5.15	5.22	5.09	17,862		44%	346.8	839.4	492.6	-1,998
	1998	1.76	1.78	1.73	15,327	38%	4.76	4.82	4.68	16,162		43%	339.5	870.9	531.3	-835
	1999	1.68	1.7	1.66	17,495	38%	4.53	4.58	4.48	14,786		44%	326.4	1041.4	715.0	2,709
	2000	1.65	1.66	1.63	18,987	38%	4.46	4.51	4.41	12,445		44%	279.0	1150.7	871.7	6,543
	2001	1.7	1.72	1.68	14,723	39%	4.6	4.65	4.55	13,823		45%	300.5	866.1	565.6	900
	2002	1.73	1.75	1.72	15,044	40%	4.7	4.75	4.65	12,401		45%	263.9	869.6	605.8	2,643
	2003	1.73	1.74	1.71	14,536	41%	4.68	4.74	4.61	12,316		45%	263.2	840.2	577.1	2,220
	2004	1.7	1.72	1.68	15,288	41%	4.61	4.67	4.55	12,032		46%	261.0	899.3	638.3	3,256
	2005	1.77	1.79	1.75	15,406	42%	4.8	4.86	4.73	13,207		47%	275.2	870.4	595.3	2,199
	2006	1.79	1.82	1.77	14,276	43%	4.89	4.97	4.82	12,785		48%	261.4	797.6	536.1	1,492
	2007	1.75	1.77	1.72	16,163	42%	4.76	4.83	4.7	13,158		46%	276.4	923.6	647.1	3,004
	2008	1.68	1.7	1.66	15,383	41%	4.54	4.6	4.47	13,320		44%	293.4	915.6	622.2	2,062
	2009	1.62	1.64	1.59	14,583	40%	4.31	4.37	4.24	13,770		43%	319.5	900.2	580.7	813
	2010	1.56	1.58	1.54	15,876	40%	4.14	4.19	4.08	12,863		41%	310.7	1017.7	707.0	3,013
	2011	1.55	1.57	1.54	15,890	40%	4.1	4.15	4.05	13,903		40%	339.1	1025.1	686.0	1,987
	2012	1.58	1.6	1.56	15,438	40%	4.19	4.26	4.11	12,454		40%	297.2	977.1	679.8	2,984
	2013	1.49	1.52	1.47	14,647	39%	3.95	4.01	3.88	13,385		38%	338.9	983.0	644.2	1,262
	2014	1.46	1.49	1.43	15,026	38%	3.88	3.96	3.79	12,845		38%	331.1	1029.2	698.1	2,181
	2015	1.61	1.64	1.58	14,010	40%	4.36	4.45	4.26	13,575		39%	311.3	870.2	558.8	435
	2016	1.71	1.76	1.65	15,222	41%	4.65	4.81	4.47	13,001		39%	279.6	890.2	610.6	2,221
	2017	1.66	1.7	1.56	15,476	41%	4.47	4.58	4.15	12,200		37%	272.9	932.3	659.4	3,276
	2018	1.58	1.63	1.49	16,433	40%	4.44	4.37	3.96	13,958	327,167,434	37%	314.4	1040.1	725.7	2,475
	2019	1.55	1.6	1.45	14,878	40%	4.59	4.25	3.83	14,414	328,239,523	39%	314.0	959.9	645.9	464
	2020						6.19			19,384	329,484,123	45%				
	2021						6.66			20,958	331,893,745	45%				
**Firearm**	1990	3.12	3.54	2.83	28,963	55%	6.86	7.29	6.23	19,290		50%	281.2	928.3	647.1	9,672
**Suicides**	1991	3.08	3.49	2.8	28,879	54%	6.78	7.22	6.18	19,318		48%	284.9	937.6	652.7	9,561
	1992	3.02	3.44	2.74	27,024	53%	6.66	7.1	6.06	19,702		48%	295.8	894.8	599.0	7,322
	1993	3.04	3.47	2.76	29,074	53%	6.77	7.25	6.16	19,170		48%	283.2	956.4	673.2	9,904
	1994	2.99	3.4	2.73	28,598	52%	6.7	7.17	6.1	18,572		48%	277.2	956.5	679.3	10,026
	1995	2.92	3.33	2.66	25,716	51%	6.59	7.08	6.01	18,882		50%	286.5	880.7	594.2	6,835
	1996	2.8	3.2	2.54	28,870	49%	6.36	6.83	5.79	19,622		51%	308.5	1031.1	722.5	9,248
	1997	2.7	3.1	2.44	25,245	47%	6.16	6.66	5.6	19,701		52%	319.8	935.0	615.2	5,545
	1998	2.61	2.99	2.35	26,528	46%	6	6.5	5.43	19,615		54%	326.9	1016.4	689.5	6,913

	1999	2.54	2.87	2.27	27,674	44%	5.97	6.33	5.27	16,599	279,040,168	58%	278.0	1089.5	811.5	11,075
	2000	2.48	2.78	2.2	28,511	43%	5.91	6.24	5.19	16,586	281,421,906	58%	280.6	1149.6	869.0	11,925
	2001	2.44	2.74	2.16	26,047	43%	5.91	6.23	5.17	16,869	284,968,955	57%	285.4	1067.5	782.1	9,178
	2002	2.4	2.7	2.14	26,499	42%	5.93	6.23	5.2	17,108	287,625,193	57%	288.5	1104.1	815.6	9,391
	2003	2.36	2.65	2.09	25,788	41%	5.78	6.17	5.12	16,907	290,107,933	56%	292.5	1092.7	800.2	8,881
	2004	2.26	2.55	2.01	24,854	40%	5.62	6.03	5	16,750	292,805,298	56%	298.0	1099.7	801.7	8,104
	2005	2.27	2.56	2.02	25,717	40%	5.67	6.14	5.09	17,002	295,516,599	55%	299.9	1132.9	833.1	8,715
	2006	2.26	2.55	2.02	27,396	40%	5.55	6.23	5.19	16,883	298,379,912	54%	304.2	1212.2	908.0	10,513
	2007	2.24	2.55	2.02	25,442	39%	5.61	6.29	5.22	17,352	301,231,207	55%	309.3	1135.8	826.5	8,090
	2008	2.25	2.56	2.04	25,557	39%	5.81	6.37	5.3	18,223	304,093,966	57%	313.6	1135.9	822.2	7,334
	2009	2.24	2.58	2.04	26,554	39%	5.92	6.43	5.34	18,735	306,771,529	59%	316.5	1185.4	869.0	7,819
	2010	2.22	2.57	2.03	29,027	39%	6.08	6.44	5.35	19,392	308,745,538	60%	318.9	1307.5	988.6	9,635
	2011	2.23	2.6	2.04	25,648	39%	6.2	6.56	5.44	19,990	311,591,917	61%	322.4	1150.2	827.7	5,658
	2012	2.22	2.58	2.03	26,120	39%	6.31	6.59	5.46	20,666	313,914,040	60%	327.5	1176.6	849.0	5,454
	2013	2.21	2.58	2.03	26,977	39%	6.41	6.62	5.48	21,175	316,128,839	61%	330.3	1220.7	890.3	5,802
	2014	2.21	2.58	2.04	27,174	39%	6.37	6.7	5.55	21,386	318,857,056	62%	335.7	1229.6	893.9	5,788
	2015	2.27	2.64	2.1	27,655	40%	6.51	6.87	5.71	22,018	321,418,820	59%	338.2	1218.3	880.0	5,637
	2016	2.33	2.69	2.16	28,748	41%	6.75	7.08	5.93	22,938	323,127,513	57%	339.8	1233.8	894.0	5,810
	2017	2.28	2.62	2.1	28,516	40%	6.93	6.86	5.78	23,854	325,719,178	58%	344.2	1250.7	906.5	4,662
	2018	2.22	2.56	2.04	29,365	39%	7.04	6.69	5.62	24,432	327,167,434	59%	347.0	1322.8	975.7	4,933
	2019	2.16	2.48	1.99	28,386	38%	6.84	6.5	5.42	23,941	328,239,523	58%	350.0	1314.2	964.2	4,445
	2020						6.95			24,292	329,484,123	51%				
	2021						7.53			26,328	331,893,745	51%				
**Unintentional**	1990	0.31	0.36	0.24	2,593	5%	0.65	0.68	0.48	1,589		5%	244.5	836.4	591.9	1,004
**FA Deaths**	1991	0.3	0.34	0.23	2,529	5%	0.63	0.66	0.47	1,447		4%	229.6	843.1	613.5	1,083
	1992	0.29	0.33	0.22	2,339	5%	0.6	0.63	0.45	1,630		4%	271.7	806.5	534.8	709
	1993	0.28	0.32	0.22	2,445	5%	0.59	0.62	0.45	1,098		4%	186.1	873.3	687.2	1,347
	1994	0.27	0.31	0.21	1,925	5%	0.56	0.59	0.43	948		4%	169.4	713.0	543.6	977
	1995	0.26	0.29	0.2	2,412	5%	0.53	0.56	0.41	1,528		4%	288.2	927.8	639.6	885
	1996	0.25	0.27	0.19	1,741	4%	0.48	0.52	0.38	860		4%	179.1	696.3	517.2	881
	1997	0.23	0.25	0.18	2,012	4%	0.44	0.48	0.35	1,512		4%	343.7	874.9	531.3	500
	1998	0.22	0.24	0.17	2,251	4%	0.4	0.45	0.33	1,374		4%	343.6	1023.0	679.4	876
	1999	0.21	0.23	0.17	1,589	4%	0.3	0.43	0.31	824	279,040,168	3%	274.7	756.6	481.9	765
	2000	0.2	0.22	0.16	2,130	3%	0.26	0.41	0.29	776	281,421,906	3%	298.5	1065.2	766.7	1,354
	2001	0.19	0.21	0.15	1,862	3%	0.28	0.39	0.28	802	284,968,955	3%	286.4	980.1	693.7	1,060
	2002	0.19	0.21	0.15	1,791	3%	0.27	0.38	0.28	762	287,625,193	3%	282.2	942.5	660.2	1,029
	2003	0.18	0.2	0.15	1,447	3%	0.25	0.37	0.26	730	290,107,933	2%	292.0	803.7	511.7	717
	2004	0.17	0.2	0.14	1,765	3%	0.22	0.36	0.25	649	292,805,298	2%	295.0	1038.2	743.2	1,116
	2005	0.17	0.19	0.14	1,636	3%	0.27	0.35	0.25	789	295,516,599	3%	292.2	962.6	670.4	847
	2006	0.16	0.19	0.14	1,378	3%	0.24	0.34	0.24	642	298,379,912	2%	267.5	861.1	593.6	736
	2007	0.16	0.19	0.14	1,698	3%	0.2	0.33	0.23	613	301,231,207	2%	306.5	1061.5	755.0	1,085
	2008	0.15	0.18	0.13	1,631	3%	0.19	0.31	0.22	592	304,093,966	2%	311.6	1087.2	775.7	1,039
	2009	0.15	0.18	0.13	1,550	3%	0.18	0.29	0.21	554	306,771,529	2%	307.8	1033.1	725.3	996
	2010	0.14	0.17	0.12	1,360	2%	0.2	0.28	0.2	606	308,745,538	2%	303.0	971.1	668.1	754
	2011	0.14	0.17	0.12	1,427	2%	0.2	0.27	0.2	591	311,591,917	2%	295.5	1019.5	724.0	836
	2012	0.14	0.16	0.12	1,510	2%	0.19	0.26	0.19	548	313,914,040	2%	288.4	1078.8	790.4	962
	2013	0.13	0.16	0.11	1,471	2%	0.16	0.26	0.18	505	316,128,839	2%	315.6	1131.2	815.6	966
	2014	0.13	0.16	0.11	1,399	2%	0.14	0.26	0.18	461	318,857,056	1%	329.3	1076.2	746.9	938
	2015	0.13	0.16	0.11	1,377	2%	0.15	0.26	0.18	489	321,418,820	1%	326.0	1058.9	732.9	888
	2016	0.13	0.16	0.11	1,373	2%	0.17	0.26	0.18	495	323,127,513	1%	291.2	1056.2	765.1	878
	2017	0.13	0.16	0.11	1,371	2%	0.16	0.26	0.18	486	325,719,178	1%	303.8	1055.0	751.2	885
	2018	0.13	0.16	0.1	1,375	2%	0.14	0.25	0.17	458	327,167,434	1%	327.1	1057.8	730.6	917
	2019	0.13	0.16	0.1	1,348	2%	0.16	0.24	0.17	486	328,239,523	1%	303.8	1037.1	733.4	862
	2020						0.17			535	329,484,123	1%				
	2021						0.17			549	331,893,745	1%				

	Australia	Canada	New Zealand	Switzerland	Israel
**Crude Rate**	Mean	Mean	Mean	Mean	Mean
1.94	3.44	4.88	2.78	8.06	3.05
2.20	3.25	4.86	2.56	8.34	3.10
1.11	3.11	4.64	2.67	8.18	3.23
2.14	2.83	4.45	2.41	7.83	3.31
2.80	2.74	4.24	2.28	7.53	3.44
1.69	2.59	3.93	2.29	6.49	3.46
2.27	2.55	3.77	2.10	6.14	3.65
0.59	2.26	3.55	2.02	6.10	3.80
0.97	2.04	3.32	1.76	5.74	3.51
1.90	1.84	3.18	1.60	5.33	3.77
2.30	1.70	2.93	1.53	5.12	4.43
1.49	1.51	2.79	1.52	4.99	4.27
1.74	1.39	2.60	1.46	4.72	4.30
1.63	1.31	2.49	1.36	4.35	4.04
1.67	1.12	2.40	1.27	4.08	4.09
1.54	1.04	2.45	1.29	3.73	3.89
1.56	1.05	2.32	1.22	3.40	3.41
1.59	1.00	2.25	1.18	3.21	2.49
1.36	1.00	2.25	1.17	2.98	2.97
1.18	0.98	2.13	1.18	2.93	2.87
1.51	0.95	2.16	1.04	2.60	2.87
1.09	0.87	1.99	0.96	2.46	2.73
1.20	0.85	2.07	0.97	2.34	2.47
0.97	0.85	1.97	1.00	2.29	2.33
1.09	0.86	2.03	0.95	2.08	2.19
0.84	0.85	2.10	0.94	2.07	2.30
1.13	0.84	2.17	0.95	1.98	2.17
1.13	0.80	2.12	0.93	1.98	2.15
0.97	0.80	2.06	0.93	1.98	2.16
0.65	0.80	2.02	0.92	1.91	2.16

	Mean	HiCI	LoCI	Mean	HiCI	LoCI	Mean	HiCI	LoCI	Mean	HiCI	LoCI	Mean	HiCI	LoCI
**0.27419**	0.53	0.56	0.5	0.72	0.76	0.68	0.56	0.59	0.53	0.67	0.72	0.62	0.92	0.99	0.85
**0.55369**	0.54	0.57	0.51	0.81	0.85	0.77	0.39	0.41	0.37	0.67	0.72	0.63	0.83	0.89	0.78
**-0.24543**	0.52	0.55	0.49	0.73	0.77	0.69	0.44	0.46	0.41	0.72	0.77	0.67	0.82	0.88	0.76
**0.47136**	0.44	0.47	0.42	0.63	0.66	0.59	0.33	0.35	0.31	0.79	0.84	0.74	0.99	1.07	0.92
**1.14436**	0.42	0.44	0.39	0.65	0.68	0.62	0.38	0.4	0.36	0.69	0.74	0.64	1.02	1.09	0.94
**0.3978**	0.41	0.44	0.39	0.52	0.55	0.5	0.33	0.35	0.31	0.49	0.52	0.46	0.9	0.96	0.83
**0.8236**	0.51	0.54	0.48	0.57	0.6	0.55	0.3	0.32	0.28	0.6	0.64	0.57	0.99	1.06	0.92
**-0.40555**	0.42	0.44	0.39	0.51	0.53	0.48	0.31	0.33	0.3	0.73	0.77	0.69	0.97	1.04	0.9
**-0.15716**	0.35	0.37	0.33	0.47	0.49	0.44	0.17	0.18	0.16	0.49	0.52	0.46	0.73	0.79	0.68
**0.37895**	0.31	0.33	0.29	0.48	0.51	0.46	0.2	0.21	0.19	0.51	0.54	0.48	1.13	1.2	1.05
**0.75057**	0.32	0.34	0.3	0.48	0.51	0.45	0.25	0.27	0.24	0.35	0.37	0.33	1.67	1.78	1.58
**0.15916**	0.26	0.28	0.24	0.49	0.52	0.46	0.24	0.26	0.23	0.53	0.57	0.5	1.49	1.6	1.37
**0.43635**	0.25	0.26	0.23	0.46	0.49	0.44	0.23	0.25	0.22	0.46	0.49	0.43	1.6	1.7	1.47
**0.38474**	0.24	0.25	0.22	0.45	0.48	0.43	0.2	0.21	0.19	0.37	0.39	0.35	1.49	1.58	1.39
**0.51015**	0.14	0.15	0.13	0.5	0.53	0.47	0.17	0.19	0.17	0.44	0.47	0.42	1.81	1.9	1.72
**0.36939**	0.13	0.14	0.12	0.62	0.65	0.59	0.19	0.2	0.18	0.35	0.37	0.33	1.67	1.74	1.59
**0.27825**	0.19	0.21	0.17	0.6	0.63	0.56	0.18	0.19	0.17	0.26	0.27	0.24	1.42	1.49	1.35
**0.46423**	0.16	0.18	0.15	0.56	0.59	0.53	0.15	0.16	0.14	0.24	0.25	0.22	0.59	0.62	0.56
**0.33145**	0.16	0.18	0.15	0.63	0.67	0.59	0.19	0.2	0.18	0.22	0.24	0.21	1.17	1.23	1.11
**0.14007**	0.19	0.2	0.17	0.55	0.58	0.52	0.22	0.24	0.21	0.26	0.27	0.24	1.22	1.28	1.16
**0.42614**	0.19	0.21	0.18	0.6	0.63	0.56	0.15	0.16	0.15	0.2	0.21	0.18	1.31	1.37	1.23
**0.28959**	0.17	0.18	0.15	0.51	0.54	0.48	0.1	0.1	0.09	0.21	0.22	0.2	1.3	1.36	1.23
**0.43891**	0.18	0.2	0.17	0.56	0.6	0.53	0.1	0.1	0.09	0.21	0.22	0.2	1.16	1.22	1.1
**0.19595**	0.18	0.19	0.17	0.47	0.5	0.44	0.18	0.19	0.17	0.2	0.22	0.19	1.1	1.16	1.05
**0.31243**	0.18	0.19	0.17	0.48	0.51	0.44	0.14	0.15	0.13	0.14	0.15	0.13	1.04	1.1	0.98
**0.07791**	0.15	0.16	0.14	0.51	0.55	0.47	0.17	0.18	0.16	0.19	0.21	0.18	1.13	1.19	1.07
**0.36375**	0.19	0.21	0.18	0.55	0.6	0.49	0.16	0.17	0.15	0.15	0.17	0.14	1.04	1.11	0.97
**0.4969**	0.18	0.19	0.17	0.52	0.57	0.47	0.15	0.16	0.14	0.17	0.19	0.16	1.04	1.12	0.97
**0.34104**	0.18	0.19	0.16	0.5	0.55	0.45	0.15	0.16	0.14	0.18	0.2	0.17	1.05	1.13	0.97
**0.07187**	0.18	0.19	0.16	0.5	0.55	0.45	0.15	0.16	0.14	0.18	0.2	0.17	1.05	1.15	0.96

**1.49475**	2.73	3.22	2.29	3.92	4.59	3.39	2	2.53	1.74	7.06	8.15	5.9	1.77	2.19	1.56
**1.46487**	2.55	3.09	2.16	3.82	4.55	3.33	1.96	2.55	1.73	7.35	8.46	6.1	1.93	2.37	1.72
**1.22234**	2.44	2.98	2.08	3.69	4.47	3.26	2.03	2.65	1.81	7.16	8.28	6.01	2.08	2.57	1.86
**1.47111**	2.25	2.8	1.94	3.62	4.46	3.24	1.91	2.55	1.71	6.78	7.8	5.8	2.03	2.52	1.81
**1.47603**	2.18	2.78	1.9	3.41	4.25	3.09	1.75	2.4	1.55	6.6	7.65	5.67	2.14	2.69	1.93
**1.15029**	2.04	2.66	1.8	3.23	4.08	2.96	1.82	2.56	1.6	5.8	6.86	5.1	2.28	2.87	2.05
**1.27992**	1.9	2.55	1.72	3.03	3.88	2.78	1.67	2.41	1.48	5.37	6.34	4.74	2.36	3.05	2.14
**0.90129**	1.7	2.34	1.54	2.88	3.71	2.63	1.61	2.38	1.42	5.22	6.1	4.6	2.5	3.28	2.25
**1.00263**	1.54	2.15	1.4	2.71	3.5	2.46	1.49	2.2	1.32	5.1	5.95	4.49	2.42	3.1	2.2

**1.36476**	1.38	1.92	1.25	2.57	3.32	2.32	1.32	1.93	1.17	4.7	5.42	4.1	2.31	2.88	2.09
**1.37227**	1.22	1.66	1.1	2.34	2.99	2.08	1.19	1.71	1.06	4.65	5.34	4.03	2.43	2.92	2.17
**1.17355**	1.11	1.49	0.99	2.19	2.8	1.93	1.2	1.7	1.07	4.35	5.01	3.72	2.47	2.88	2.12
**1.15139**	1	1.33	0.87	2.04	2.64	1.78	1.15	1.6	1.03	4.16	4.81	3.52	2.4	2.7	1.98
**1.10986**	0.93	1.21	0.79	1.95	2.53	1.68	1.07	1.49	0.96	3.88	4.45	3.24	2.27	2.53	1.85
**1.01085**	0.85	1.11	0.72	1.81	2.33	1.55	1.02	1.42	0.9	3.55	4.1	2.97	2.03	2.3	1.65
**1.04617**	0.8	1.05	0.67	1.75	2.25	1.48	1.03	1.43	0.92	3.3	3.84	2.73	1.97	2.23	1.61
**1.15778**	0.77	1.01	0.64	1.65	2.12	1.39	0.97	1.35	0.85	3.07	3.55	2.51	1.77	1.98	1.44
**0.97885**	0.77	1.03	0.65	1.62	2.09	1.36	0.96	1.32	0.85	2.9	3.32	2.35	1.69	1.92	1.39
**0.892**	0.77	1.04	0.64	1.55	2.03	1.31	0.91	1.26	0.79	2.7	3.1	2.14	1.62	1.87	1.32
**0.89977**	0.73	0.99	0.62	1.52	2.05	1.29	0.89	1.23	0.77	2.62	2.98	2.04	1.49	1.75	1.22
**0.97464**	0.7	0.95	0.6	1.5	2	1.27	0.83	1.18	0.73	2.35	2.69	1.81	1.42	1.69	1.17
**0.68361**	0.65	0.91	0.55	1.42	1.91	1.2	0.8	1.13	0.7	2.2	2.52	1.66	1.3	1.54	1.04
**0.64231**	0.63	0.89	0.54	1.46	1.96	1.24	0.81	1.13	0.7	2.09	2.42	1.57	1.2	1.43	0.95
**0.65168**	0.63	0.9	0.54	1.45	1.97	1.25	0.77	1.05	0.66	2.05	2.38	1.54	1.12	1.31	0.87
**0.64751**	0.64	0.93	0.56	1.5	2.03	1.29	0.75	1.04	0.64	1.9	2.2	1.42	1.05	1.23	0.8
**0.64048**	0.66	0.97	0.58	1.54	2.08	1.3	0.71	0.99	0.59	1.85	2.12	1.36	1.07	1.24	0.8
**0.64988**	0.61	0.89	0.53	1.57	2.09	1.33	0.73	1	0.62	1.79	2.06	1.31	1.03	1.19	0.76
**0.51427**	0.58	0.83	0.5	1.55	2.04	1.32	0.72	1	0.6	1.77	2.05	1.3	1.01	1.18	0.76
**0.5056**	0.58	0.83	0.49	1.51	1.97	1.28	0.72	1	0.6	1.76	2.05	1.28	1.01	1.18	0.75
**0.46103**	0.58	0.83	0.49	1.47	1.9	1.24	0.71	0.99	0.6	1.69	1.99	1.23	1.01	1.18	0.74
**0.16957**	0.18	0.21	0.12	0.24	0.27	0.13	0.22	0.26	0.13	0.33	0.4	0.18	0.36	0.41	0.29
**0.17647**	0.16	0.19	0.11	0.23	0.26	0.12	0.21	0.25	0.13	0.32	0.39	0.18	0.34	0.38	0.27
**0.13253**	0.15	0.18	0.11	0.22	0.24	0.12	0.2	0.23	0.12	0.3	0.36	0.16	0.33	0.37	0.26
**0.19605**	0.14	0.17	0.1	0.2	0.22	0.11	0.17	0.2	0.12	0.26	0.32	0.15	0.29	0.33	0.23
**0.17964**	0.14	0.17	0.1	0.18	0.21	0.11	0.15	0.18	0.11	0.24	0.29	0.14	0.28	0.31	0.23
**0.13831**	0.14	0.16	0.1	0.18	0.2	0.11	0.14	0.19	0.11	0.2	0.24	0.11	0.28	0.31	0.23
**0.17037**	0.14	0.17	0.1	0.17	0.19	0.1	0.13	0.18	0.1	0.17	0.2	0.1	0.3	0.34	0.24
**0.09415**	0.14	0.16	0.1	0.16	0.18	0.1	0.1	0.17	0.09	0.15	0.18	0.09	0.33	0.38	0.26
**0.12897**	0.15	0.17	0.1	0.14	0.16	0.09	0.1	0.16	0.09	0.15	0.17	0.08	0.36	0.4	0.26
**0.15871**	0.15	0.17	0.1	0.13	0.15	0.09	0.08	0.14	0.06	0.12	0.14	0.07	0.33	0.37	0.25
**0.17664**	0.16	0.18	0.09	0.11	0.13	0.08	0.09	0.14	0.07	0.12	0.14	0.07	0.33	0.37	0.24
**0.15284**	0.14	0.16	0.09	0.11	0.12	0.08	0.08	0.13	0.07	0.11	0.12	0.06	0.31	0.35	0.23
**0.1558**	0.14	0.16	0.08	0.1	0.12	0.07	0.08	0.13	0.07	0.1	0.11	0.06	0.3	0.35	0.23
**0.14006**	0.14	0.16	0.08	0.09	0.11	0.07	0.09	0.13	0.07	0.1	0.11	0.06	0.28	0.32	0.22
**0.15015**	0.13	0.15	0.08	0.09	0.11	0.07	0.08	0.12	0.06	0.09	0.1	0.06	0.25	0.28	0.2
**0.12641**	0.11	0.13	0.07	0.08	0.11	0.06	0.07	0.11	0.06	0.08	0.09	0.05	0.25	0.27	0.19
**0.12395**	0.09	0.1	0.06	0.07	0.1	0.06	0.07	0.11	0.06	0.07	0.08	0.05	0.22	0.24	0.17
**0.14376**	0.07	0.09	0.06	0.07	0.1	0.06	0.07	0.1	0.06	0.07	0.08	0.05	0.21	0.23	0.16
**0.13393**	0.07	0.09	0.06	0.07	0.09	0.06	0.07	0.1	0.06	0.06	0.07	0.04	0.18	0.2	0.15
**0.13727**	0.06	0.09	0.05	0.06	0.09	0.05	0.07	0.1	0.06	0.05	0.07	0.04	0.16	0.18	0.13
**0.11279**	0.06	0.08	0.05	0.06	0.08	0.05	0.06	0.09	0.05	0.05	0.06	0.04	0.14	0.17	0.12
**0.11551**	0.05	0.08	0.04	0.06	0.08	0.05	0.06	0.09	0.05	0.05	0.06	0.04	0.13	0.15	0.11
**0.12175**	0.04	0.07	0.04	0.05	0.08	0.05	0.06	0.09	0.05	0.04	0.06	0.04	0.11	0.14	0.1
**0.11839**	0.04	0.07	0.03	0.05	0.08	0.04	0.05	0.08	0.04	0.04	0.06	0.04	0.11	0.13	0.09
**0.12559**	0.04	0.07	0.03	0.05	0.08	0.04	0.06	0.08	0.05	0.04	0.05	0.03	0.1	0.13	0.09
**0.1211**	0.04	0.07	0.03	0.05	0.08	0.04	0.06	0.08	0.05	0.03	0.05	0.03	0.1	0.13	0.09
**0.11478**	0.04	0.07	0.03	0.05	0.08	0.04	0.06	0.08	0.05	0.04	0.05	0.03	0.1	0.12	0.09
**0.11787**	0.04	0.07	0.03	0.05	0.08	0.04	0.06	0.08	0.05	0.04	0.05	0.03	0.1	0.12	0.09
**0.12552**	0.04	0.06	0.03	0.05	0.08	0.04	0.06	0.08	0.05	0.04	0.05	0.03	0.1	0.12	0.09
**0.11757**	0.04	0.06	0.03	0.05	0.08	0.04	0.06	0.08	0.05	0.04	0.05	0.03	0.1	0.12	0.08
