# Supplementary material for: Fallacy of attributing the U.S. firearm mortality epidemic to mental health
Source: PLoS One. 2024 Aug 5;19(8):e0290138. doi: 10.1371/journal.pone.0290138 (PMC11299823; doi:10.1371/journal.pone.0290138)
Supplement: S4 File — (PDF) [file pone.0290138.s006.pdf]

		Hi SDI Countries	N =	41		13 th of 9 categories								
2019		U.S. Rank	USA	Andorra	Australia	Austria	Belgium	Bermuda	Brunei	Canada	Cyprus	Czechia	Denmark	England	Estonia
Mean	Mental disorders	5	15,654.9	14,064.0	17,563.2	14,433.7	13,602.0	13,696.7	9,562.2	13,534.6	14,475.5	10,324.5	12,749.2	13,834.3	11,285.9
	Anxiety	12	5,697.8	5,453.4	5,815.2	6,006.4	5,047.2	4,329.2	2,856.9	4,320.6	6,467.2	3,153.9	4,873.9	4,425.2	3,378.8
	Depression	2	4,376.0	3,607.2	4,382.5	3,125.2	3,542.0	3,177.6	1,603.1	3,290.5	3,112.7	2,878.9	3,482.9	4,105.7	3,693.6
	ADHD **	4	2,102.0	1,296.8	3,377.4	1,632.4	1,290.1	3,059.9	1,470.9	2,037.7	1,294.8	1,061.8	918.1	1,308.0	1,061.8
	Bipolar	32	601.3	934.8	1,125.1	900.4	900.3	977.4	606.5	805.3	910.4	547.2	940.3	1,121.4	522.8
	Conduct	35	554.1	628.2	612.5	610.0	626.6	559.1	531.9	500.5	629.0	584.0	627.0	685.8	584.1
	Schizophrenia	1	433.7	286.5	388.7	276.8	274.2	305.3	304.1	288.6	274.0	296.6	241.4	244.2	290.1
	Autism	4	643.4	580.4	435.9	567.5	563.3	350.4	569.6	608.1	562.3	379.7	429.0	773.0	404.1
	Other	3	1,792.2	1,611.4	1,911.3	1,600.4	1,599.1	1,458.7	1,611.8	1,797.6	1,594.7	1,465.5	1,602.4	1,458.8	1,457.7
	Eating	21	424.4	520.9	1,031.7	562.2	436.8	367.4	424.8	428.6	408.1	192.3	471.3	404.63	175.2
	Idiopathic developmental intell	9	428.3	254.2	312.9	324.9	345.6	148.3	93.8	497.6	413.0	340.7	74.5	373.62	386.5
Hi CI	Mental disorders		16,908.2	15,440.5	19,103.9	15,861.3	14,876.4	15,114.1	10,556.5	14,895.9	16,016.3	11,264.6	14,015.7	14,819.5	12,301.9
	Anxiety disorders		6,728.4	6,872.0	7,271.5	7,419.0	6,351.2	5,414.1	3,558.2	5,477.1	8,099.4	3,908.8	6,067.9	5,144.9	4,259.5
	Depressive disorders		4,843.1	4,175.1	5,062.4	3,587.0	4,054.3	3,662.3	1,855.3	3,815.7	3,618.8	3,332.8	3,979.9	4,617.2	4,209.0
	Attention-deficit/hyperactivity disorder		2,853.5	1,770.3	4,247.8	2,234.4	1,762.3	4,107.1	2,051.6	2,770.0	1,770.3	1,424.4	1,227.8	1,832.4	1,425.3
	Bipolar disorder		638.3	1,164.9	1,305.6	1,121.1	1,125.2	1,240.3	752.0	936.1	1,143.9	690.8	1,181.1	1,309.5	656.7
	Conduct disorder		726.4	812.4	775.0	789.2	809.6	722.9	704.5	662.2	812.5	763.8	810.0	876.4	763.8
	Schizophrenia		500.8	348.9	420.4	339.6	334.3	372.7	371.8	292.4	330.7	362.6	294.8	281.5	354.4
	Autism spectrum disorders		763.6	699.5	523.7	675.3	677.1	422.9		696.6	672.0	460.6	444.9	914.9	485.6
	Other mental disorders		2,246.3	2,075.0	2,256.7	2,061.5	2,059.4	1,866.0	2,075.1	2,267.8	2,053.7	1,873.4	2,064.0	1,849.2	1,864.7
	Eating disorders		540.4	669.3	1,225.0	716.7	550.1	476.7	535.3	548.3	514.8	250.1	598.0	511.7	225.4
	Idiopathic developmental intellectual di		736.2	467.2	539.6	576.9	602.9	276.5	225.7	839.5	703.1	565.2	175.3	651.3	641.9
Lo CI	Mental disorders		14,618.6	12,905.0	16,274.1	13,210.7	12,470.2	12,497.7	8,715.5	12,390.2	13,129.0	9,487.7	11,683.0	12,953.0	10,395.5
	Anxiety disorders		4,843.3	4,341.1	4,639.8	4,815.2	4,033.7	3,459.8	2,262.1	3,442.7	5,125.8	2,534.5	3,836.1	3,796.2	2,719.2
	Depressive disorders		3,958.5	3,122.0	3,817.7	2,737.5	3,110.2	2,783.1	1,384.1	2,894.2	2,696.6	2,531.4	3,053.4	3,643.2	3,256.8
	Attention-deficit/hyperactivity disorder		1,509.4	942.8	2,585.8	1,187.7	938.2	2,243.7	1,052.3	1,465.3	940.6	761.4	650.9	931.1	761.1
	Bipolar disorder		565.4	722.0	946.8	697.0	690.2	743.3	468.4	668.0	702.0	419.7	716.6	938.8	407.3
	Conduct disorder		389.2	457.8	483.2	448.7	456.5	405.5	384.7	353.0	458.6	420.0	456.8	509.6	420.1
	Schizophrenia		372.4	229.7	359.8	220.6	219.8	242.2	242.7	285.0	219.2	237.7	189.7	210.0	231.4
	Autism spectrum disorders		535.1	476.1	362.9	468.3	464.8	284.5	469.3	526.8	465.8	308.4	417.9	642.6	331.8
	Other mental disorders		1,372.3	1,243.7	1,584.4	1,234.9	1,233.9	1,130.5	1,243.9	1,363.2	1,230.4	1,135.8	1,236.6	1,117.9	1,129.6
	Eating disorders		316.4	394.8	849.9	429.9	334.6	269.3	323.4	316.8	311.6	139.7	361.4	306.5	128.8
	Idiopathic developmental intellectual di		133.7	59.5	98.3	87.9	95.1	30.2	4.7	165.2	130.0	116.5	7.9	110.5	134.5
Hi CI	Mental disorders		1,253.3	1,376.5	1,540.7	1,427.7	1,274.4	1,417.5	994.3	1,361.3	1,540.9	940.1	1,266.4	985.1	1,016.0
Diff	Anxiety disorders		1,030.7	1,418.6	1,456.3	1,412.6	1,304.0	1,084.8	701.3	1,156.5	1,632.2	754.9	1,194.0	719.7	880.7
	Depressive disorders		467.1	567.9	680.0	461.8	512.3	484.8	252.1	525.2	506.1	454.0	497.0	511.5	515.3
	Attention-deficit/hyperactivity disorder		751.5	473.5	870.4	602.0	472.3	1,047.3	580.7	732.3	475.6	362.6	309.6	524.4	363.5
	Bipolar disorder		37.0	230.1	180.5	220.6	224.9	263.0	145.5	130.9	233.5	143.7	240.8	188.2	134.0
	Conduct disorder		172.3	184.2	162.5	179.2	183.0	163.8	172.5	161.7	183.6	179.8	183.0	190.6	179.8
	Schizophrenia		67.1	62.4	31.8	62.9	60.1	67.3	67.7	3.8	56.7	66.0	53.5	37.2	64.3
	Autism spectrum disorders		120.2	119.1	87.9	107.7	113.8	72.4	#VALUE!	88.5	109.7	81.0	16.0	141.9	81.5
	Other mental disorders		454.2	463.5	345.4	461.1	460.4	407.3	463.3	470.2	459.0	407.9	461.6	390.4	407.0
	Eating disorders		116.0	148.4	193.3	154.5	113.2	109.3	110.6	119.7	106.7	57.9	126.8	107.1	50.2
	Idiopathic developmental intellectual di		307.9	213.0	226.7	252.0	257.4	128.2	131.9	341.9	290.1	224.5	100.9	277.7	255.4
Lo CI	Mental disorders		1,036.3	1,159.0	1,289.2	1,223.0	1,131.8	1,199.0	846.7	1,144.3	1,346.5	836.8	1,066.2	881.3	890.4
Diff	Anxiety disorders		854.4	1,112.3	1,175.4	1,191.2	1,013.5	869.4	594.8	878.0	1,341.4	619.4	1,037.8	629.0	659.6
	Depressive disorders		417.5	485.2	564.8	387.7	431.8	394.5	219.0	396.3	416.1	347.5	429.5	462.6	436.9
	Attention-deficit/hyperactivity disorder		592.6	354.0	791.6	444.7	351.8	816.1	418.6	572.4	354.2	300.5	267.2	376.9	300.7
	Bipolar disorder		35.9	212.8	178.3	203.5	210.1	234.0	138.0	137.3	208.4	127.5	223.7	182.6	115.5
	Conduct disorder		164.9	170.3	129.3	161.3	170.2	153.6	147.2	147.5	170.4	164.0	170.2	176.3	164.0
	Schizophrenia		61.2	56.8	28.9	56.1	54.4	63.2	61.4	3.7	54.8	58.9	51.7	34.2	58.7
	Autism spectrum disorders		108.3	104.3	73.0	99.3	98.5	65.9	100.3	81.3	96.5	71.3	11.1	130.3	72.3
	Other mental disorders		419.8	367.7	326.9	365.5	365.2	328.2	367.9	434.4	364.3	329.7	365.9	341.0	328.0
	Eating disorders		107.9	126.1	181.8	132.4	102.2	98.1	101.3	111.8	96.5	52.6	109.9	98.2	46.3
	Idiopathic developmental intellectual di		294.6	194.7	214.6	237.0	250.4	118.1	89.0	332.4	283.0	224.2	66.6	263.1	252.0

	x higher than ave of other HiSDI		USA	Andorra	Australia	Austria	Belgium	Bermuda	Brunei	Canada	Cyprus	Czechia	Denmark	England	Estonia
	**All Firearm Deaths**	10	10.08	0.52	0.8	1.71	1.49	1.69	0.14	2.02	1.21	1.14	0.83	0.18	1.04
	Firearm Suicides	8	5.76	0.42	0.58	1.55	1.1	0.04	0.05	1.47	0.64	0.92	0.66	0.12	0.71
	Firearm Homicides	19	4.12	0.08	0.18	0.13	0.34	1.5	0.07	0.5	0.51	0.15	0.15	0.04	0.27
	Unintentional Firearm Deaths	4	0.2	0.02	0.04	0.03	0.05	0.15	0.02	0.05	0.06	0.07	0.02	0.02	0.06
	Self-harm by firearm		6.5	1.55	0.83	1.81	1.43	0.05	0.07	1.9	0.8	1.23	0.81	0.19	1
Hi CI	Physical violence by firearm		4.25	0.12	0.19	0.14	0.36	1.81	0.09	0.55	0.64	0.18	0.16	0.04	0.34
	Unintentional firearm injuries		0.24	0.04	0.06	0.04	0.06	0.2	0.02	0.08	0.09	0.09	0.04	0.03	0.09
	Self-harm by firearm		5.42	0.15	0.49	1.06	0.88	0.03	0.03	1.24	0.25	0.63	0.47	0.11	0.4
Lo CI	Physical violence by firearm		3.83	0.05	0.16	0.12	0.31	1.23	0.05	0.45	0.41	0.12	0.13	0.03	0.2
	Unintentional firearm injuries		0.17	0.01	0.03	0.02	0.03	0.11	0.01	0.04	0.04	0.05	0.02	0.02	0.04
Hi Ci - I	All Firearm Deaths		0.91	1.19	0.28	0.28	0.36	0.37	0.04	0.51	0.32	0.36	0.18	0.08	0.39
Mean -	All Firearm Deaths		0.66	0.31	0.12	0.51	0.27	0.32	0.05	0.29	0.51	0.34	0.21	0.02	0.4
Hi Ci - I	Firearm Suicides		0.74	1.13	0.25	0.26	0.33	0.01	0.02	0.43	0.16	0.31	0.15	0.07	0.29
Mean -	Firearm Suicides		0.34	0.27	0.09	0.49	0.22	0.01	0.02	0.23	0.39	0.29	0.19	0.01	0.31
Hi Ci - I	Firearm Homicides		0.13	0.04	0.01	0.01	0.02	0.31	0.02	0.05	0.13	0.03	0.01	0	0.07
Mean -	Firearm Homicides		0.29	0.03	0.02	0.01	0.03	0.27	0.02	0.05	0.1	0.03	0.02	0.01	0.07

x higher in U.S. than average of all other high SDI countries

Finland	France	Germany	Guam	Iceland	Ireland	Japan	Kuwait	Latvia	Lithuania	Luxembourg	Monaco	Netherlands	New Zealan	Northern Ire	Norway	Qatar
13,626.5	15,099.9	13,684.0	11,025.9	13,305.0	15,973.4	9,687.7	13,120.6	11,677.5	12,314.0	13,539.0	14,580.0	15,051.3	17,203.5	15,662.2	14,743.7	12,786.5
3,993.1	6,274.3	6,208.1	4,014.0	5,103.8	6,907.8	2,324.1	4,600.0	3,748.0	4,254.2	5,277.0	5,501.6	6,754.8	7,263.5	6,520.9	6,735.8	4,263.9
4,139.3	3,835.7	3,376.8	3,197.2	2,936.6	4,216.9	2,103.3	4,060.3	3,747.8	3,991.2	3,219.3	4,056.5	3,534.4	3,723.9	4,258.9	3,238.2	3,951.4
1,633.6	1,500.1	638.0	1,133.1	1,496.4	1,642.9	1,491.1	1,000.2	1,059.8	1,058.6	1,296.0	1,285.5	1,471.5	2,557.2	1,289.0	1,595.5	1,112.0
890.3	904.2	746.9	285.0	908.4	701.3	644.3	810.2	520.1	521.2	896.1	900.8	787.7	1,506.4	1,134.1	828.6	744.6
626.7	626.8	629.3	532.5	626.0	626.5	584.0	589.6	583.9	583.8	628.4	625.6	594.3	639.1	627.0	648.5	600.3
264.1	268.3	262.7	327.2	278.4	352.7	300.8	273.8	286.0	288.4	281.9	295.4	368.0	388.6	275.7	282.7	285.0
557.7	457.1	572.3	296.3	566.1	607.3	676.5	305.0	401.0	407.8	571.4	548.3	629.6	437.3	582.7	484.5	344.7
1,603.3	1,593.6	1,606.1	1,473.3	1,607.6	1,595.6	1,460.7	1,492.9	1,452.3	1,451.8	1,604.6	1,595.1	1,600.5	1,556.9	1,595.5	1,465.6	1,606.2
481.8	468.3	425.6	182.9	454.1	455.8	388.8	345.8	166.1	173.4	568.8	869.9	383.7	603.4	419.92	474.7	346.4
438.2	456.9	279.7	236.5	319.4	300.1	228.3	591.0	431.4	390.3	228.0	98.2	213.9	348.3	340.23	226.0	428.4
14,895.6	16,622.6	15,073.4	12,120.8	14,657.8	17,380.6	10,468.8	14,354.4	12,766.9	13,450.8	14,867.6	16,009.9	16,748.1	18,595.5	17,157.1	15,882.5	13,934.4
5,009.1	7,816.0	7,682.8	4,967.1	6,358.6	8,441.3	2,701.7	5,713.8	4,699.8	5,371.8	6,572.1	6,863.2	8,573.7	8,616.4	8,156.3	7,843.5	5,263.8
4,737.2	4,368.1	3,826.9	3,700.1	3,397.3	4,768.9	2,324.9	4,676.5	4,257.9	4,542.7	3,665.2	4,809.1	4,052.8	4,205.1	4,832.8	3,633.8	4,517.4
2,190.1	2,014.5	870.7	1,569.4	2,016.8	2,259.1	2,009.1	1,354.4	1,422.9	1,421.3	1,770.4	1,756.1	1,970.2	3,503.5	1,761.8	2,158.2	1,508.7
1,114.4	1,127.5	877.6	355.7	1,132.6	837.9	753.3	1,021.6	662.0	658.7	1,111.7	1,126.3	875.0	1,769.8	1,333.5	961.0	936.5
809.8	809.8	812.7	695.5	809.0	809.5	764.9	760.6	763.8	763.6	811.9	808.7	763.6	830.1	810.0	826.7	771.0
318.8	326.2	322.0	400.8	341.9	431.2	345.7	334.0	348.5	349.6	344.5	361.0	437.4	448.8	317.3	323.5	351.1
666.1	533.8	687.2	360.6	673.2	727.2	805.0	370.1	480.9	489.8	687.5	652.2	754.7	524.2	706.7	577.4	415.7
2,065.3	2,052.0	2,069.3	1,881.6	2,071.1	2,054.5	1,851.2	1,904.0	1,858.6	1,857.8	2,066.8	2,054.0	2,061.4	1,979.9	2,054.5	1,856.0	2,063.6
607.4	591.7	525.2	235.0	574.4	576.3	490.5	446.0	214.4	225.0	722.9	1,099.0	473.7	762.6	528.94	597.3	452.5
749.7	734.7	507.8	403.4	568.4	551.8	436.2	937.9	698.3	649.2	425.9	219.1	410.3	598.0	596.08	419.5	709.7
12,589.4	13,786.0	12,402.5	10,083.1	12,156.9	14,642.2	8,998.8	12,025.8	10,738.1	11,324.4	12,409.6	13,375.1	13,744.6	15,982.3	14,350.8	13,647.2	11,750.6
3,191.7	4,991.2	4,983.5	3,185.4	4,080.3	5,544.1	1,986.9	3,628.0	2,999.7	3,385.3	4,188.2	4,353.5	5,318.1	6,166.7	5,174.7	5,787.0	3,353.9
3,662.2	3,364.8	2,972.8	2,782.9	2,563.1	3,752.3	1,902.6	3,523.3	3,317.3	3,515.4	2,850.3	3,413.3	3,097.1	3,296.4	3,726.9	2,866.7	3,427.3
1,189.0	1,052.0	444.7	803.9	1,069.9	1,157.3	1,057.7	718.6	759.0	758.1	942.8	933.5	1,054.8	1,850.7	936.9	1,148.1	796.1
692.4	704.5	620.7	221.2	704.4	575.8	541.2	623.7	403.7	407.9	690.8	702.5	704.7	1,259.7	941.3	697.1	573.9
456.5	456.5	458.6	372.6	456.0	456.3	430.2	430.6	420.0	419.9	458.1	455.4	429.5	466.6	456.8	475.2	440.8
212.6	212.9	208.3	259.3	225.5	281.7	258.6	216.4	227.9	229.7	224.8	234.0	299.9	332.8	236.7	242.3	225.4
460.9	390.0	467.4	240.7	471.1	500.5	562.8	248.0	331.4	336.7	470.4	453.3	515.2	360.3	479.5	399.4	283.2
1,237.2	1,229.5	1,239.4	1,141.5	1,240.7	1,231.1	1,119.4	1,154.7	1,125.5	1,125.0	1,238.3	1,230.7	1,235.0	1,199.0	1,231.1	1,123.4	1,234.5
371.3	358.7	334.3	135.1	345.4	344.5	300.6	249.9	121.1	127.8	431.0	674.8	300.5	445.6	318.06	361.3	252.6
140.6	157.3	57.1	71.9	80.5	70.9	36.3	252.7	155.2	133.8	52.5	15.9	44.2	110.6	83.12	55.1	157.8
1,269.1	1,522.7	1,389.4	1,094.9	1,352.9	1,407.2	781.1	1,233.8	1,089.4	1,136.8	1,328.6	1,429.9	1,696.8	1,391.9	1,494.8	1,138.8	1,147.9
1,015.9	1,541.7	1,474.7	953.2	1,254.8	1,533.5	377.6	1,113.9	951.8	1,117.6	1,295.1	1,361.6	1,818.9	1,352.9	1,635.4	1,107.6	999.9
597.9	532.3	450.1	502.9	460.7	552.0	221.6	616.2	510.1	551.5	445.9	752.6	518.4	481.2	573.9	395.6	566.0
556.6	514.4	232.7	436.3	520.5	616.2	518.0	354.2	363.2	362.8	474.5	470.7	498.8	946.2	472.7	562.7	396.7
224.2	223.3	130.7	70.7	224.2	136.6	109.0	211.4	141.9	137.6	215.6	225.6	87.3	263.4	199.4	132.4	191.9
183.0	183.0	183.4	163.0	183.0	183.0	180.9	171.0	179.9	179.9	183.5	183.1	169.3	191.0	183.0	178.2	170.7
54.7	57.9	59.3	73.6	63.5	78.5	44.9	60.2	62.5	61.2	62.6	65.6	69.4	60.2	41.6	40.8	66.2
108.5	76.6	114.9	64.3	107.1	119.9	128.5	65.1	79.8	82.0	116.1	104.0	125.1	87.0	124.0	93.0	71.0
462.1	458.4	463.2	408.3	463.5	458.9	390.6	411.1	406.3	406.0	462.2	458.9	460.9	423.0	459.0	390.4	457.4
125.6	123.5	99.6	52.2	120.3	120.5	101.7	100.2	48.4	51.7	154.1	229.1	90.0	159.3	109.0	122.6	106.0
311.5	277.8	228.1	166.9	249.0	251.6	208.0	346.9	267.0	258.9	198.0	120.9	196.4	249.6	255.9	193.5	281.3
1,037.1	1,313.9	1,281.5	942.8	1,148.1	1,331.2	689.0	1,094.8	939.5	989.7	1,129.4	1,204.9	1,306.6	1,221.2	1,311.4	1,096.5	1,035.9
801.4	1,283.0	1,224.6	828.6	1,023.5	1,363.7	337.3	972.0	748.4	868.9	1,088.8	1,148.1	1,436.7	1,096.8	1,346.3	948.9	910.0
477.2	471.0	404.0	414.3	373.5	464.7	200.7	537.0	430.5	475.8	369.0	643.2	437.3	427.5	532.0	371.5	524.1
444.6	448.1	193.3	329.2	426.5	485.6	433.4	281.6	300.7	300.4	353.1	351.9	416.7	706.6	352.1	447.4	315.9
197.9	199.7	126.2	63.7	204.0	125.5	103.1	186.5	116.4	113.3	205.3	198.3	82.9	246.7	192.8	131.5	170.7
170.2	170.2	170.7	160.0	169.9	170.1	153.8	159.0	164.0	163.9	170.3	170.2	164.8	172.5	170.3	173.3	159.5
51.5	55.4	54.4	67.9	52.9	71.0	42.2	57.4	58.1	58.8	57.1	61.4	68.1	55.8	39.0	40.4	59.5
96.8	67.2	104.9	55.6	95.0	106.8	113.7	57.0	69.6	71.1	101.0	95.0	114.4	76.9	103.2	85.1	61.5
366.1	364.1	366.7	331.8	366.9	364.5	341.2	338.2	326.9	326.7	366.3	364.4	365.5	357.9	364.5	342.2	371.7
110.5	109.6	91.3	47.8	108.8	111.3	88.2	96.0	45.0	45.6	137.8	195.2	83.2	157.7	101.9	113.5	93.8
297.6	299.6	222.6	164.6	238.9	229.3	192.0	338.3	276.2	256.5	175.5	82.3	169.7	237.7	257.1	170.9	270.6

Finland	France	Germany	Guam	Iceland	Ireland	Japan	Kuwait	Latvia	Lithuania	Luxembourg	Monaco	Netherlands	New Zealan	Northern Ire	Norway	Qatar
2.26	2.17	0.71	2.11	0.95	0.56	0.06	0.36	1.13	0.85	1.02	1.94	0.44	0.92	0.29	1.2	0.25
2.01	1.7	0.6	1.46	0.87	0.34	0.03	0.07	0.63	0.47	0.77	1.81	0.2	0.71	0.2	1.11	0.05
0.21	0.32	0.08	0.58	0.06	0.18	0.02	0.28	0.42	0.3	0.21	0.09	0.23	0.15	0.06	0.07	0.13
0.04	0.15	0.03	0.07	0.02	0.04	0.01	0.01	0.08	0.08	0.04	0.04	0.01	0.06	0.03	0.02	0.07
2.73	2.07	0.73	2.15	1.08	0.73	0.04	0.1	0.89	0.66	0.95	2.8	0.29	0.99	0.43	1.73	0.12
0.24	0.35	0.09	0.78	0.06	0.2	0.02	0.35	0.53	0.36	0.24	0.13	0.25	0.16	0.06	0.08	0.2
0.04	0.18	0.04	0.21	0.03	0.05	0.02	0.02	0.12	0.11	0.05	0.06	0.02	0.08	0.04	0.03	0.12
1.71	1.28	0.44	0.85	0.64	0.25	0.02	0.06	0.4	0.32	0.49	1.3	0.16	0.6	0.15	0.97	0.03
0.2	0.29	0.08	0.42	0.05	0.16	0.02	0.22	0.34	0.24	0.18	0.06	0.21	0.14	0.05	0.07	0.07
0.03	0.1	0.03	0.04	0.02	0.02	0.01	0.01	0.05	0.06	0.03	0.03	0.01	0.05	0.02	0.02	0.04
0.75	0.43	0.15	1.03	0.22	0.42	0.02	0.11	0.41	0.28	0.22	1.05	0.12	0.31	0.24	0.64	0.19
0.32	0.5	0.16	0.8	0.24	0.13	0.01	0.07	0.34	0.23	0.32	0.55	0.06	0.13	0.07	0.14	0.11
0.72	0.37	0.13	0.69	0.21	0.39	0.01	0.03	0.26	0.19	0.18	0.99	0.09	0.28	0.23	0.62	0.07
0.3	0.42	0.16	0.61	0.23	0.09	0.01	0.01	0.23	0.15	0.28	0.51	0.04	0.11	0.05	0.14	0.02
0.03	0.03	0.01	0.2	0	0.02	0	0.07	0.11	0.06	0.03	0.04	0.02	0.01	0	0.01	0.07
0.01	0.03	0	0.16	0.01	0.02	0	0.06	0.08	0.06	0.03	0.03	0.02	0.01	0.01	0	0.06

San Marino	Scotland	Singapore	Slovakia	Slovenia	South Korea	Sweden	Switzerland	Taiwan	UAE	Wales	Puerto Rico	Russia	Saudi Arabia	UK
14,500.1	12,871.3	9,900.6	10,422.8	10,761.0	10,033.6	14,109.8	15,489.5	9,806.2	11,920.0	13,163.4	13,490.2	10,496.0	13,071.9	13,777.7
5,542.6	4,217.4	2,708.1	3,425.7	3,389.2	3,221.3	4,795.1	6,790.6	3,665.5	4,243.3	4,260.7	4,442.6	3,133.7	4,554.4	4,460.2
4,091.9	3,938.8	2,008.1	2,695.3	3,115.2	2,091.2	4,116.5	3,705.5	2,564.1	3,578.5	4,146.4	2,882.8	3,079.9	4,216.1	4,097.6
1,278.9	890.7	1,437.5	1,061.2	1,064.8	1,372.3	1,159.2	1,565.5	940.9	442.1	939.4	3,059.2	1,086.4	929.5	1,258.3
918.9	913.9	719.8	542.3	546.8	500.3	973.9	941.7	193.8	752.0	901.5	947.1	514.1	762.2	1,094.5
625.1	632.8	528.0	584.1	584.5	502.4	662.8	628.1	501.5	587.7	631.8	560.6	625.3	584.8	677.4
280.1	242.8	319.2	295.7	299.5	300.6	270.3	280.0	313.4	275.3	271.0	292.8	281.8	262.9	246.3
560.4	556.0	588.1	374.6	382.7	548.9	706.8	573.4	215.8	331.2	546.4	347.8	396.8	310.9	739.6
1,585.1	1,593.7	1,608.1	1,461.5	1,468.8	1,609.1	1,464.5	1,603.5	1,463.6	1,583.7	1,601.6	1,452.4	1,349.4	1,517.7	1,480.3
505.2	404.63	453.7	182.7	184.9	351.4	514.2	491.1	173.6	300.4	410.58	310.9	161.6	279.0	425.0
279.6	373.6	75.2	396.3	350.8	91.5	550.3	273.0	288.9	609.5	396.93	199.8	451.5	586.5	329.7
16,021.5	13,993.9	10,913.7	11,415.4	11,762.1	11,041.2	15,138.9	16,889.8	10,816.0	13,073.5	14,345.8	14,985.2	11,291.2	14,277.9	14,783.9
7,022.9	5,192.6	3,370.1	4,318.0	4,201.1	4,047.1	5,624.2	8,161.9	4,565.8	5,228.0	5,365.0	5,566.5	3,642.1	5,680.0	5,226.9
4,866.4	4,511.7	2,297.1	3,126.3	3,588.7	2,363.1	4,573.0	4,182.3	3,027.2	4,133.3	4,734.5	3,306.4	3,429.2	4,857.1	4,602.1
1,749.9	1,199.9	2,006.6	1,423.8	1,428.4	1,832.5	1,539.6	2,121.9	1,268.7	607.4	1,284.3	4,110.2	1,498.2	1,260.3	1,754.7
1,138.4	1,137.8	882.8	687.8	692.1	620.2	1,137.6	1,175.9	242.0	956.0	1,119.6	1,192.2	600.7	947.8	1,277.8
808.3	815.6	700.7	763.8	764.3	665.5	850.4	811.3	659.7	764.2	819.4	724.5	806.4	758.3	866.0
343.5	269.5	385.6	359.2	365.0	366.4	308.5	339.3	383.0	337.2	327.9	358.7	324.0	323.1	283.1
669.9	663.1	707.6	451.2	461.9	653.7	838.5	688.4	256.4	400.8	652.5	418.7	474.3	375.6	876.3
2,039.9	2,052.1	2,071.1	1,869.0	1,877.2	2,072.6	1,855.0	2,065.5	1,870.6	2,030.6	2,063.0	1,857.9	1,709.2	1,939.3	1,877.6
642.4	511.73	576.6	235.3	238.2	452.8	662.1	607.5	221.8	395.6	519.47	406.6	208.0	358.1	532.7
510.6	651.34	188.2	637.0	592.4	209.5	889.1	498.6	473.4	961.3	700.2	355.3	734.9	924.3	604.8
13,274.7	11,896.6	9,096.4	9,501.9	9,922.6	9,165.4	13,187.5	14,226.3	8,876.3	10,879.5	12,142.4	12,250.1	9,749.0	12,043.0	12,895.5
4,397.4	3,350.0	2,135.1	2,743.4	2,711.6	2,563.4	4,113.7	5,513.7	2,904.7	3,386.0	3,377.5	3,527.1	2,685.8	3,598.7	3,806.9
3,442.5	3,429.4	1,780.4	2,361.9	2,737.6	1,858.5	3,726.6	3,307.8	2,211.7	3,093.1	3,652.8	2,508.4	2,749.0	3,680.7	3,649.6
929.5	626.0	1,028.0	760.6	763.9	1,012.7	839.8	1,125.4	679.1	309.1	672.7	2,243.2	774.7	669.6	897.8
708.0	701.9	563.4	422.3	424.4	391.5	814.1	726.3	151.3	586.6	699.6	727.7	436.0	589.5	910.1
455.2	466.6	381.2	420.1	420.5	349.2	488.3	457.7	355.7	425.0	467.5	406.7	461.5	428.3	503.1
223.0	215.5	255.6	236.5	239.8	241.0	233.7	224.3	245.9	218.5	216.0	232.5	242.0	208.4	211.9
457.6	461.5	485.4	307.4	311.5	452.7	589.1	470.7	183.2	271.9	452.6	284.4	327.0	253.2	617.2
1,222.8	1,229.6	1,241.2	1,132.7	1,138.3	1,241.8	1,122.6	1,237.4	1,134.4	1,216.5	1,235.9	1,125.6	1,030.4	1,171.7	1,141.2
381.9	306.46	342.9	133.2	135.2	260.0	378.4	384.5	128.8	216.3	315.62	228.5	119.0	202.5	324.9
67.7	110.5	3.3	151.4	120.8	10.1	228.6	67.0	110.0	259.5	111.7	48.3	170.2	248.9	87.5
1,521.4	1,122.5	1,013.1	992.6	1,001.1	1,007.7	1,029.1	1,400.3	1,009.8	1,153.5	1,182.3				
1,480.3	975.2	662.0	892.3	811.9	825.8	829.0	1,371.3	900.3	984.7	1,104.4				
774.5	572.9	289.0	431.0	473.5	272.0	456.5	476.8	463.1	554.8	588.1				
471.0	309.3	569.2	362.6	363.6	460.2	380.4	556.5	327.8	165.3	345.0				
219.5	224.0	163.1	145.5	145.4	119.9	163.7	234.1	48.2	204.0	218.1				
183.2	182.7	172.7	179.8	179.7	163.1	187.6	183.1	158.2	176.5	187.6				
63.3	26.8	66.4	63.5	65.5	65.8	38.3	59.3	69.6	61.9	56.9				
109.4	107.1	119.5	76.7	79.3	104.8	131.7	115.0	40.6	69.6	106.1				
454.8	458.4	462.9	407.5	408.4	463.5	390.5	462.0	407.1	446.8	461.4				
137.3	107.1	123.0	52.6	53.3	101.5	147.9	116.4	48.2	95.2	108.9				
231.0	277.7	113.0	240.7	241.7	117.9	338.8	225.6	184.5	351.9	303.3				
1,225.4	974.8	804.2	920.9	838.5	868.1	922.3	1,263.2	929.9	1,040.5	1,021.0				
1,145.3	867.4	573.0	682.2	677.6	658.0	681.5	1,276.9	760.8	857.2	883.1				
649.4	509.4	227.7	333.4	377.6	232.7	389.9	397.7	352.3	485.4	493.6				
349.5	264.6	409.5	300.6	301.0	359.6	319.4	440.1	261.7	132.9	266.7				
210.9	212.0	156.4	120.0	122.4	108.8	159.9	215.5	42.5	165.4	201.9				
169.9	166.2	146.9	164.0	164.0	153.3	174.5	170.4	145.8	162.6	164.4				
57.1	27.2	63.6	59.1	59.7	59.6	36.6	55.6	67.5	56.8	55.1				
102.8	94.6	102.8	67.2	71.2	96.2	117.7	102.7	32.6	59.3	93.8				
362.3	364.1	366.9	328.8	330.5	367.3	341.9	366.1	329.2	367.3	365.7				
123.3	98.2	110.8	49.5	49.8	91.4	135.7	106.5	44.8	84.1	95.0				
211.9	263.1	71.9	245.0	230.0	81.4	321.7	206.0	178.9	350.0	285.3				

San Marino	Scotland	Singapore	Slovakia	Slovenia	South Korea	Sweden	Switzerland	Taiwan	UAE	Wales	Puerto Rico	Russia	Saudi Arabia	UK
2.98	0.33	0.04	1.14	1.22	0.06	1.13	1.91	0.19	0.75	0.26	20.81	1.5	1.25	0.2
2.86	0.23	0.02	0.73	1.02	0.03	0.85	1.69	0.06	0.33	0.19	0.65	0.6	0.26	0.14
0.08	0.07	0.01	0.25	0.18	0.02	0.25	0.18	0.11	0.3	0.05	19.72	0.72	0.17	0.04
0.04	0.03	0.01	0.16	0.02	0.01	0.03	0.04	0.02	0.12	0.02	0.44	0.18	0.82	0.02
4.76	0.46	0.03	1.1	1.45	0.05	1.17	1.99	0.09	0.6	0.36	0.89	0.88	0.37	0.22
0.12	0.08	0.01	0.34	0.23	0.03	0.28	0.2	0.14	0.58	0.05	24.36	0.86	0.34	0.04
0.06	0.04	0.02	0.24	0.04	0.02	0.03	0.05	0.03	0.48	0.04	0.56	0.23	1.4	0.03
1.68	0.17	0.01	0.43	0.67	0.02	0.7	1.23	0.04	0.14	0.15	0.47	0.45	0.13	0.12
0.05	0.06	0.01	0.18	0.14	0.02	0.23	0.17	0.09	0.12	0.04	15.86	0.6	0.11	0.04
0.02	0.02	0.01	0.08	0.01	0.01	0.02	0.03	0.02	0.03	0.02	0.34	0.13	0.44	0.02
1.96	0.25	0.02	0.54	0.5	0.04	0.35	0.33	0.07	0.91	0.19		0.47	0.86	0.09
1.23	0.08	0.01	0.45	0.4	0.01	0.18	0.48	0.04	0.46	0.05		0.32	0.57	0.02
1.9	0.23	0.01	0.37	0.43	0.02	0.32	0.3	0.03	0.27	0.17		0.28	0.11	0.08
1.18	0.06	0.01	0.3	0.35	0.01	0.15	0.46	0.02	0.19	0.04		0.15	0.13	0.02
0.04	0.01	0	0.09	0.05	0.01	0.03	0.02	0.03	0.28	0		0.14	0.17	0
0.03	0.01	0	0.07	0.04	0	0.02	0.01	0.02	0.18	0.01		0.12	0.06	0
