# Supplementary material for: Fallacy of attributing the U.S. firearm mortality epidemic to mental health
Source: PLoS One. 2024 Aug 5;19(8):e0290138. doi: 10.1371/journal.pone.0290138 (PMC11299823; doi:10.1371/journal.pone.0290138)
Supplement: S5 File — (PDF) [file pone.0290138.s007.pdf]

	U.S., Age-standardized							INCIDENCE										
								MENTAL DISORDERS										
	FIREARMS							IHME										
Source:	WISQARS							All	Category titles are listed below							All		Cat
Rates	All Firea	omicide	%	Suicide	%	Uninten	%	1	2	3	4	5	6	7	8	1	2	3
1990	14.51	6.11	42%	7.61	52%	0.55	4%	5,253	24.1	754.7	113.4	13.4	51.9	188.5	3850.0	14515.8	435.9	5241.2
1991	14.82	6.64	45%	7.39	50%	0.57	4%	5,419	24.3	754.6	115.5	13.4	51.9	186.3	4008.2	14650.4	439.4	5241.6
1992	14.46	6.5	45%	7.15	49%	0.54	4%	5,586	24.5	756.0	117.5	13.4	51.8	184.3	4167.6	14785.1	442.6	5247.8
1993	15	6.75	45%	7.34	49%	0.58	4%	5,749	24.6	758.7	119.1	13.5	51.8	182.8	4322.9	14913.8	445.3	5259.0
1994	14.45	6.45	45%	7.18	50%	0.51	4%	5,903	24.8	762.5	120.4	13.5	51.8	181.8	4468.8	15031.5	447.3	5274.7
1995	13.38	5.69	43%	6.99	52%	0.46	3%	6,041	24.8	767.2	121.1	13.5	51.8	181.5	4600.0	15132.3	448.4	5293.7
1996	12.57	5.1	41%	6.79	54%	0.42	3%	6,216	24.8	787.8	121.6	13.5	51.8	181.8	4754.1	15329.7	448.9	5452.5
1997	11.84	4.77	40%	6.48	55%	0.36	3%	6,450	24.8	829.8	122.0	13.6	51.8	182.3	4946.2	15667.2	449.1	5800.8
1998	11.09	4.21	38%	6.34	57%	0.31	3%	6,690	24.8	878.1	122.3	13.6	51.8	183.0	5137.6	16040.3	449.3	6209.0
1999	10.3	3.82	37%	5.96	58%	0.29	3%	6,882	24.8	917.9	122.4	13.6	51.8	183.6	5290.0	16344.4	449.5	6547.9
2000	10.1	3.78	37%	5.88	58%	0.27	3%	6,973	24.8	934.4	122.5	13.7	51.8	183.8	5365.1	16476.6	449.9	6688.4
2001	10.3	3.93	38%	5.9	57%	0.28	3%	6,980	24.9	928.4	122.7	13.7	51.8	183.7	5377.4	16445.3	451.4	6636.5
2002	10.4	4.07	39%	5.92	57%	0.26	2%	6,963	25.0	914.0	123.0	13.7	51.8	183.2	5373.4	16348.5	454.2	6510.1
2003	10.3	4.07	40%	5.77	56%	0.25	2%	6,936	25.2	895.6	123.4	13.7	51.8	182.6	5362.5	16217.9	457.4	6347.8
2004	10.0	3.94	39%	5.65	57%	0.22	2%	6,911	25.3	877.9	123.7	13.7	51.8	182.1	5353.9	16090.4	460.2	6188.5
2005	10.3	4.17	41%	5.66	55%	0.27	3%	6,902	25.4	865.2	123.7	13.7	51.8	181.8	5357.1	15998.5	461.7	6070.7
2006	10.2	4.27	42%	5.54	54%	0.21	2%	6,918	25.4	859.3	123.6	14.0	51.8	181.8	5377.7	15953.8	462.0	6000.6
2007	10.2	4.2	41%	5.63	55%	0.2	2%	6,949	25.4	857.3	123.5	14.6	51.9	181.8	5409.2	15933.6	462.1	5950.4
2008	10.2	4.03	39%	5.82	57%	0.19	2%	6,983	25.5	856.6	123.3	15.3	52.0	181.9	5442.5	15921.6	461.8	5908.2
2009	10.1	3.78	38%	5.91	59%	0.18	2%	7,008	25.5	854.8	123.1	15.8	52.0	182.0	5468.4	15899.8	461.3	5862.3
2010	10.1	3.62	36%	6.06	60%	0.2	2%	7,013	25.5	849.5	123.0	16.1	52.0	182.1	5477.8	15853.1	460.5	5800.8
2011	10.2	3.59	35%	6.16	61%	0.19	2%	6,958	25.4	836.2	122.9	16.1	52.0	184.0	5436.3	15748.3	458.5	5690.2
2012	10.5	3.76	36%	6.29	60%	0.17	2%	6,842	25.1	816.1	122.7	16.1	52.0	188.1	5339.1	15586.9	455.0	5531.8
2013	10.4	3.6	35%	6.38	61%	0.16	2%	6,703	24.9	794.4	122.6	16.1	52.0	192.9	5220.7	15413.4	450.6	5365.8
2014	10.3	3.53	34%	6.36	62%	0.14	1%	6,581	24.6	776.7	122.5	16.1	52.0	197.0	5115.4	15268.3	446.1	5232.7
2015	11.0	4.14	38%	6.5	59%	0.15	1%	6,515	24.4	768.1	122.5	16.0	52.0	198.6	5058.1	15193.9	442.4	5172.5
2016	11.7	4.6	39%	6.72	57%	0.15	1%	6,506	24.1	768.1	124.1	16.1	51.9	198.6	5044.7	15197.5	436.4	5168.1
2017	12.0	4.61	39%	6.93	58%	0.15	1%	6,508	23.8	771.6	125.7	16.3	51.8	198.6	5039.4	15217.0	431.8	5175.3
2018	11.9	4.42	37%	7.02	59%	0.14	1%	6,526	23.8	788.8	125.7	16.4	51.9	198.6	5039.5	15355.2	431.9	5342.1
2019	11.8	4.57	39%	6.83	58%	0.15	1%	6,569	23.8	822.1	125.7	16.5	52.3	198.6	5047.9	15654.9	433.7	5697.8
2020	13.6	6.16	45%	6.93	51%	0.17	1%											

Pearson	All Fireaomicide Firearm Suicide Unintentional Fi	Mental	Schizopl	Anxiety	Attentic	Autism :	Bipolar	Conduct	Depress	All Menta	Schizopl	Anxiety
All Firearm Deaths	1990-2019	-0.96	-0.55	-0.73	-0.72	-0.37	-0.20	0.04	-0.96	-0.83	-0.61	-0.63
	1995-2019	-0.93	-0.63	-0.63	0.01	0.00	-0.07	0.31	-0.94	-0.65	-0.66	-0.55
	2000-2019	-0.85	-0.93	-0.62	0.80	0.54	0.23	0.84	-0.87	-0.63	-0.92	-0.58
	2005-2019	-0.84	-0.95	-0.67	0.82	0.49	0.06	0.83	-0.87	-0.66	-0.94	-0.61
	2010-2019	-0.78	-0.95	-0.51	0.89	0.73	-0.18	0.78	-0.82	-0.50	-0.95	-0.42
	2015-2019	0.18	-0.92	0.36	0.94	0.68	-0.08	-0.83	-0.93	0.35	-0.97	0.31
Firearm Homicides	1990-2019	-0.93	-0.42	-0.65	-0.72	-0.50	-0.29	-0.11	-0.93	-0.75	-0.47	-0.53
	1995-2019	-0.80	-0.39	-0.43	-0.02	-0.28	-0.27	0.03	-0.83	-0.45	-0.40	-0.34
	2000-2019	-0.51	-0.61	-0.25	0.82	0.05	-0.11	0.43	-0.56	-0.25	-0.56	-0.20
	2005-2019	-0.51	-0.63	-0.22	0.82	-0.07	-0.24	0.42	-0.56	-0.21	-0.59	-0.14
	2010-2019	-0.72	-0.89	-0.45	0.85	0.68	-0.17	0.72	-0.76	-0.44	-0.89	-0.37
	2015-2019	0.13	-0.72	0.27	0.74	0.50	-0.06	-0.63	-0.75	0.28	-0.76	0.25
Firearm Suicides	1990-2019	-0.91	-0.76	-0.83	-0.58	-0.04	0.04	0.37	-0.90	-0.89	-0.81	-0.77
	1995-2019	-0.86	-0.80	-0.76	0.15	0.41	0.24	0.65	-0.84	-0.79	-0.85	-0.73
	2000-2019	-0.87	-0.90	-0.76	0.56	0.80	0.46	0.93	-0.86	-0.78	-0.92	-0.74
	2005-2019	-0.86	-0.93	-0.84	0.56	0.82	0.29	0.92	-0.86	-0.84	-0.95	-0.83
	2010-2019	-0.84	-0.97	-0.57	0.88	0.75	-0.20	0.84	-0.87	-0.57	-0.98	-0.49
	2015-2019	0.18	-0.93	0.35	0.94	0.71	-0.11	-0.84	-0.92	0.33	-0.97	0.29
FA Unint	1990-2019	-0.82	-0.14	-0.36	-0.85	-0.76	-0.48	-0.44	-0.84	-0.52	-0.21	-0.22
	1995-2019	-0.37	0.22	0.20	-0.66	-0.81	-0.61	-0.63	-0.45	0.17	0.18	0.29
	2000-2019	0.75	0.53	0.93	-0.36	-0.91	-0.64	-0.76	0.65	0.92	0.56	0.93
	2005-2019	0.74	0.73	0.82	-0.23	-0.85	-0.38	-0.82	0.71	0.82	0.77	0.83
	2010-2019	0.94	0.85	0.84	-0.44	-0.48	0.17	-0.94	0.94	0.83	0.85	0.78
	2015-2019	-0.46	-0.24	-0.01	0.14	0.04	0.05	0.64	-0.53	0.01	-0.25	0.04

PREVALENCE
MENTAL DISORDERS
IHME
:egory titles are listed below

4	5	6	7	8	9	10
1867.1	518.5	606.2	529.3	3796.5	404.1	534.1
1910.1	518.9	605.9	521.8	3902.6	418.5	531.5
1949.4	519.4	605.7	515.3	4007.0	431.5	529.0
1983.2	519.9	605.5	510.1	4106.5	442.0	526.5
2009.5	520.4	605.3	506.7	4198.1	449.2	524.2
2026.2	520.9	605.1	505.4	4278.9	452.3	522.1
2037.0	521.5	604.9	506.0	4346.0	452.2	519.9
2046.3	522.1	604.7	507.6	4398.7	451.2	517.3
2054.0	522.6	604.5	509.6	4439.0	449.5	514.6
2060.3	523.2	604.3	511.3	4467.7	448.2	512.0
2064.9	523.7	604.1	512.2	4485.8	447.7	509.9
2069.9	524.2	603.9	511.7	4493.3	448.8	507.9
2076.3	524.7	603.8	510.4	4492.3	450.6	505.8
2082.7	525.2	603.6	508.6	4486.7	452.8	503.5
2087.7	525.6	603.5	507.1	4482.2	454.8	501.0
2089.8	526.0	603.3	506.3	4484.8	456.2	498.4
2090.0	536.5	603.1	506.2	4491.8	457.2	492.7
2089.9	560.7	602.8	506.2	4498.4	458.2	482.7
2089.4	589.6	602.5	506.4	4503.6	459.2	471.4
2088.4	613.6	602.3	506.7	4506.4	459.9	461.5
2087.0	623.8	602.1	507.0	4506.5	460.1	455.9
2083.6	623.9	602.0	512.3	4495.2	455.5	453.9
2078.0	624.1	601.9	524.1	4468.6	445.3	452.3
2072.0	624.2	601.9	538.0	4435.8	433.1	451.1
2067.3	624.3	601.8	549.6	4404.1	422.9	449.9
2065.7	624.4	601.7	554.4	4382.1	418.7	448.4
2085.3	626.9	601.5	554.3	4372.8	422.2	445.4
2104.1	631.3	601.2	554.3	4369.1	426.2	441.4
2103.0	636.5	601.2	554.2	4369.6	426.1	435.9
2102.0	636.0	601.3	554.1	4376.0	424.4	428.3
	643.41					

ADHD*	Autism	Bipolar	**Conduc**	Depress	Eating D	Intell Disab***
-0.82	-0.35	0.64	0.07	-0.92	-0.49	0.49
-0.41	0.01	0.14	0.31	-0.93	-0.36	0.04
0.50	0.54	-0.67	0.84	-0.89	-0.78	-0.64
0.49	0.48	-0.75	0.83	-0.89	-0.77	-0.67
0.69	0.84	-0.96	0.78	-0.86	-0.67	-0.86
0.95	0.73	-0.93	-0.77	-0.90	0.94	-0.61
-0.81	-0.47	0.71	-0.07	-0.88	-0.36	0.59
-0.40	-0.27	0.35	0.03	-0.77	-0.09	0.28
0.63	0.04	-0.25	0.43	-0.55	-0.39	-0.19
0.66	-0.09	-0.23	0.42	-0.55	-0.38	-0.13
0.68	0.77	-0.90	0.72	-0.80	-0.61	-0.80
0.75	0.45	-0.69	-0.57	-0.73	0.70	-0.45
-0.70	-0.01	0.36	0.40	-0.85	-0.70	0.18
-0.23	0.43	-0.24	0.64	-0.91	-0.66	-0.36
0.27	0.79	-0.83	**0.93**	-0.90	-0.86	-0.84
0.19	0.82	-0.96	**0.92**	-0.90	-0.86	-0.92
0.66	0.87	-0.99	**0.84**	-0.90	-0.74	-0.87
0.95	0.86	-0.99	-0.80	-0.90	0.98	-0.62
-0.88	-0.74	0.94	-0.41	-0.80	-0.13	0.86
-0.81	-0.80	0.93	-0.63	-0.26	0.49	0.87
-0.29	-0.91	0.95	-0.76	0.71	0.66	0.94
0.11	-0.85	0.90	-0.82	0.75	0.79	0.88
-0.05	-0.57	0.80	-0.94	0.92	0.94	0.68
0.12	-0.07	-0.16	0.65	-0.46	-0.30	-0.13

Table 1
Pearson Correlation Coefficients (r) of Comparisons of
Firearm Death Rates and Prevalence of Mental Disorders, 1990-2019,
by Type of Firearm Death, Mental Disorder, and Era, U.S.

Rates and Prevalence Age-Adjusted
Values in red: r > 0.80

Era:	1990-2019	1995-2019	2000-2019	2005-2019	2010-2019	2015-2019
**All Firearm Death Rate**						
All Mental Disorders	-0.83	-0.65	-0.63	-0.66	-0.50	0.35
Anxiety Disorders	-0.63	-0.55	-0.58	-0.61	-0.42	0.31
Depressive Disorders	-0.92	-0.93	-0.89	-0.89	-0.86	-0.90
ADHD*	-0.82	-0.41	0.50	0.49	0.69	**0.95**
Bipolar Disorder	0.64	0.14	-0.67	-0.75	-0.96	-0.93
Conduct Disorder	0.07	0.31	**0.84**	**0.83**	0.78	-0.77
Schizophrenia	-0.61	-0.66	-0.92	-0.94	-0.95	-0.97
Autism Disorders**	-0.35	0.01	0.55	0.50	0.77	0.61
Eating Disorders	-0.49	0.04	-0.78	-0.77	-0.67	**0.94**
Intellectual Disability***	0.49	-0.36	-0.64	-0.67	-0.86	-0.61
**Firearm Homicide Rate**						
All Mental Disorders	-0.75	-0.45	-0.25	-0.21	-0.44	0.28
Anxiety Disorders	-0.53	-0.34	-0.20	-0.14	-0.37	0.25
Depressive Disorders	-0.88	-0.77	-0.55	-0.55	-0.80	-0.73
ADHD*	-0.81	-0.40	0.63	0.66	0.68	0.75
Bipolar Disorder	0.71	0.35	-0.25	-0.23	-0.90	-0.69
Conduct Disorder	-0.07	0.03	0.43	0.42	0.72	-0.57
Schizophrenia	-0.47	-0.40	-0.56	-0.59	-0.89	-0.76
Autism Disorders**	-0.47	-0.27	0.05	-0.07	0.72	0.44
Eating Disorders	-0.36	0.28	-0.39	-0.38	-0.61	0.70
Intellectual Disability***	0.59	-0.09	-0.19	-0.13	-0.80	-0.45
**Firearm Suicide Rate**						
All Mental Disorders	-0.89	-0.79	-0.78	-0.84	-0.57	0.33
Anxiety Disorders	-0.77	-0.73	-0.74	-0.83	-0.49	0.29
Depressive Disorders	-0.85	-0.91	-0.90	-0.90	-0.90	-0.90
ADHD*	-0.70	-0.23	0.27	0.19	0.66	**0.95**
Bipolar Disorder	0.36	-0.24	-0.83	-0.96	-0.99	-0.99
Conduct Disorder	0.40	0.64	**0.93**	**0.92**	**0.84**	-0.80
Schizophrenia	-0.81	-0.85	-0.92	-0.95	-0.98	-0.97
Autism Disorders**	-0.01	0.43	0.79	**0.83**	0.77	0.64
Eating Disorders	-0.70	-0.36	-0.86	-0.86	-0.74	**0.98**
Intellectual Disability***	0.18	-0.66	-0.84	-0.92	-0.87	-0.62

* Attention-deficit/hyperactivity disorder
** Autism spectrum disorders
*** Idiopathic developmental intellectual disability

N = 180

	**Mental Disorders Categories**
1	Mental Disorders
2	Schizophrenia
3	Anxiety disorders
4	Attention-deficit/hyperactivity disorder
5	Autism spectrum disorders
6	Bipolar disorder
7	Conduct Disorder Incidence
8	Depressive disorders
9	Eating disorders
10	Idiopathic developmental intellectual disability

JP Regessions

JP 2	JP1	JP1	JP 2	JP 3				US		Hi SDI s US	
US					Hi SDI s US			Suicide	Homicide	Suicide	Homicide
All Firearm Death:		FA Suicid	FA Homicides		All Firear	FA Suicid	FA Homi	%	%	%	%
10.2	10.3	5.9	3.9	3.8	1.5	0.9	0.4	58%	38%	58%	28%
10.2	10.3	5.9	3.9	3.9	1.4	0.8	0.4	57%	38%	58%	28%
10.2	10.3	5.8	4.0	4.0	1.4	0.8	0.4	57%	39%	59%	28%
10.2	10.2	5.8	4.0	4.0	1.4	0.8	0.4	56%	39%	59%	28%
10.2	10.2	5.7	4.1	4.1	1.3	0.8	0.4	56%	40%	59%	27%
10.2	10.2	5.7	4.1	4.2	1.3	0.8	0.4	55%	40%	59%	27%
10.2	10.2	5.6	4.2	4.2	1.3	0.7	0.3	55%	41%	60%	27%
10.2	10.2	5.7	4.2	4.1	1.2	0.7	0.3	56%	42%	60%	27%
10.2	10.1	5.8	4.0	4.0	1.2	0.7	0.3	57%	40%	60%	27%
10.3	10.1	5.9	3.8	3.9	1.2	0.7	0.3	58%	38%	60%	27%
10.3	10.1	6.0	3.7	3.8	1.1	0.7	0.3	59%	36%	61%	27%
10.3	10.1	6.1	3.5	3.7	1.1	0.7	0.3	61%	35%	61%	26%
10.3	10.3	6.2	3.6	3.6	1.1	0.6	0.3	60%	35%	61%	26%
10.3	10.6	6.3	3.8	3.5	1.0	0.6	0.3	60%	36%	61%	26%
10.3	10.8	6.4	3.9	3.8	1.0	0.6	0.3	60%	36%	61%	26%
10.9	11.1	6.6	4.1	4.1	1.0	0.6	0.3	59%	37%	62%	26%
11.5	11.3	6.7	4.2	4.5	0.9	0.6	0.2	59%	37%	62%	26%
12.1	11.6	6.8	4.4	4.5	0.9	0.6	0.2	59%	38%	62%	26%
12.0	11.9	6.9	4.5	4.5	0.9	0.6	0.2	58%	38%	62%	25%
11.8	12.1	7.0	4.7	4.5	0.9	0.5	0.2	58%	39%	63%	25%
								**59%**	**37%**	**62%**	**26%**
		APC				APC		Avrerage during 2000-2019			
0.1	-0.2	-1.0	1.3	1.6	-2.7	-2.4	-3.3				
5.7	2.3	1.8	-4.7	-2.8							
-1.5			3.9	9.3							
				0.0							
		p-Vlaue				p-Vlaue					
0.46	0.40	0.008	0.18	0.07	0.003	0.10	0.11				
0.02	7E-06	< 10-10	0.19	0.003							
0.47			0.0003	0.03							
				0.12							
