# Supplementary material for: Fallacy of attributing the U.S. firearm mortality epidemic to mental health
Source: PLoS One. 2024 Aug 5;19(8):e0290138. doi: 10.1371/journal.pone.0290138 (PMC11299823; doi:10.1371/journal.pone.0290138)
Supplement: Attachment — Submitted filename: Reviewers Replies.docx [file pone.0290138.s008.docx]

Revision summary

The authors completely agreed with the recommendations. In general we added discussion of other relevant reports in the medical literature, four of which were selected, and removed the discussion on legislative impact and its 3 references). In total the manuscript was actually thus shortened, from 3,375 to 3,378 text words, and the number of references (45) being the same. Also, the figures and supporting information are being separately submitted as *tiff*s.

Reviewer #1:

The authors deserve my sincere congratulations. It has been quite some time since I reviewed such a good paper. The introduction is straightforward , the hypothesis is highly relevant, the data source is reliable, the statistical analysis is simple but efficient, and the discussion is clear and concise.

Reviewer #2:

The manuscript is sound and addresses an important topic. Statistical analysis is also valid. However, it lacks a literature review on the topic.

As also recommended by Reviewer #3, a literature review has been added:

*“Although the primary limitation of this study is its ecologic nature, case control and cohort studies support firearm access per se as a causal factor of firearm mortality and not mental health per se. In the U.S., higher rates of firearm ownership at the state level have been shown to be strongly associated with higher rates of firearm suicide but not with non-firearm suicide or gun ownership level.*^New reference39^ *The authors concluded that firearm ownership rates independent of underlying rates of suicidal behavior largely determine variations in suicide mortality across the 50 states.*^New reference 39^ *In the U.S., a nationally representative study of 10,123 13-18 year-olds estimated that their risk of suicide was increased 3-4 times if they had lived in homes with a firearm compared with if they had not. ^New reference 40^ An analysis of four U.S. studies did not suggest any other confounders that explain the association between firearms and suicide.*^New reference 41^

*Other examples have evidenced that suicide rates can be substantially reduced without targeting underlying mental health or suicidality. The success in preventing suicides in Sri Lanka by reducing access to the most highly toxic pesticides is, as the authors conclude, “one of the strongest empirical arguments” and supports ‘household firearm ownership as a consistent strong predictor of suicide risk in studies that examined individual-level data.*^New reference 42^*”*

New References

39. Miller M, Barber C, White RA, Azrael D. Firearms and suicide in the United States: is risk independent of underlying suicidal behavior? Am J Epidemiol. 2013 Sep 15;178(6):946-55. doi:10.1093/aje/kwt197*.*

40. Swanson SA, Eyllon M, Sheu YH, Miller M. Firearm access and adolescent suicide risk: toward a clearer understanding of effect size. Inj Prev. 2020 May 14;27(3):264–70. doi:10.1136/injuryprev-2019-043605.

41. Miller M, Swanson SA, Azrael D. Are we missing something pertinent? A bias analysis of unmeasured confounding in the firearm-suicide literature. Epidemiol Rev. 2016;38(1):62-9. doi:10.1093/epirev/mxv011.

42: Azrael D and Miller M. Reducing suicide without affecting underlying mental health. Theoretical underpinnings and a review of the evidence base linking the availability of lethal means and suicide. Chapter 35. The International Handbook of Suicide Prevention, 2^nd^ Edition. Edited by Rory C. O’Connor and Jane Pirkis. Published 2016 by John Wiley & Sons, Ltd.. Baffins Lane, Chichester, West Sussex PO19 1UD, England.

Authors should also rephrase some sentences to incorporate passive voice only.

All active voice phrases, which were limited to the Methods section have been rephrased in the passive mode:

*“The mean and 95% confidence interval (C.I.) for mental health disorder were obtained for the U.S. and for other high sociodemographic index (SDI)^4^ countries from the Institute of Health Metrics and Evaluation (IHME) Global Health Burden resource.^5^ For each of nine mental health disorders provided by IHME, the U.S. was compared with each of 40 other high SDI countries for overall firearm deaths, firearm homicides, and firearm suicides. Annual firearm death rate data in the U.S. was obtained from the Centers for Disease Control and Prevention Web-based Injury Statistics Query, Reporting and WONDER Systems.^6,7^ U.S. data were available until 2021 whereas IHME SDI country data were not available after 2019. The Joinpoint Regression Program, version 5.0.1-April 2023^8^ and its constant variance option, was used to identify mortality trends and when they occurred. Joinpoint-derived p-values in the figures are designated in the text as <<0.001 if several logs <0.001.”*

Reviewer #3:

The manuscript under review, “Fallacy of Attributing the U.S. Firearm Mortality Epidemic to Mental Health” is a cogent empirical essay that underscores an observation in need of reinforcement. Namely, that mental disorder prevalence in the U.S. is similar in all major categories to its 40 comparable sociodemographic countries and cannot explain the U.S. firearm crisis. None of this minimizes the need for the U.S. to support overall mental health, as the authors take care to note.

The paper could be strengthened substantially by integrating well-established research in the peer-reviewed literature in the introduction and discussion, such as studies that mirror what the authors present at the international level using within state variation in the US and bias analyses of existing case control studies on the relation between guns and suicide . The straw man use of the NRA detracts from an otherwise sober and thoughtfully written manuscript. Better to cite prior work that has built the case to date and to which this paper adds value.

Some of these papers include:

Azrael D and Miller M. Reducing Access to Lethal Means. A Review of the Evidence Base. Chapter XXVI. The International Handbook of Suicide Prevention, Second Edition. Edited by Rory C. O’Connor and Jane Pirkis. Published 2016 by John Wiley & Sons, Ltd.. Baffins Lane, Chichester, West Sussex PO19 1UD, England.

This may not be the correct reference information since the authors have the following chapter (#36 and not XXVI) in the 2^nd^ edition of the book: *Reducing Suicide Without Affecting Underlying Mental Health. Theoretical Underpinnings and a Review of the Evidence Base Linking the Availability of Lethal Means and Suicide.* This chapter is now cited as summarized below.

Miller M, Swanson S, Azrael D. Are We Missing Something Pertinent? A Bias Analysis of Unmeasured Confounding in the Firearm-Suicide Literature. Epidemiol Rev (2016) 38 (1): 62-69.doi: 10.1093/epirev/mxv011.

Swanson S, Eyllon M, Sheu Y, Miller M. Firearm access and adolescent suicide risk: Toward a clearer understanding of effect size. Injury Prevention. Published Online First: 14 May 2020. doi: 10.1136/injuryprev-2019-043605.

Miller M, Barber C, Azrael D, White R. Firearms and suicide in the United States: is risk independent of underlying suicidal behavior? Am J Epidemiol 2013 Sep 15;178(6):946-55. doi: 10.1093/aje/kwt197. Epub 2013 Aug 23.

All four of these references (new #39-42) have been added to the Discussion as summarized below.

In the discussion the authors focus only on the ecologic literature when making the case that firearms causally contribute to suicide rates. The argument could be strengthened by also referring to the individual-level case control and cohort studies that exist.

The references and additional comments in the Discussion:

*“Although the primary limitation of this study is its ecologic nature, case control and cohort studies support firearm access per se as a causal factor of firearm mortality and not mental health per se. In the U.S., higher rates of firearm ownership at the state level have been shown to be strongly associated with higher rates of firearm suicide but not with non-firearm suicide or gun ownership level.*^New reference 39^ *The authors concluded that firearm ownership rates independent of underlying rates of suicidal behavior largely determine variations in suicide mortality across the 50 states.*^New reference 39^ *In the U.S., a nationally representative study of 10,123 13-18 year-olds estimated that their risk of suicide was increased 3-4 times if they had lived in homes with a firearm compared with if they had not.*^New reference 40^ *An* *analysis of four U.S. studies did not suggest any other confounders that explain the association between firearms and suicide.*^New reference 41^

*Other examples have evidenced that suicide rates can be substantially reduced without targeting underlying mental health or suicidality. The success in preventing suicides in Sri Lanka by reducing access to the most highly toxic pesticides is, as the authors conclude, “one of the strongest empirical arguments” and supports ‘household firearm ownership as a consistent strong predictor of suicide risk in studies that examined individual-level data.*^New reference 42^*”*

New References

39. Miller M, Barber C, White RA, Azrael D. Firearms and suicide in the United States: is risk independent of underlying suicidal behavior? Am J Epidemiol. 2013 Sep 15;178(6):946-55. doi:10.1093/aje/kwt197*.*

40. Swanson SA, Eyllon M, Sheu YH, Miller M. Firearm access and adolescent suicide risk: toward a clearer understanding of effect size. Inj Prev. 2020 May 14;27(3):264–70. doi:10.1136/injuryprev-2019-043605.

41. Miller M, Swanson SA, Azrael D. Are we missing something pertinent? A bias analysis of unmeasured confounding in the firearm-suicide literature. Epidemiol Rev. 2016;38(1):62-9. doi:10.1093/epirev/mxv011.

42: Azrael D and Miller M. Reducing suicide without affecting underlying mental health. Theoretical underpinnings and a review of the evidence base linking the availability of lethal means and suicide. Chapter 35. The International Handbook of Suicide Prevention, 2^nd^ Edition. Edited by Rory C. O’Connor and Jane Pirkis. Published 2016 by John Wiley & Sons, Ltd.. Baffins Lane, Chichester, West Sussex PO19 1UD, England.

I’m not sure that the argument they make when evoking reviews of the effect of legislation advances their argument. The authors should review those paragraphs and ask themselves what if anything does this text support of relevance to their important but modest point.

This Discussion and its 3 associated references regarding legislative advances have been deleted.
